# Modulation of the Metabiome by Rifaximin in Patients with Cirrhosis and Minimal Hepatic Encephalopathy

**DOI:** 10.1371/journal.pone.0060042

**Published:** 2013-04-02

**Authors:** Jasmohan S. Bajaj, Douglas M. Heuman, Arun J. Sanyal, Phillip B. Hylemon, Richard K. Sterling, R. Todd Stravitz, Michael Fuchs, Jason M. Ridlon, Kalyani Daita, Pamela Monteith, Nicole A. Noble, Melanie B. White, Andmorgan Fisher, Masoumeh Sikaroodi, Huzefa Rangwala, Patrick M. Gillevet

**Affiliations:** 1 Division of Gastroenterology, Hepatology and Nutrition, Virginia Commonwealth University and McGuire Veterans Affairs Medical Center, Richmond, Virginia, United States of America; 2 Department of Microbiology, Virginia Commonwealth University, Richmond, Virginia, United States of America; 3 Microbiome Analysis Center, George Mason University, Manassas, Virginia, United States of America; 4 Department of Computer Science, George Mason University, Manassas, Virginia, United States of America; Institute of Medical Research A Lanari-IDIM, University of Buenos Aires-National Council of Scientific and Technological Research (CONICET), Argentina

## Abstract

Hepatic encephalopathy (HE) represents a dysfunctional gut-liver-brain axis in cirrhosis which can negatively impact outcomes. This altered gut-brain relationship has been treated using gut-selective antibiotics such as rifaximin, that improve cognitive function in HE, especially its subclinical form, minimal HE (MHE). However, the precise mechanism of the action of rifaximin in MHE is unclear. We hypothesized that modulation of gut microbiota and their end-products by rifaximin would affect the gut-brain axis and improve cognitive performance in cirrhosis. Aim To perform a systems biology analysis of the microbiome, metabolome and cognitive change after rifaximin in MHE.

**Methods:**

Twenty cirrhotics with MHE underwent cognitive testing, endotoxin analysis, urine/serum metabolomics (GC and LC-MS) and fecal microbiome assessment (multi-tagged pyrosequencing) at baseline and 8 weeks post-rifaximin 550 mg BID. Changes in cognition, endotoxin, serum/urine metabolites (and microbiome were analyzed using recommended systems biology techniques. Specifically, correlation networks between microbiota and metabolome were analyzed before and after rifaximin.

**Results:**

There was a significant improvement in cognition(six of seven tests improved,p<0.01) and endotoxemia (0.55 to 0.48 Eu/ml, p = 0.02) after rifaximin. There was a significant increase in serum saturated (myristic, caprylic, palmitic, palmitoleic, oleic and eicosanoic) and unsaturated (linoleic, linolenic, gamma-linolenic and arachnidonic) fatty acids post-rifaximin. No significant microbial change apart from a modest decrease in *Veillonellaceae* and increase in *Eubacteriaceae* was observed. Rifaximin resulted in a significant reduction in network connectivity and clustering on the correlation networks. The networks centered on *Enterobacteriaceae, Porphyromonadaceae* and *Bacteroidaceae* indicated a shift from pathogenic to beneficial metabolite linkages and better cognition while those centered on autochthonous taxa remained similar.

**Conclusions:**

Rifaximin is associated with improved cognitive function and endotoxemia in MHE, which is accompanied by alteration of gut bacterial linkages with metabolites without significant change in microbial abundance.

**Trial Registration:**

ClinicalTrials.gov NCT01069133

## Introduction

Dysfunction of the gut-liver-brain axis in cirrhosis can manifest as hepatic encephalopathy, the subclinical form of which is minimal hepatic encephalopathy (MHE) [1]. MHE affects several cognitive domains that can adversely impact patients in their daily function [2], [3]. The treatment of MHE using gut-selective strategies can improve cognitive function and quality of life in patients; however the precise mechanisms of their action are not clear [4]–[6]. Rifaximin is a gut-selective antibiotic that has efficacy in the therapy of HE, traveler’s diarrhea and irritable bowel syndrome [7], [8]. The mechanism of action of rifaximin is presumed to modulate the concentration of gut microbiota, which has only been investigated in cirrhosis using culture-based techniques. However the effect of rifaximin on gut flora using culture-independent techniques and its effect on gut-derived metabolites in the improvement of MHE has not been investigated.

With the advent of the Human Microbiome project, there has been substantial focus on characterization of the microbial taxa in the human gut in disease states [9]. It is now apparent that the gut microbiome is highly individualized and is influenced by diet and environmental factors [10]. The resulting taxa abundance data is non-parametric and sparse, that is there are many taxa that are present in one individual that are not present in another. From an ecological perspective, one can hypothesize that this observation could be explained by the proposition that different taxa perform the same function in the gut ecosystem [11]. Thus, there are many discrepancies and confounding observation seen in the current microbiome literature that tries to correlate microbial taxa with clinical conditions such as obesity and inflammatory bowel disease [12], [13]. We propose that one needs to take a systems biology approach to correlate the complex functional dynamic in the gut ecosystem as a modulator of the gut-brain axis in the human host [14].

Thus, the aim of this study was to use a systems biology approach to evaluate the effect of rifaximin therapy on the metabiome which we define as the interaction between the phenome (cognition, liver disease severity and endotoxin), microbiome (stool microbial community) and metabolome (serum and urine metabolites) in patients with cirrhosis and MHE [15]. The *a priori* hypothesis was that rifaximin therapy would improve cognition, reduce endotoxemia, dysbiosis and gut-derived systemic products in patients with MHE.

## Methods

### Overall Trial Design

**Figure 1:**
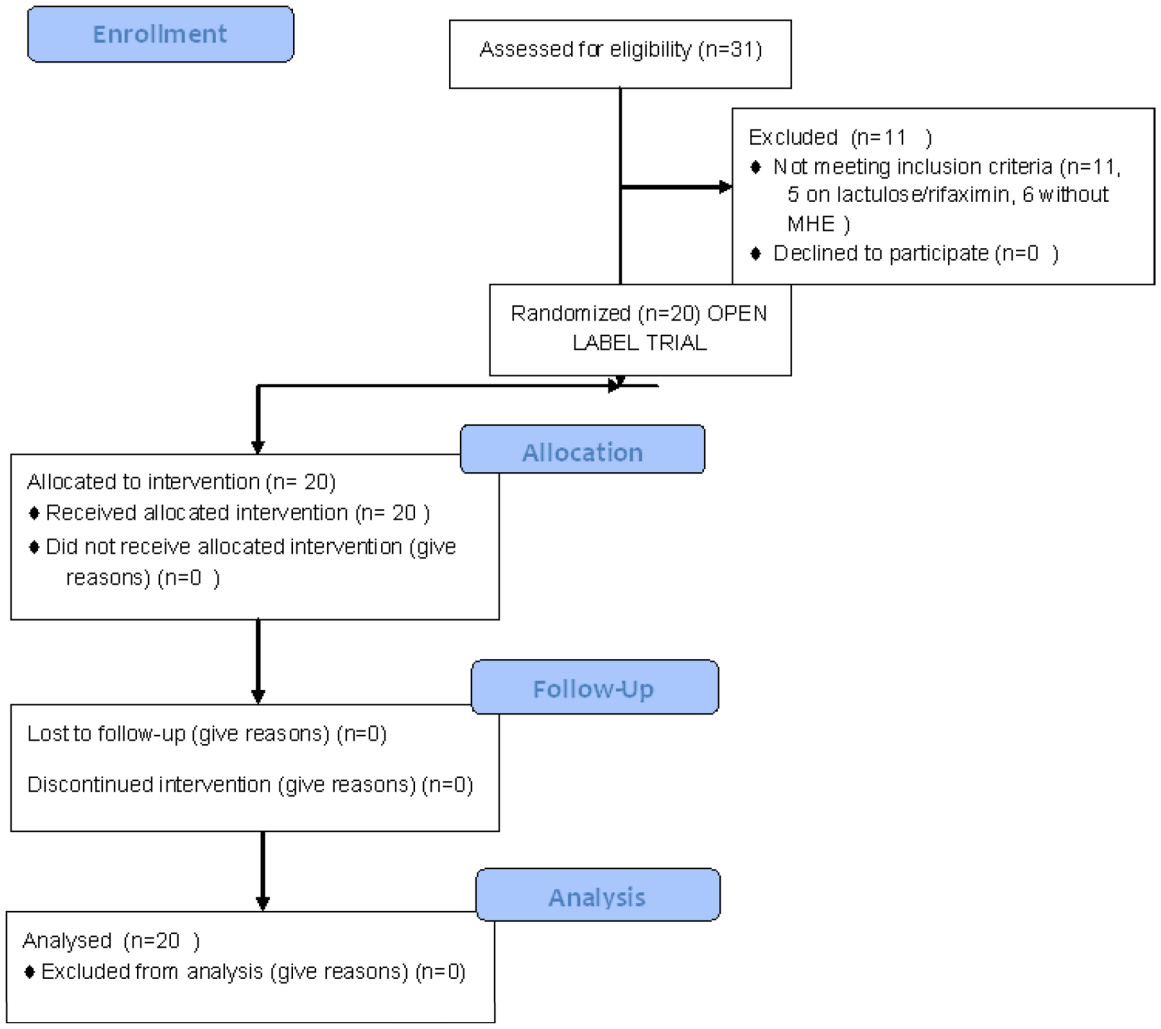
Consort Flowchart of the Open-label trial.

This trial was conducted at the Hunter Holmes McGuire VA Medical Center between April 2010 through March 2012. Patients for this trial were recruited after obtaining written informed consent and underwent all study procedures (Figure 1). The protocol and checklist for this trial are available as supporting information; see SI Protocol and Checklist. We screened 31 patients for this study; five were previously on lactulose/rifaximin and six did not have MHE based on their cognitive performance. We included twenty patients with cirrhosis who had been diagnosed with MHE using our pre-defined criteria [two of the following abnormal compared to our healthy controls, number connection test A/B (NCT-A/B), Digit symbol (DST) and Block Design (BDT)] at least 2 months prior to the start of this trial [1] as has been used and recommended in cirrhosis [16]. We only included patients with cirrhosis between 18–65 years of age, without a prior TIPS placement, without prior overt HE and on treatment for it and those who are able to give written informed consent. Patients were diagnosed with cirrhosis if they had biopsy evidence, radiological evidence or endoscopic evidence of varices). We excluded patients with prior overt HE, who had a recent mini-mental status exam result of <25, those who scored better than the inclusion criteria on the cognitive tests and those with prior TIPS or overt HE. For the first visit, we gave the patients the tests again to confirm the MHE status and to account for any learning effect. The patients were prescribed open-label rifaximin 550 mg PO BID for 8 weeks and the tests were repeated at the end of the study. Subjects were advised to inform the study staff of any adverse events and adherence was assessed at week 8 by the percentage of pills returned.

This report is the microbiome, metabolome and cognitive analysis of this open-label trial that also involved MR imaging of the brain before and after rifaximin. It is registered at www.clinicaltrials.gov number NCT01069133. This trial was conducted under IND number 7,783 granted to Jasmohan Bajaj by the FDA.

### Cognitive Test Battery

We used the following tests at baseline and at the 8 week visit; BDT and the psychometric hepatic encephalopathy score [PHES; consists of NCT-A, NCT-B, DST, line tracing test (LTT; has 2 outcomes; errors and time) and serial dotting (SDT)] which have been validated for use in MHE [17]. Patients also underwent blood draw for MELD score components (serum bilirubin, serum creatinine and INR), serum sodium and venous ammonia at baseline and week 8. A portion of the blood during both visits was centrifuged to produce serum that was stored at −80 degrees C for metabolomic analysis. We also collected 10 ml of urine during both visits that was also stored at −80 degrees C for metabolomic analysis.

### Microbiome Analysis

Fresh stool was collected and DNA extracted for microbiome analysis within 24 h of collection from patients and controls using published techniques. Microbial community fingerprinting and multi-tagged pyrosequencing were performed per published techniques (Text S1) [15]. Metabolomic analysis: Samples were analyzed using GC and LC mass spectroscopy using published techniques (details in Text S1, Methods section) [18].

### Statistical Analysis

Based on our prior microbiome studies [11] we were able to find differences in microbiota constituents between advanced cirrhosis groups with at least 7 subjects in them; we anticipated using 20 patients would be adequate to detect any variation in microbiome in this relatively compensated population. We compared the cognitive performance, MELD score (and its individual components), venous ammonia and endotoxin levels before and after rifaximin using paired t-tests. Clinical and microbiome features of patients before and after rifaximin were compared with a principal coordinate analysis was also used to show differences between the two groups. Only taxa with average abundances >1%, *P* values <0.05, and low *q* values (i.e., low risk of false discovery) were considered significant. Microbiome abundance comparisons between groups were made at a family level using nonparametric tests. A comparison was performed between patients before and after rifaximin using the Wilcoxon matched-pair signed rank tests. All values are presented as means ± SD unless mentioned otherwise.

Metabolomic statistical analyses were performed on all continuous variables using the Statistica DataMiner software version 7.1. Univariate statistical analysis for multiple study design classes was performed by breakdown and one-way ANOVA. F statistics and *p*-values were generated for all metabolites. Data distributions were displayed by box–whisker plots, giving the arithmetic mean value for each category and the standard error as box and whiskers for 1.96 times the category standard deviation to indicate the 95% confidence intervals, assuming normal distributions. Multivariate statistical analysis was performed by unsupervised principal component analysis (PCA) to obtain a general overview of the variance of metabolic phenotypes in the study [19]. In addition, supervised partial least-square (PLS) statistical analysis was performed to obtain information about the variance of metabolic phenotypes that corresponded to the study design classes [20]. Three plots were obtained for each PCA and PLS model. The first was a scree plot for the Eigen values of the correlation or covariance matrix, used as a simple quality check to ensure a steep descent with an increasing number of Eigen values. Second, 2D score scatter plots were generated for at least the first three dimensionless principal components or PLS vectors, and 3D plots were generated to better distinguish metabolic phenotypes if needed. Third, loading plots were generated for each vector in PCA or PLS, showing the impact of variables on the formation of vectors.

The abundances of the bacterial identifications were normalized and taxa present at >1% of the community were tabulated. Unifrac analysis was performed using Version 1.3.0 of Quantitative Insights into Microbial Ecology (QIIME) and weighted P-values were calculated using a Bonferroni correction. Correlation networks were performed separately for groups before and after rifaximin. The microbiome features along with endotoxin, ammonia, and metabolomics were correlated using a Spearman’s correlation function and then filtered for correlations >0.60 and p<0.05. These correlates were calculated using a custom R module, and the correlations and corresponding attributes were imported into Cytoscape for visualization of the network models [21]. The Intersection of the networks was done using the advanced network merge function in Cytoscape. A Correlation Difference (CorrDiff) network was calculated using a R module which extracts edges whose correlations are statistically different between the before and after treatment with a P value <0.05 and where at least one of the original correlations was greater than 0.06 [22], [23]. We then compared the network topology of the network before and after rifaximin to identify which sub-networks were present in one and not the other, giving us clues on system functionality [24]. It is assumed that correlations present in one treatment group that are missing in another not only differentiate the groups but indicate potential clues to the functionality of the system, leading the way to hypothesis-driven experimental research.

## Results

### Rifaximin Trial

All patients were able to complete the trial with rifaximin 550 mg BID for 8 weeks. The overall compliance with the medication was 92%. We included 20 patients, 14 men and 6 women with a mean age of 59.7±3.5 years and education of 14±1.7 years. The majority was Caucasian (14, 70%) with the remainder being African American (6, 30%). The predominant etiology was hepatitis C (7, 35%), followed by alcohol+hepatitis C (4, 20%), non-alcoholic fatty liver disease (4, 20%), alcohol alone (3, 15%) and others (2, 10%). There was a significant improvement in serum bilirubin but not the other MELD score components at the end of the trial (Table 1). There was also a significant improvement in cognitive performance on all tests apart from the block design test compared to the pre-treatment baseline. There was a significant reduction in endotoxin levels after rifaximin therapy compared to baseline (0.55±0.21 vs. 0.48±0.24 Eu/ml, p = 0.02).

**Table 1:** Changes in cognition and cirrhosis severity with rifaximin therapy.

N = 20	Baseline	After rifaximin
MELD score	9.8±3.3	9.4±3.1
INR	1.2±0.2	1.2±0.2
Serum creatinine (mg/dl)	0.9±0.1	0.9±0.2
Serum bilirubin (mg/dl)	1.3±0.8	1.1±0.7*
Serum sodium (meq/L)	138.1±2.8	138.9±2.7
Venous ammonia	46.2±23.4	42.9±23.1
Cognitive tests		
Number connection-A (seconds)	42.3±13.4	37.3±8.9*
Number connection-B (seconds)	97.2±31.9	85.7±25.8*
Digit symbol (raw score)	50.0±12.3	55.1±13.9*
Block design (raw score)	25.9±11.9	28.5±9.6
Line tracing time (seconds)	121.7±32.1	96.4±33.1*
Line tracing errors (number)	41.2±28.3	24.8±17.1*
Serial dotting (seconds)	69.6±25.7	61.0±17.3*

### Microbiome Changes

**Figure 2:**
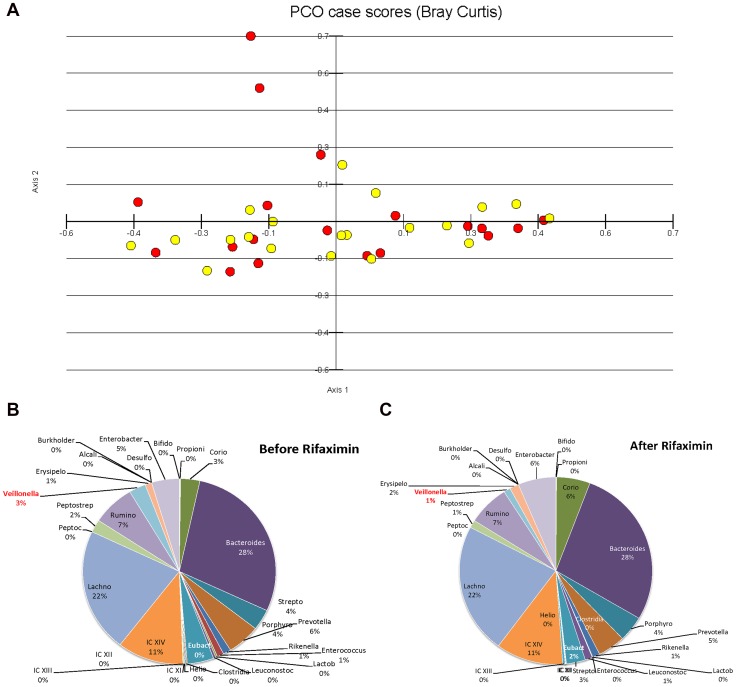
A: Principal Component Analysis of Microbiota. There was no significant change in the PCO of microbiota before and after rifaximin therapy (yellow dots are before and red dots are after rifaximin) B and C: Composition of microbiota families before (figure 2B) and after (figure 2C) rifaximin. There was a significant decrease in *Veillonellaceae* and increase in *Eubacteriaceae* abundance after rifaximin therapy (marked in red).

There was no significant difference in the overall microbiome composition before and after rifaximin upon visual inspection of the principal component analysis (Figure 2A). UNIFRAC PCO analysis also did not show a significant clustering between the microbiota composition before and after rifaximin (Figure S1). However, the UNIFRAC Bonferroni corrected, weighted significance test for the treatments was indicated a slight difference between the microbiome compositions (p = 0.01) There was a significant reduction in the abundance of the taxa *Veillonellaceae* (p = 0.025) and increase in the abundance of *Eubacteriaceae* (p = 0.042) using Metastats but no other significant changes in the microbiome abundance were observed after rifaximin therapy (Figures 2B and 2C).

### Metabolome Analysis

**Figure 3:**
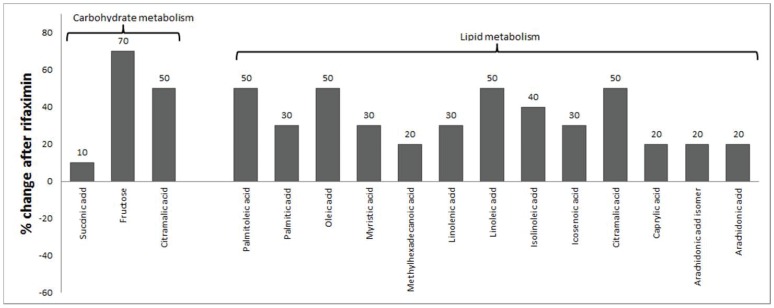
Univariate serum metabolomic analysis. There was a significant increase in fatty acids and intermediates of carbohydrate metabolism after rifaximin therapy in the serum.

There was a significant difference in serum and urine metabolites between groups before and after rifaximin using Partial Least Squares Discriminant Analysis (PLS-DA) (figures S2A and B). Uni-variate analysis of serum metabolites (Figure 3) showed that the majority of the differentiators were serum fatty acids that increased after rifaximin therapy. The pattern of fatty acid increase after rifaximin were a higher level of saturated fatty acids [caprylic (8∶0), myristic (14∶0) and palimitic acid (16∶0)] which after the action of stearoyl-CoA desaturase can be turned into palmitoleic acid (16∶1n7), oleic (18∶1n9) and eicosanoic acid (20∶1n9). An increase in linolenic acid (18∶3n3), gamma-linolenic acid, linoleic (18∶2n6) and arachidonic acid (20∶3n6) formed by the action of delta 6-desaturase and fatty acid elongase was also seen. There was also an increase in serum fructose, succinic acid and citramalic acid after rifaximin. The only significant uni-variate change in urine metabolites was a minor increase in urine succinic acid.

### Correlation Network Analysis

**Figure 4:**
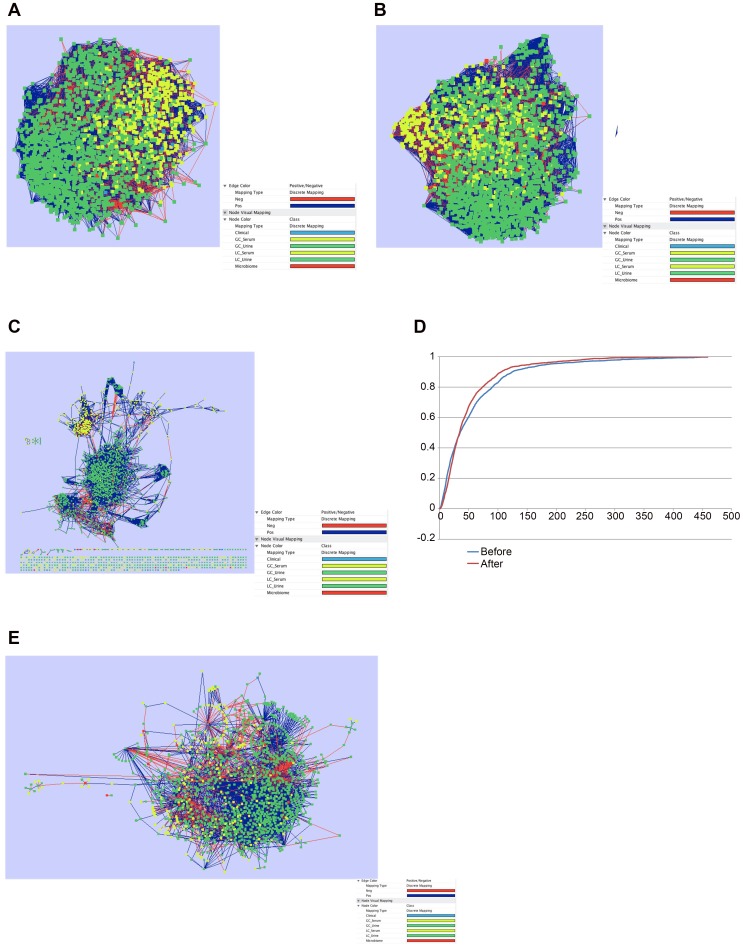
Correlation networks before and after rifaximin. Legend common for figures 4A, 4B and 4C: The complex correlation network represented parameters that were linked with a correlation coefficient >0.6 (negative or positive) and with a p value <0.05. Red nodes represent bacterial taxa, green ones the serum metabolites, yellow nodes indicate urinary metabolites while blue ones indicate clinical parameters. Red edges represent negative correlation between connected nodes and blue edges indicate positive correlations. A: Correlation network before rifaximin (BCN) with r>0.6 or <−0.6 and p<0.001. B: Correlation network after rifaximin (ACN) with r>0.6 or <−0.6 and p<0.001. C: is the intersection of 5A and B. It demonstrates those nodes and correlations that remain exactly same before and after rifaximin. D: Cumulative Degree Function curve. This graph plots the cumulative degree function of the node frequency distributions before and after rifaximin. It shows that after rifaximin therapy there was a significant reduction in network complexity (p<0.0001). Blue line: before and red line: after rifaximin. E: Correlation difference before and after rifaximin. This figure shows the correlations that significantly changed between the before and after rifaximin state; i.e. if two nodes were connected positively in the before rifaximin network but aftr rifaximin changed to negative, they are represented here. While the color coding of the nodes is similar, red edges demonstrate linkages that were positive in the BCN but became negative in ACN, while blue edges represent correlations that changed from negative to positive after the use of rifaximin.

We ran the Spearman correlation network analysis on the 2,238 features in the dataset (Table S1) and selected correlation for both “Before” and “After” treatment that had an absolute Spearman Correlation Coefficient greater than 0.6 and P-value <0.05 The global correlation networks are very complex with 153,000 correlations (2,220 nodes) for the “before” correlation network (BCN) (Figure 4A) and 57,249 correlations (2,225 nodes) for the “after” correlation network (ACN) (Figure 4B).

We calculated the intersection correlation network (ICN) which plots all the correlations that are the same in both the BCN and ACN (Figure 4C). Interestingly, over 99% of the features in the dataset are found in the intersection correlation network. Thus, this intersection correlation network delineates the stable core metabiome of the cirrhotic state that didn’t change during treatment. Visually, there is a major hub of urine metabolites with a minor hub of serum metabolites connected by various minor clusters.

The complexity of the networks is expected as many compounds will be in the same or complementary metabolic pathway. The networks are visually different and this is reflected in the connectivity measurements (Table 2). For example, the average number of neighbors for the BCN is 59 while it is 51 for the ACN. These parameters indicate that rifaximin has a major effect of the metabolic network, reducing a number of the metabolic interactions and reducing the clustering, while keeping the nodes themselves intact.

**Table 2:** Comparison of network topology before and after rifaximin.

	Before Rifaximin	After Rifaximin	Intersection of the two networks
Number of Nodes	2220	2225	2219
Isolated Nodes	0	0	511
Connected Components	1	1	547
Average Number of Neighbors	59.0405405	51.4588764	13.5205047
Network Density	0.02660682	0.02313798	0.00609581
Clustering Coefficient (saturation of the nodes)	0.36257932	0.33746817	0.31452636
Network Diameter (largest distance between nodes)	6	6	15
Network Radius (shortest distance between nodes)	4	4	1
Characteristic Path Length (expected distance between two nodes)	2.77271111	2.75946771	4.68771603
Network Centralization	0.23453281	0.18386182	0.15184087
Shortest Path (shortest path through all nodes)	4926180	4948400	2600364
Network Heterogeneity (tendency to form hubs)	1.19030892	1.05451615	1.80374813

Intersection indicates the nodes and network common to both before and after rifaximin. The table shows that the majority of nodes involved were common (intersection) between the groups while the network density (average number of neighbors and network density) changed after rifaximin therapy. While the diameter and radius remained same, there was a reduction in the path length and heterogeneity after rifaximin compared to before. There was also a decrease in network centralization which means that the distribution was spread out after rifaximin therapy compared to before.

When we plotted the Cumulative Distribution Function (CDF) of the node degree frequency(14), we found that the connectivity simplified after rifaximin (Figure 4D) and this was a statistically significant shift (P<0.001). We found that most of the nodes included in the BCN and ACN are contained in the ICN [2219 nodes] but it contains a much smaller subset of the correlations with an average number of neighbors of 13.5. Thus, despite most of the features being present before and after rifaximin therapy, the connectivity changed significantly after rifaximin. This is in contrast to a much more minimal effect on the bacterial abundances of the microbiome. This implies that rifaximin, which is a bacterial RNA polymerase inhibitor, does not seem to alter the relative bacterial abundances but does promote a major shift in the complexity of the peripheral metabiome network implying a shift in the gut microbiome functionality.

We then calculated the Correlation Difference network (CorrDiff) (Figure 4E) which is a global view of which correlations changed significantly after treatment with rifaximin. We selected only correlation differences that had a Pvalue <0.05 and where at least one of the original Spearman correlation was greater than 0.6. This network contains only 1490 features, which is substantially smaller than the original BCN with only 67% of the original features. Visual inspection of the CorrDiff (Figure 4E) shows a more complex network of hubs that is reflected in the Network Heterogeneity (2.012) and Clustering Coefficient (0.79).

Additionally, one can see that a number of correlations involving bacteria (red squares) were changed by the rifaximin treatment Figure 4D). We found five bacterial taxa (*Enterobacteriaceae, Bacteroidaceae, Veillonellaceae, Porphyromonadaceae* and *Rikenellaceae*) that showed a significant difference in correlations before rifaximin compared to after rifaximin using the correlation difference network. Subnets centered on these taxa from the global BCN and ACN were then visualized (figures S3–S7 in File S1, Text S1). To aid in interpretation, the nodes that were “unassigned” or not yet identified were removed from correlation networks unless they served as a bridge between two named features in the subnets.

### Correlation Differences before and after Rifaximin Therapy

**Figure 5:**
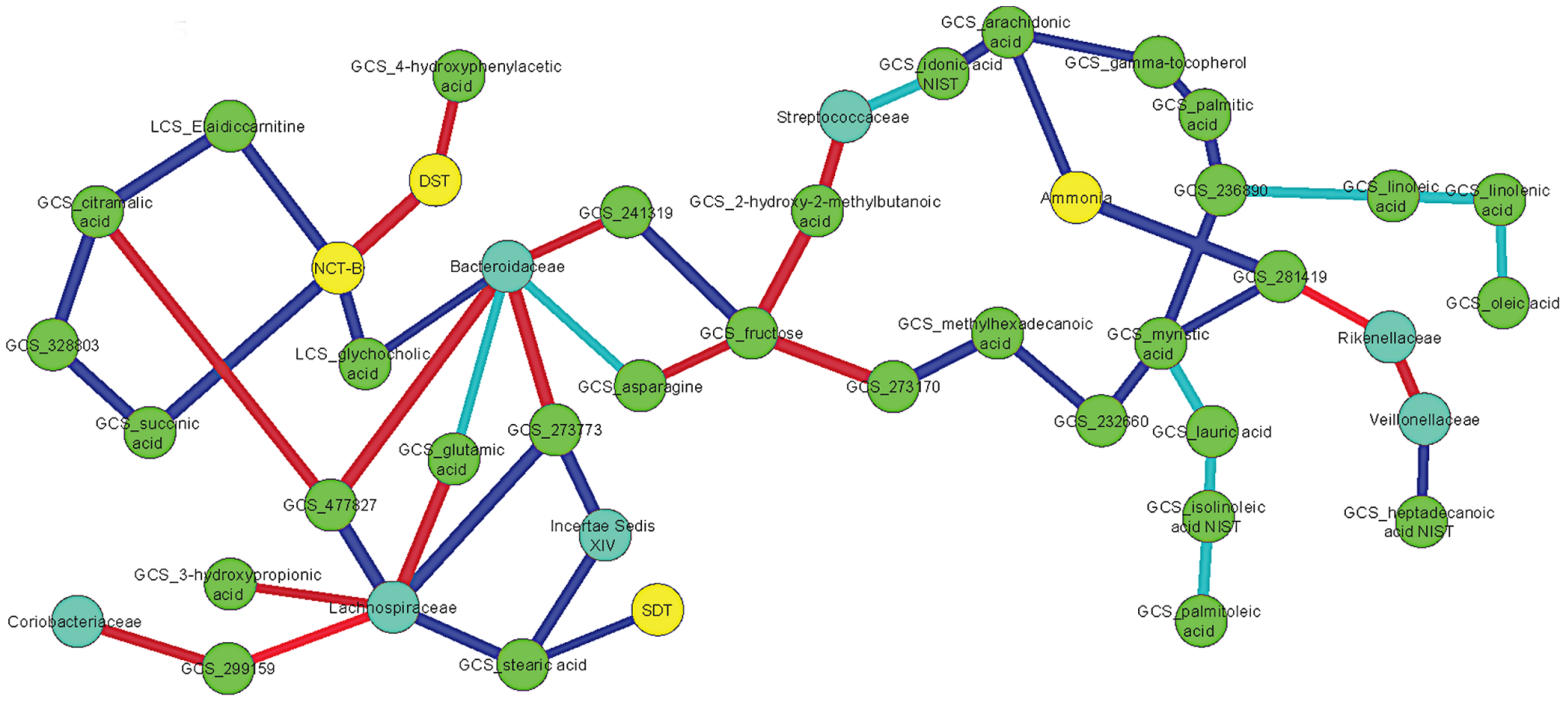
Subset of correlation differences before and after rifaximin. This figure is limited to the metabolomics and clinical/cognitive features that changed with rifaximin and their interaction with the bacterial taxa. The linkages that significantly changed in nature (positive to negative or vice-versa) or intensity (less to more or vice-versa while remaining positive or negative) with p<0.05 are shown. Nodes: Blue: bacterial taxa, green: serum metabolites, Yellow: cognitive or clinical data. Linkages were dark blue if correlations were positive before and changed significantly to negative, light blue if they changed significantly but remained positive throughout, red if correlations were negative at baseline but changed to positive after therapy and green is negative relationship throughout but a significant change.

To identify relationships that changed significantly between baseline and post-rifaximin, we specifically analyzed data on microbiome, significantly different serum metabolites, and clinical/cognitive data (Figure 5). We found that *Bacteroidaceae* changed their linkages from being positively correlated with NCT-B (indicates poor cognition) and glycocholic acid before to a negative correlation after; also there was a reduction in intensity of the positive correlation with glutamic acid and asparagine, both ammonia sources after rifaximin. Glutamic acid changed from negative to positive with *Lachnospiraceae*. We also found that in the network, serum fatty acids (linoleic, linolenic and oleic, and isolinoleic, lauric, myristic and palmitoleic acids) remained correlated with each other positively while the arachidonic acid was initially positively but then negatively linked to ammonia after rifaximin. A high score on SDT indicates poor cognition so it is also interesting that stearic acid, changed its linkage from positive to negative with that cognitive test as well as with autochthonous taxa *Lachnospiraceae* and *Incertae Sedis XIV.* These correlation differences are key in evaluating the potential effects of rifaximin on cognition.

## Discussion

This clinical trial demonstrates that rifaximin is associated with improved cognitive performance and reduction in endotoxemia in patients with cirrhosis and MHE. This was associated with a modest change in the stool microbiota characterization with reduced *Veillonellaceae* and increased *Eubacteriaceae*. There was a significant change in the serum metabolome with a specific increase in serum fatty acids after rifaximin therapy. Correlation networks showed that key bacterial families, *Porphyromonadaceae*, *Bacteroidaceae* and *Enterobacteriaceae* had differing associations with the metabolome and microbiome after rifaximin compared to baseline linkages.

The use of rifaximin for MHE therapy is an attractive proposition due to its efficacy, tolerability and gut-specific action [4], [5], [7]. *In vitro* the rifaximin is able to act on a wide variety of gram-positive and negative organisms [8]. However there is emerging evidence that its primary mode of action may be related to a change in bacterial function and virulence rather than a simple reduction in bacterial population. Studies in *Escherichia coli* and *Shigella sonnei*, virulent members of *Enterobacteriaceae* have shown that rifaximin exposure results in a reduction in its virulence and ability to adhere to intestinal cells while keeping the counts comparable to baseline [25], [26]. Also fecal microbiota studies from Crohn’s disease patients have shown that there was a change in bacterial end-products such as short-chain fatty acids and alcohols, after rifaximin therapy, rather than an absolute difference in numbers [27].

Confirming and extending these investigations into cirrhosis and MHE, we did not find an overall significant change in microbiota composition at the phylum or order level but there was a modest reduction in *Veillonellaceae* and a trend towards increased *Eubacteriaceae*. *Veillonellaceae* are anerobic gram-negative cocci that have been found to be higher in abundance in cirrhotic patients’ stool and colonic mucosa compared to healthy controls [28]. Also, prior studies show that their abundance is greater in the colonic mucosa of cirrhotics with HE compared to those without HE and lesser in those on rifaximin and lactulose compared to lactulose alone [29]. Veillonella spp have also been over-represented in patients with irritable bowel syndrome, most often those with a mixed or constipation-predominant clinical picture [30]. The presence of *Veillonellaceae* may be a marker for the presence of HE and MHE in the stool and colonic mucosa of cirrhotic patients [11], [29]. Since the major fermentative substrate for *Veillonella* is lactic acid, it often has a symbiotic relationship with taxa such as *Streptococcaceae* whose end-product of metabolism is lactate. There was a trend towards reduction in *Streptococcaceae* with rifaximin which could also factor into the reduction of *Veillonellaceae* in this population. Complementary to our findings, *Eubacteriaceae* often respond in directions opposite to that of *Veillonellaceae* after dietary manipulation [31].

There was also a change in the overall network connectivity before and after rifaximin. This is interesting because it shows that although the network features, i.e. microbiota and metabolome are present in the networks before and after rifaximin, rifaximin therapy decentralized and reduced clustering and decreased the overall complexity of the connections between the same nodes. This supports a change in bacterial metabolic function rather than a change in bacterial numbers as the probable cause of its action. When we specifically studied families that have been associated with cirrhosis in the network, a significant change in connectivity before and after rifaximin was observed with respect to metabolites. Prominent among them were changes in *Bacteroidaceae*, *Enterobacteriaceae,* and *Porphyromonadaceae*. All three taxa have been associated with cirrhosis, HE and cognitive dysfunction in the past and include several pathogenic genera such as *Escherichia, Shigella, Alistipes, Porphyromonas, Bacteroides* and *Salmonella* [11], [28], [32]. At baseline, these taxa were correlated with products of aromatic amino acid and ammonia metabolism and oxidative stress indicators [28], [33]. These correlations changed to become beneficial, i.e. negatively correlated with oxidative stress or with aromatic amino acid and nitrogen metabolism after rifaximin. Interestingly, there was also evidence of several quorum sensors in the correlation network that can influence the number of co-existent microbiota, especially those belonging to *Enterobacteriaceae* [*34*]. We also were able to confirm that the autochthonous taxa continued to be beneficially linked to metabolites before and after rifaximin therapy [29]. While correlations do not imply causality, as a whole the network analysis indicates that the associations between metabolites and microbiota changed after rifaximin therapy into a potentially beneficial metabiomic milieu for the host.

Lipopolysaccharide (endotoxin) was significantly reduced in patients taking rifaximin, in the current study consistent with previously reported studies of HE and cirrhosis patients [35]. Moreover, there was a significant increase in serum long-chain fatty acids in patients on rifaximin as compared to controls. The absorption of endotoxin and long-chain fatty acids are believe to primarily occur in the small bowel as bile salts are important for the solubilization of hydrophobic compounds. Both endotoxin and long-chain fatty acid are transported packaged in chylomicrons which are formed in enterocytes in the small bowel [36]. Therefore, we hypothesize that main effect of rifaximin may be to inhibit bacterial growth and reduce endotoxin absorption in the small bowel. This is consistent with the reduction of members of the family *Veillonellaceae*, which are Gram-negative anaerobic cocci and have been reported to be in relatively high numbers in the human ileum [37].

After rifaximin therapy, there was an increase in long-chain saturated fatty acids along with products of stearoyl CoA desaturase. We also found a significant increase in unsaturated fatty acids with higher linoleic, conjugated linoleic, linolenic and arachidonic acids after the treatment with rifaximin. This specific fatty acid profile is interesting because animal studies have shown that it is possible to modify the adipose tissue and peripheral fatty acid profile with introduction of probiotics or bacteria that have specific fatty acid enzyme mutations [38]. Also these studies found that these changes in peripheral fatty acid changes can benefit brain fatty acid constitution in these animals giving a potential mechanism for the biological effect of gut bacteria on brain function [39]. This increase is unlikely to be dietary since the diet remained constant throughout the study and there was no change in the mead acid level, which is a marker for dietary intake change between the pre and post-rifaximin profile [40]. Therefore the increased fatty acids are likely either due to an enhanced transport from the gut to the bloodstream via the thoracic duct as chylomicrons or enhanced release from the adipose tissue. Gut microbiota can affect adipose tissue and peripheral lipoprotein lipase by modulating the fasting-induced adipose factor [41], [42]. The lack of short-chain fatty acids, which are major end-products of bacterial fermentation, in this serum profile is likely because the majority of their biological activity occurs within the gut lumen and they are directly absorbed and transported into the liver [43]. The predominance of long-chain serum fatty acids in the post-rifaximin profile supports the gut-based transport of these molecules in chylomicrons as a potential mechanism for their higher levels. Prior studies have shown that fatty acids, both saturated and unsaturated, are associated with brain function in animal, human and population-based studies[39], [44]–[46]. The brain fatty acid profile impacts neurogenesis, cognition and memory possibly by affecting neurotransmission, axonal sheath composition and cell membrane fluidity. Fatty acids increased in our study, arachidonic and linoleic acids, have been shown to influence brain function directly [44], [46]. There was a significant improvement in their cognitive function after rifaximin therapy across most cognitive domains. While our study did not evaluate mechanisms, it can be speculated that changing the gut bacterial end-product and fatty acid profile could benefit this cognitive ability by potentially affecting the brain fatty acid profile.

We observed that rifaximin does not alter the relative bacterial abundances but does promote a major shift in the complexity of the metabiome network. However, we did not measure absolute abundances of the taxa in the microbiome. Thus, there is a possibility that rifaximin may have had an impact on total microbial mass in the gut that may also play a role in the modulation of the metabiome to improve MHE.

A limitation of this study was that the majority of the metabolome features were unidentified due to limitations in the GC and LC MS databases. Future studies should focus on delineating those metabolites that are at key nodes in the major network hubs of the CorrDiff or are key links between metabiome components. The fecal metabolome and small bowel microbiota analysis could have given additional insight into the mechanism of action of rifaximin. Our sample size was also limited, which could have potentially impeded our ability to characterize changes in microbial abundance, since there is considerable inter-individual microbial variability. Also our study is limited by the evaluation of fecal bacteria, which could have differing abundances from that of the colonic and small bowel mucosal bacteria [29]. However, given these limitations, we were still able to detect changes that are physiologically plausible and relevant to the ultimate result which is improvement in cognition and reduction in endotoxemia after rifaximin therapy.

We have demonstrated that a systems biology approach using network correlation analysis and correlation difference analysis was much more informative in interpreting the interaction of the metabiome (i.e. phenome, microbiome and metabolome) than current multivariate analyses. We postulate that the metabiome is a complex non-linear dynamic and interrogating this dynamic with one time point cannot completely capture the fluctuation in the metabolic network. Additionally, microbiome identification alone may have limited utility in that many taxa may have the same metabolic function in the gut ecosystem. Thus, methodology interrogating functional aspects of the metabiome, such as the metabolome and metatranscriptome, should prove more informative.

We conclude that rifaximin therapy has a systemic and local effect on the microbiota, metabolome, endotoxemia and cognition in patients with minimal hepatic encephalopathy. A significant improvement in cognition with reduction in endotoxemia was observed with a modest change in stool microbiota composition. There was a significant increase in serum long-chain fatty acids after rifaximin therapy. We also found a significant linkage of bacterial taxa with the metabolites, especially those linked to ammonia, aromatic amino acids and oxidative stress, which shifted to reflect changes in bacterial metabolic function after rifaximin therapy. Therefore the mechanism of action of rifaximin, based on our results, may be associated with changing microbiota-associated metabolic function leading to cognitive improvement.

### Ethics Statement

All research involving human participants was approved by Hunter Holmes McGuire VA Medical Center Institutional Review Board. Written informed consent, as approved by the Institutional Review Board, was obtained and all clinical investigations were conducted according to the principles expressed in the Declaration of Helsinki.

## Supporting Information

Figure S1 **Unifrac PCO Analysis: no significant difference was seen before (red) and after rifaximin (blue) therapy with respect to microbiome**
(PDF)

Figure S2 **PLS-DA shows significant separation between before and after rifaximin on urine and serum metabolites.**
(PDF)

Table S1 **List of individual metabolites and other features included in the correlation network.**
(PDF)

Text S1 **Includes Supplementary Methods (Pages S1–6), Supplementary Discussion (pages S7–9), Supplementary References (Page S10) and Supplementary Figure and Table Legends (page S11).**
(DOCX)

File S1 **Figures S3–S7 with individual correlation networks centered before and after rifaximin around Figure S3:** ***Bacterioidaceae*** **, Figure S4:** ***Porphyromonadaceae*** **, Figure S5:** ***Enterobacteriaceae*** **, Figure S6:** ***Veillonellaceae*** **, Figure S7:** ***Rickenellaceae.***
(PDF)

Protocol S1 **Trial Protocol**
(DOC)

Checklist S1 **CONSORT Checklist.**
(DOC)

Listing S1 **Clinical trials registration.**
(PDF)
