# Supplementary figures and images for: Modulation of the Metabiome by Rifaximin in Patients with Cirrhosis and Minimal Hepatic Encephalopathy
Source: PLoS One. 2013 Apr 2;8(4):e60042. doi: 10.1371/journal.pone.0060042 (PMC3615021; doi:10.1371/journal.pone.0060042)

Figure S1

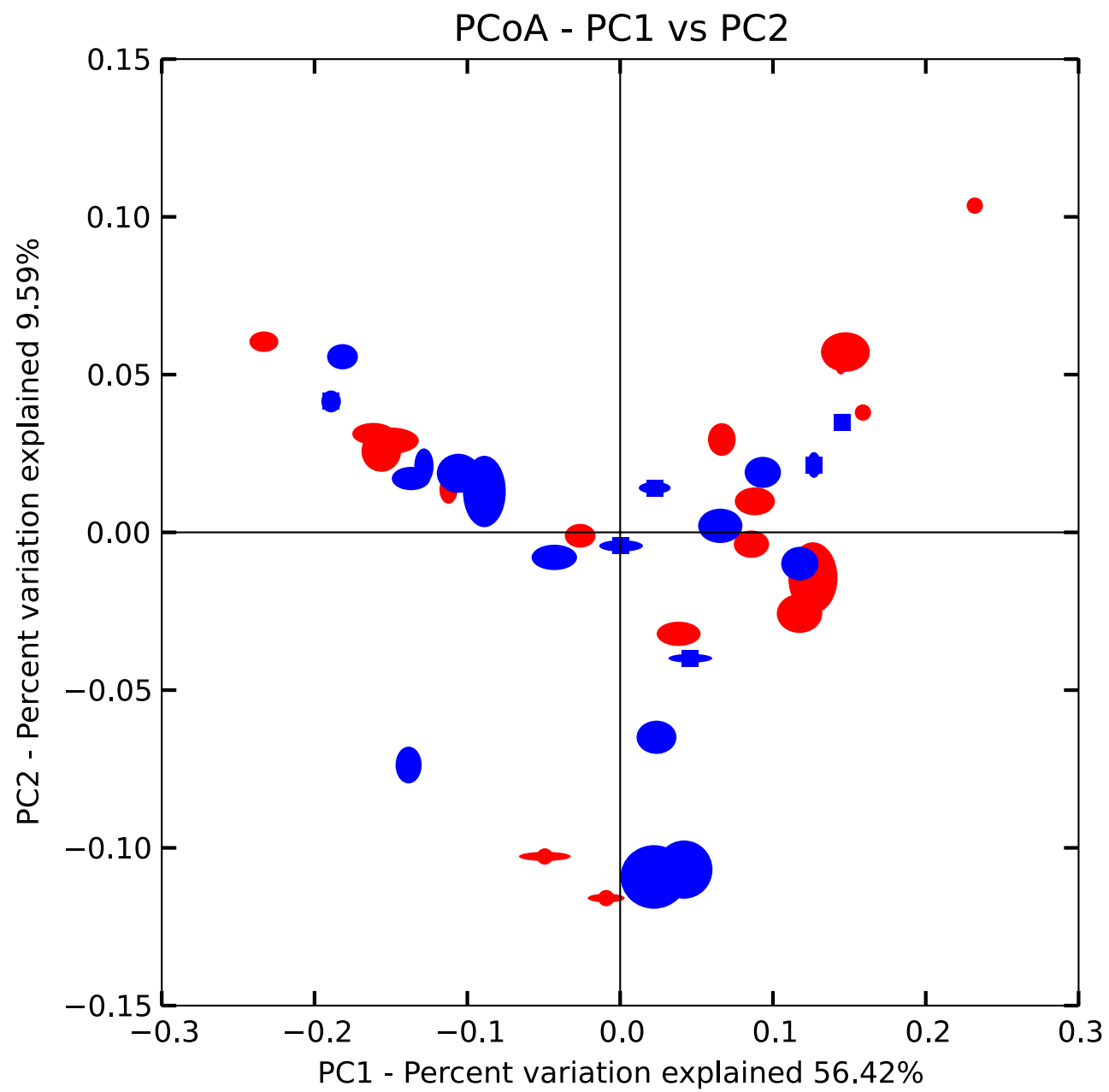

Supplement: Figure S1 — Unifrac PCO Analysis: no significant difference was seen before (red) and after rifaximin (blue) therapy with respect to microbiome (PDF) [file pone.0060042.s001.pdf]

## S2A: Urine Partial Least Square analysis Discriminant Analysis

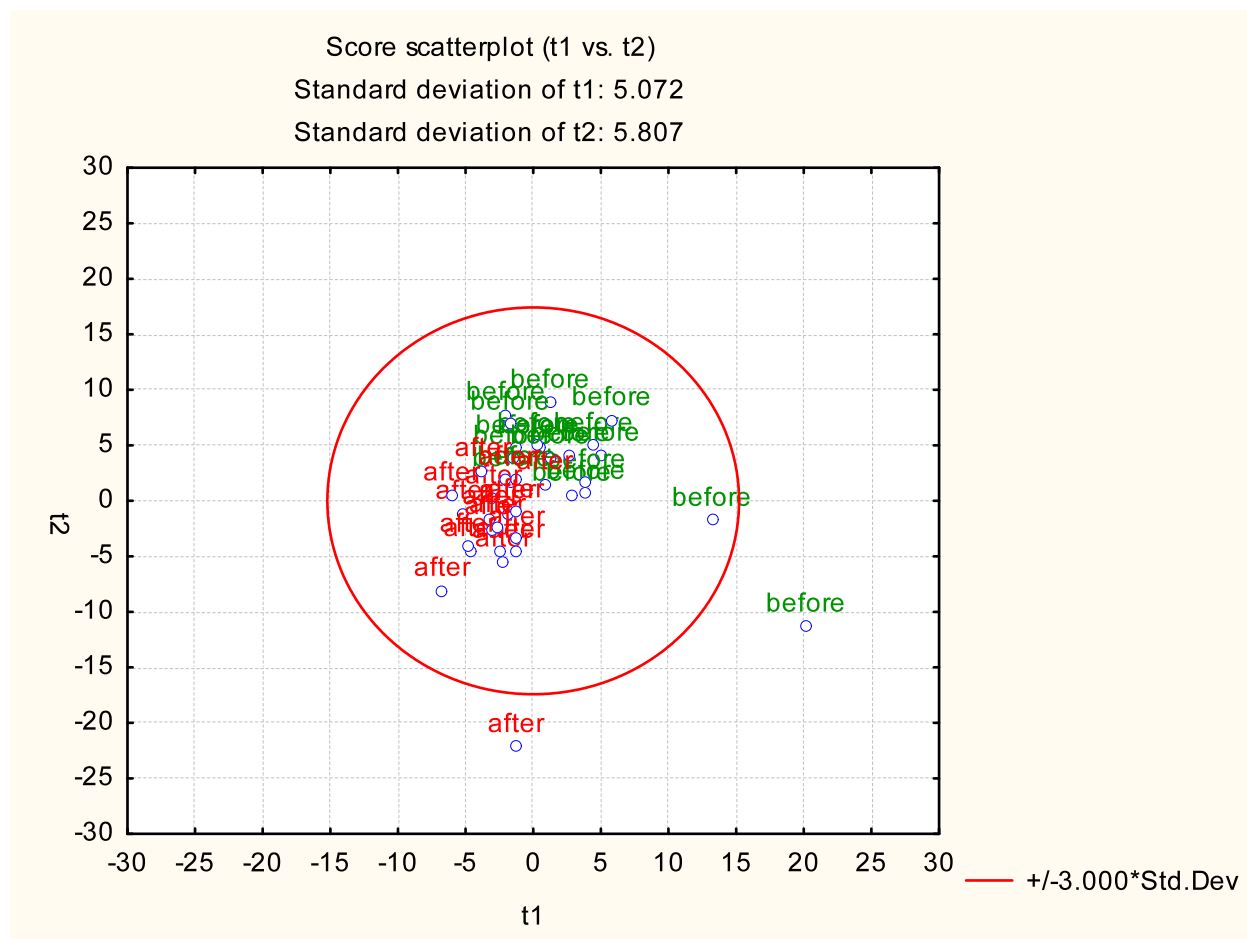

## S2B Serum PLS-DA before and after

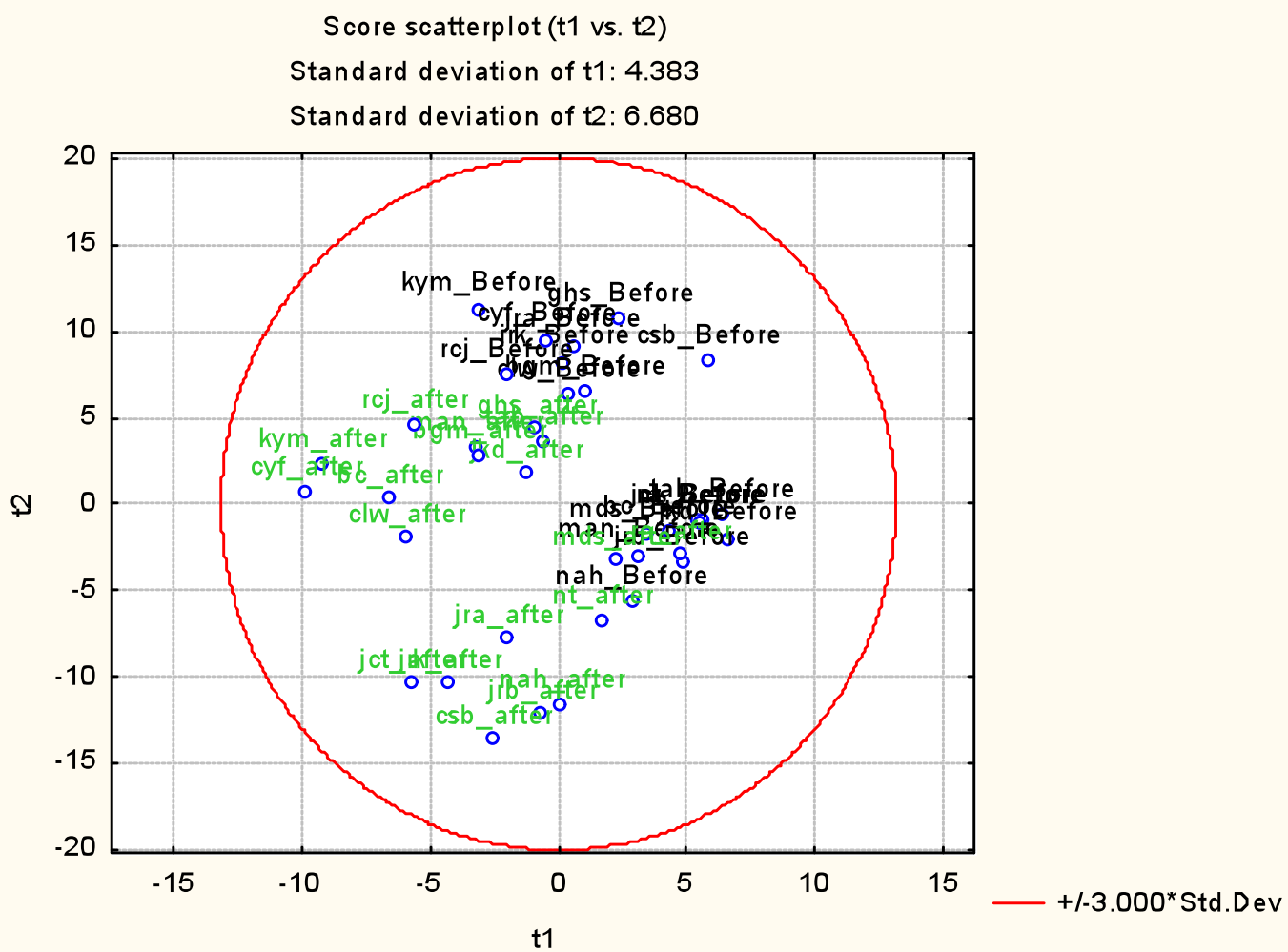

Supplement: Figure S2 — PLS-DA shows significant separation between before and after rifaximin on urine and serum metabolites. (PDF) [file pone.0060042.s002.pdf]
