# Supplementary material for: Modulation of the Metabiome by Rifaximin in Patients with Cirrhosis and Minimal Hepatic Encephalopathy
Source: PLoS One. 2013 Apr 2;8(4):e60042. doi: 10.1371/journal.pone.0060042 (PMC3615021; doi:10.1371/journal.pone.0060042)
Supplement: Table S1 — List of individual metabolites and other features included in the correlation network. (PDF) [file pone.0060042.s003.pdf]

Table S8

Index	Key1	Key2	Class
12	Endotoxin	Endotoxin	Clinical
13	Taxa_13	Actinobacteria_Actinobacteria_Actinomycetales_Actinomycetaceae	Microbiome
14	Taxa_14	Actinobacteria_Actinobacteria_Bifidobacteriales_Bifidobacteriaceae	Microbiome
15	Taxa_15	Actinobacteria_Actinobacteria_Coriobacteriales_Coriobacteriaceae	Microbiome
16	Taxa_16	Bacteroidetes_Bacteroidia_Bacteroidales_Bacteroidaceae	Microbiome
17	Taxa_17	Bacteroidetes_Bacteroidia_Bacteroidales_Porphyromonadaceae	Microbiome
18	Taxa_18	Bacteroidetes_Bacteroidia_Bacteroidales_Prevotellaceae	Microbiome
19	Taxa_19	Bacteroidetes_Bacteroidia_Bacteroidales_Rikenellaceae	Microbiome
20	Taxa_20	Firmicutes_Bacilli_Bacillales_Staphylococcaceae	Microbiome
21	Taxa_21	Firmicutes_Bacilli_Lactobacillales_Carnobacteriaceae	Microbiome
22	Taxa_22	Firmicutes_Bacilli_Lactobacillales_Enterococcaceae	Microbiome
23	Taxa_23	Firmicutes_Bacilli_Lactobacillales_Lactobacillaceae	Microbiome
24	Taxa_24	Firmicutes_Bacilli_Lactobacillales_Leuconostocaceae	Microbiome
25	Taxa_25	Firmicutes_Bacilli_Lactobacillales_Streptococcaceae	Microbiome
26	Taxa_26	Firmicutes_Clostridia_Clostridiales_Clostridiaceae	Microbiome
27	Taxa_27	Firmicutes_Clostridia_Clostridiales_Eubacteriaceae	Microbiome
28	Taxa_28	Firmicutes_Clostridia_Clostridiales_Incertae Sedis XIII	Microbiome
29	Taxa_29	Firmicutes_Clostridia_Clostridiales_Incertae Sedis XIV	Microbiome
30	Taxa_30	Firmicutes_Clostridia_Clostridiales_Lachnospiraceae	Microbiome
31	Taxa_31	Firmicutes_Clostridia_Clostridiales_Peptostreptococcaceae	Microbiome
32	Taxa_32	Firmicutes_Clostridia_Clostridiales_Ruminococcaceae	Microbiome
33	Taxa_33	Firmicutes_Clostridia_Clostridiales_Veillonellaceae	Microbiome
34	Taxa_34	Firmicutes_Erysipelotrichi_Erysipelotrichales_Erysipelotrichaceae	Microbiome
35	Taxa_35	Fusobacteria_Fusobacteria_Fusobacteriales_Fusobacteriaceae	Microbiome
36	Taxa_36	Proteobacteria_Betaproteobacteria_Burkholderiales_Burkholderiales_incertae_sedis	Microbiome
37	Taxa_37	Proteobacteria_Deltaproteobacteria_Desulfovibrionales_Desulfovibrionaceae	Microbiome
38	Taxa_38	Proteobacteria_Gammaproteobacteria_Enterobacteriales_Enterobacteriaceae	Microbiome
39	LCU_39	LCU_0.74727875	LC_Urine
40	LCU_40	LCU_0.74927455	LC_Urine
41	LCU_41	LCU_0.75	LC_Urine
42	LCU_42	LCU_0.7500821	LC_Urine
43	LCU_43	LCU_0.75031143	LC_Urine
44	LCU_44	LCU_0.7506349	LC_Urine
45	LCU_45	LCU_0.75090474	LC_Urine
46	LCU_46	LCU_0.752541	LC_Urine
47	LCU_47	LCU_0.7537869	LC_Urine
48	LCU_48	LCU_0.75457627	LC_Urine
49	LCU_49	LCU_0.7553621	LC_Urine
50	LCU_50	LCU_0.755918	LC_Urine
51	LCU_51	LCU_0.75832564	LC_Urine
52	LCU_52	LCU_0.7631207	LC_Urine
53	LCU_53	LCU_0.77219296	LC_Urine
54	LCU_54	LCU_0.7892223	LC_Urine
55	LCU_55	LCU_0.7892727	LC_Urine
56	LCU_56	LCU_0.794889	LC_Urine
57	LCU_57	LCU_0.7954131	LC_Urine
58	LCU_58	LCU_0.79913557	LC_Urine
59	LCU_59	LCU_0.7999183	LC_Urine
60	LCU_60	LCU_0.8054529	LC_Urine
61	LCU_61	LCU_0.817	LC_Urine
62	LCU_62	LCU_0.82914275	LC_Urine
63	LCU_63	LCU_0.8293199	LC_Urine
64	LCU_64	LCU_0.8307142	LC_Urine
65	LCU_65	LCU_0.83115387	LC_Urine
66	LCU_66	LCU_0.83168626	LC_Urine
67	LCU_67	LCU_0.8319464	LC_Urine
68	LCU_68	LCU_0.8319555	LC_Urine
69	LCU_69	LCU_0.8323829	LC_Urine
70	LCU_70	LCU_0.8325651	LC_Urine
71	LCU_71	LCU_0.8326785	LC_Urine

72	LCU_72	LCU_0.8326862	LC_Urine
73	LCU_73	LCU_0.8327	LC_Urine
74	LCU_74	LCU_0.8328	LC_Urine
75	LCU_75	LCU_0.83282965	LC_Urine
76	LCU_76	LCU_0.8330195	LC_Urine
77	LCU_77	LCU_0.83307546	LC_Urine
78	LCU_78	LCU_0.833317	LC_Urine
79	LCU_79	LCU_0.8337407	LC_Urine
80	LCU_80	LCU_0.83375007	LC_Urine
81	LCU_81	LCU_0.83388	LC_Urine
82	LCU_82	LCU_0.8339	LC_Urine
83	LCU_83	LCU_0.8339583	LC_Urine
84	LCU_84	LCU_0.8343777	LC_Urine
85	LCU_85	LCU_0.8344	LC_Urine
86	LCU_86	LCU_0.8345966	LC_Urine
87	LCU_87	LCU_0.8347059	LC_Urine
88	LCU_88	LCU_0.8347273	LC_Urine
89	LCU_89	LCU_0.83483326	LC_Urine
90	LCU_90	LCU_0.83488	LC_Urine
91	LCU_91	LCU_0.8349272	LC_Urine
92	LCU_92	LCU_0.8349455	LC_Urine
93	LCU_93	LCU_0.8351154	LC_Urine
94	LCU_94	LCU_0.8352545	LC_Urine
95	LCU_95	LCU_0.8354468	LC_Urine
96	LCU_96	LCU_0.8355556	LC_Urine
97	LCU_97	LCU_0.8356512	LC_Urine
98	LCU_98	LCU_0.8359039	LC_Urine
99	LCU_99	LCU_0.83702224	LC_Urine
100	LCU_100	LCU_0.83703697	LC_Urine
101	LCU_101	LCU_0.8375193	LC_Urine
102	LCU_102	LCU_0.87418616	LC_Urine
103	LCU_103	LCU_0.8745832	LC_Urine
104	LCU_104	LCU_0.87646544	LC_Urine
105	LCU_105	LCU_0.8787965	LC_Urine
106	LCU_106	LCU_0.87987924	LC_Urine
107	LCU_107	LCU_0.8805	LC_Urine
108	LCU_108	LCU_0.8829792	LC_Urine
109	LCU_109	LCU_0.88521665	LC_Urine
110	LCU_110	LCU_0.8865102	LC_Urine
111	LCU_111	LCU_0.89766026	LC_Urine
112	LCU_112	LCU_0.9008372	LC_Urine
113	LCU_113	LCU_0.9008372	LC_Urine
114	LCU_114	LCU_0.9198982	LC_Urine
115	LCU_115	LCU_0.91999996	LC_Urine
116	LCU_116	LCU_0.9200508	LC_Urine
117	LCU_117	LCU_0.9251185	LC_Urine
118	LCU_118	LCU_0.9254614	LC_Urine
119	LCU_119	LCU_0.9260754	LC_Urine
120	LCU_120	LCU_0.926173	LC_Urine
121	LCU_121	LCU_0.9263409	LC_Urine
122	LCU_122	LCU_0.9269811	LC_Urine
123	LCU_123	LCU_0.9269999	LC_Urine
124	LCU_124	LCU_0.9306362	LC_Urine
125	LCU_125	LCU_0.93258536	LC_Urine
126	LCU_126	LCU_0.9426347	LC_Urine
127	LCU_127	LCU_0.9429642	LC_Urine
128	LCU_128	LCU_0.94850004	LC_Urine
129	LCU_129	LCU_0.9499769	LC_Urine
130	LCU_130	LCU_0.95090467	LC_Urine
131	LCU_131	LCU_0.9534468	LC_Urine
132	LCU_132	LCU_0.96072096	LC_Urine
133	LCU_133	LCU_0.9618981	LC_Urine

134	LCU_134	LCU_0.9632633	LC_Urine
135	LCU_135	LCU_0.9651641	LC_Urine
136	LCU_136	LCU_0.97273076	LC_Urine
137	LCU_137	LCU_0.985805	LC_Urine
138	LCU_138	LCU_0.9963092	LC_Urine
139	LCU_139	LCU_1.0073206	LC_Urine
140	LCU_140	LCU_1.015354	LC_Urine
141	LCU_141	LCU_1.0740485	LC_Urine
142	LCU_142	LCU_1.1610193	LC_Urine
143	LCU_143	LCU_1.2602726	LC_Urine
144	LCU_144	LCU_1.2623726	LC_Urine
145	LCU_145	LCU_1.267737	LC_Urine
146	LCU_146	LCU_1.2687	LC_Urine
147	LCU_147	LCU_1.2778	LC_Urine
148	LCU_148	LCU_1.2788914	LC_Urine
149	LCU_149	LCU_1.2788914	LC_Urine
150	LCU_150	LCU_1.279109	LC_Urine
151	LCU_151	LCU_1.280451	LC_Urine
152	LCU_152	LCU_1.2930889	LC_Urine
153	LCU_153	LCU_1.3214036	LC_Urine
154	LCU_154	LCU_1.3219131	LC_Urine
155	LCU_155	LCU_1.3337923	LC_Urine
156	LCU_156	LCU_1.3610377	LC_Urine
157	LCU_157	LCU_1.3614264	LC_Urine
158	LCU_158	LCU_1.3694761	LC_Urine
159	LCU_159	LCU_1.3711303	LC_Urine
160	LCU_160	LCU_1.3832453	LC_Urine
161	LCU_161	LCU_1.3952246	LC_Urine
162	LCU_162	LCU_1.4084749	LC_Urine
163	LCU_163	LCU_1.4326079	LC_Urine
164	LCU_164	LCU_1.4340222	LC_Urine
165	LCU_165	LCU_1.4741818	LC_Urine
166	LCU_166	LCU_1.4764813	LC_Urine
167	LCU_167	LCU_1.4820741	LC_Urine
168	LCU_168	LCU_1.4830698	LC_Urine
169	LCU_169	LCU_1.4837799	LC_Urine
170	LCU_170	LCU_1.4842181	LC_Urine
171	LCU_171	LCU_1.4860667	LC_Urine
172	LCU_172	LCU_1.487729	LC_Urine
173	LCU_173	LCU_1.4877455	LC_Urine
174	LCU_174	LCU_1.4883584	LC_Urine
175	LCU_175	LCU_1.490586	LC_Urine
176	LCU_176	LCU_1.4934629	LC_Urine
177	LCU_177	LCU_1.5206554	LC_Urine
178	LCU_178	LCU_1.5267242	LC_Urine
179	LCU_179	LCU_1.5351628	LC_Urine
180	LCU_180	LCU_1.5480783	LC_Urine
181	LCU_181	LCU_1.5799284	LC_Urine
182	LCU_182	LCU_1.5823072	LC_Urine
183	LCU_183	LCU_1.582964	LC_Urine
184	LCU_184	LCU_1.5838695	LC_Urine
185	LCU_185	LCU_1.5839664	LC_Urine
186	LCU_186	LCU_1.5841163	LC_Urine
187	LCU_187	LCU_1.5846099	LC_Urine
188	LCU_188	LCU_1.5855306	LC_Urine
189	LCU_189	LCU_1.5857404	LC_Urine
190	LCU_190	LCU_1.5861996	LC_Urine
191	LCU_191	LCU_1.5864478	LC_Urine
192	LCU_192	LCU_1.5866978	LC_Urine
193	LCU_193	LCU_1.5871603	LC_Urine
194	LCU_194	LCU_1.5890453	LC_Urine
195	LCU_195	LCU_1.5894725	LC_Urine

196	LCU_196	LCU_1.5896667	LC_Urine
197	LCU_197	LCU_1.5949998	LC_Urine
198	LCU_198	LCU_1.5974073	LC_Urine
199	LCU_199	LCU_1.6054822	LC_Urine
200	LCU_200	LCU_1.6100588	LC_Urine
201	LCU_201	LCU_1.6136792	LC_Urine
202	LCU_202	LCU_1.6173636	LC_Urine
203	LCU_203	LCU_1.6188293	LC_Urine
204	LCU_204	LCU_1.6190517	LC_Urine
205	LCU_205	LCU_1.6257869	LC_Urine
206	LCU_206	LCU_1.625981	LC_Urine
207	LCU_207	LCU_1.6272712	LC_Urine
208	LCU_208	LCU_1.6276001	LC_Urine
209	LCU_209	LCU_1.6280836	LC_Urine
210	LCU_210	LCU_1.6297739	LC_Urine
211	LCU_211	LCU_1.6349598	LC_Urine
212	LCU_212	LCU_1.6454388	LC_Urine
213	LCU_213	LCU_1.658018	LC_Urine
214	LCU_214	LCU_1.6587019	LC_Urine
215	LCU_215	LCU_1.6646327	LC_Urine
216	LCU_216	LCU_1.6919421	LC_Urine
217	LCU_217	LCU_1.7066609	LC_Urine
218	LCU_218	LCU_1.7088137	LC_Urine
219	LCU_219	LCU_1.7386442	LC_Urine
220	LCU_220	LCU_1.7901965	LC_Urine
221	LCU_221	LCU_1.811508	LC_Urine
222	LCU_222	LCU_1.8660964	LC_Urine
223	LCU_223	LCU_1.8672792	LC_Urine
224	LCU_224	LCU_1.8683732	LC_Urine
225	LCU_225	LCU_1.9070834	LC_Urine
226	LCU_226	LCU_1.9699509	LC_Urine
227	LCU_227	LCU_1.9724256	LC_Urine
228	LCU_228	LCU_1.9781467	LC_Urine
229	LCU_229	LCU_2.0142667	LC_Urine
230	LCU_230	LCU_2.031731	LC_Urine
231	LCU_231	LCU_2.033434	LC_Urine
232	LCU_232	LCU_2.035491	LC_Urine
233	LCU_233	LCU_2.1562004	LC_Urine
234	LCU_234	LCU_2.1580324	LC_Urine
235	LCU_235	LCU_2.1584098	LC_Urine
236	LCU_236	LCU_2.2056663	LC_Urine
237	LCU_237	LCU_2.2079535	LC_Urine
238	LCU_238	LCU_2.341478	LC_Urine
239	LCU_239	LCU_2.3936396	LC_Urine
240	LCU_240	LCU_2.39485	LC_Urine
241	LCU_241	LCU_2.3964212	LC_Urine
242	LCU_242	LCU_2.6387966	LC_Urine
243	LCU_243	LCU_2.6391184	LC_Urine
244	LCU_244	LCU_2.6424997	LC_Urine
245	LCU_245	LCU_2.645761	LC_Urine
246	LCU_246	LCU_2.6595964	LC_Urine
247	LCU_247	LCU_2.6604998	LC_Urine
248	LCU_248	LCU_2.7091873	LC_Urine
249	LCU_249	LCU_2.7255743	LC_Urine
250	LCU_250	LCU_2.7548604	LC_Urine
251	LCU_251	LCU_2.7589827	LC_Urine
252	LCU_252	LCU_2.7684414	LC_Urine
253	LCU_253	LCU_2.802426	LC_Urine
254	LCU_254	LCU_2.8698545	LC_Urine
255	LCU_255	LCU_2.9481251	LC_Urine
256	LCU_256	LCU_3.0346272	LC_Urine
257	LCU_257	LCU_3.0549347	LC_Urine

258	LCU_258	LCU_3.1949997	LC_Urine
259	LCU_259	LCU_3.2131965	LC_Urine
260	LCU_260	LCU_3.299293	LC_Urine
261	LCU_261	LCU_3.5435	LC_Urine
262	LCU_262	LCU_3.7233748	LC_Urine
263	LCU_263	LCU_4.438754	LC_Urine
264	LCU_264	LCU_4.4903407	LC_Urine
265	LCU_265	LCU_4.6014996	LC_Urine
266	LCU_266	LCU_4.6609545	LC_Urine
267	LCU_267	LCU_4.7638125	LC_Urine
268	LCU_268	LCU_4.765773	LC_Urine
269	LCU_269	LCU_4.765911	LC_Urine
270	LCU_270	LCU_4.766158	LC_Urine
271	LCU_271	LCU_4.7718625	LC_Urine
272	LCU_272	LCU_4.7718625	LC_Urine
273	LCU_273	LCU_4.8621707	LC_Urine
274	LCU_274	LCU_4.908667	LC_Urine
275	LCU_275	LCU_4.9140444	LC_Urine
276	LCU_276	LCU_4.916358	LC_Urine
277	LCU_277	LCU_4.9432173	LC_Urine
278	LCU_278	LCU_4.94539	LC_Urine
279	LCU_279	LCU_4.9456315	LC_Urine
280	LCU_280	LCU_4.9519105	LC_Urine
281	LCU_281	LCU_5.152652	LC_Urine
282	LCU_282	LCU_5.154371	LC_Urine
283	LCU_283	LCU_5.154371	LC_Urine
284	LCU_284	LCU_5.1904693	LC_Urine
285	LCU_285	LCU_5.1935616	LC_Urine
286	LCU_286	LCU_5.1938496	LC_Urine
287	LCU_287	LCU_5.1945767	LC_Urine
288	LCU_288	LCU_5.1948285	LC_Urine
289	LCU_289	LCU_5.199934	LC_Urine
290	LCU_290	LCU_5.2008705	LC_Urine
291	LCU_291	LCU_5.2108912	LC_Urine
292	LCU_292	LCU_5.2199836	LC_Urine
293	LCU_293	LCU_5.2523804	LC_Urine
294	LCU_294	LCU_5.26583	LC_Urine
295	LCU_295	LCU_5.3574414	LC_Urine
296	LCU_296	LCU_5.4184885	LC_Urine
297	LCU_297	LCU_5.4312587	LC_Urine
298	LCU_298	LCU_5.4375343	LC_Urine
299	LCU_299	LCU_5.4512296	LC_Urine
300	LCU_300	LCU_5.4528127	LC_Urine
301	LCU_301	LCU_5.481099	LC_Urine
302	LCU_302	LCU_5.483327	LC_Urine
303	LCU_303	LCU_5.485268	LC_Urine
304	LCU_304	LCU_5.5079618	LC_Urine
305	LCU_305	LCU_5.508163	LC_Urine
306	LCU_306	LCU_5.5625243	LC_Urine
307	LCU_307	LCU_5.5652394	LC_Urine
308	LCU_308	LCU_5.568741	LC_Urine
309	LCU_309	LCU_5.569786	LC_Urine
310	LCU_310	LCU_5.611671	LC_Urine
311	LCU_311	LCU_5.6127157	LC_Urine
312	LCU_312	LCU_5.6135116	LC_Urine
313	LCU_313	LCU_5.6304255	LC_Urine
314	LCU_314	LCU_5.6408687	LC_Urine
315	LCU_315	LCU_5.640949	LC_Urine
316	LCU_316	LCU_5.6759615	LC_Urine
317	LCU_317	LCU_5.7831793	LC_Urine
318	LCU_318	LCU_5.7832956	LC_Urine
319	LCU_319	LCU_5.78345	LC_Urine

320	LCU_320	LCU_5.7836595	LC_Urine
321	LCU_321	LCU_5.7961636	LC_Urine
322	LCU_322	LCU_5.797979	LC_Urine
323	LCU_323	LCU_5.8026514	LC_Urine
324	LCU_324	LCU_5.861306	LC_Urine
325	LCU_325	LCU_5.9078	LC_Urine
326	LCU_326	LCU_5.9280505	LC_Urine
327	LCU_327	LCU_5.98149	LC_Urine
328	LCU_328	LCU_6.0272217	LC_Urine
329	LCU_329	LCU_6.062381	LC_Urine
330	LCU_330	LCU_6.070278	LC_Urine
331	LCU_331	LCU_6.07372	LC_Urine
332	LCU_332	LCU_6.077568	LC_Urine
333	LCU_333	LCU_6.086763	LC_Urine
334	LCU_334	LCU_6.09863	LC_Urine
335	LCU_335	LCU_6.1219807	LC_Urine
336	LCU_336	LCU_6.122539	LC_Urine
337	LCU_337	LCU_6.1241083	LC_Urine
338	LCU_338	LCU_6.1274805	LC_Urine
339	LCU_339	LCU_6.136942	LC_Urine
340	LCU_340	LCU_6.1466746	LC_Urine
341	LCU_341	LCU_6.213277	LC_Urine
342	LCU_342	LCU_6.286322	LC_Urine
343	LCU_343	LCU_6.352967	LC_Urine
344	LCU_344	LCU_6.3543324	LC_Urine
345	LCU_345	LCU_6.4223166	LC_Urine
346	LCU_346	LCU_6.4357796	LC_Urine
347	LCU_347	LCU_6.4629674	LC_Urine
348	LCU_348	LCU_6.4682045	LC_Urine
349	LCU_349	LCU_6.5068154	LC_Urine
350	LCU_350	LCU_6.6587186	LC_Urine
351	LCU_351	LCU_6.681644	LC_Urine
352	LCU_352	LCU_6.7164106	LC_Urine
353	LCU_353	LCU_6.7721176	LC_Urine
354	LCU_354	LCU_6.7893596	LC_Urine
355	LCU_355	LCU_6.7908297	LC_Urine
356	LCU_356	LCU_6.805694	LC_Urine
357	LCU_357	LCU_6.8114405	LC_Urine
358	LCU_358	LCU_6.8166566	LC_Urine
359	LCU_359	LCU_6.819189	LC_Urine
360	LCU_360	LCU_6.853128	LC_Urine
361	LCU_361	LCU_6.870501	LC_Urine
362	LCU_362	LCU_6.8725004	LC_Urine
363	LCU_363	LCU_6.8748856	LC_Urine
364	LCU_364	LCU_6.8752184	LC_Urine
365	LCU_365	LCU_6.8756604	LC_Urine
366	LCU_366	LCU_6.876167	LC_Urine
367	LCU_367	LCU_6.876308	LC_Urine
368	LCU_368	LCU_6.876892	LC_Urine
369	LCU_369	LCU_6.8770485	LC_Urine
370	LCU_370	LCU_6.877236	LC_Urine
371	LCU_371	LCU_6.877356	LC_Urine
372	LCU_372	LCU_6.87739	LC_Urine
373	LCU_373	LCU_6.8775887	LC_Urine
374	LCU_374	LCU_6.8777957	LC_Urine
375	LCU_375	LCU_6.877911	LC_Urine
376	LCU_376	LCU_6.8780837	LC_Urine
377	LCU_377	LCU_6.878209	LC_Urine
378	LCU_378	LCU_6.8783264	LC_Urine
379	LCU_379	LCU_6.8790364	LC_Urine
380	LCU_380	LCU_6.879132	LC_Urine
381	LCU_381	LCU_6.8791623	LC_Urine

382	LCU_382	LCU_6.880388	LC_Urine
383	LCU_383	LCU_6.8810234	LC_Urine
384	LCU_384	LCU_6.8816586	LC_Urine
385	LCU_385	LCU_6.882289	LC_Urine
386	LCU_386	LCU_6.8835697	LC_Urine
387	LCU_387	LCU_6.9340515	LC_Urine
388	LCU_388	LCU_6.9370008	LC_Urine
389	LCU_389	LCU_6.9504914	LC_Urine
390	LCU_390	LCU_6.9522223	LC_Urine
391	LCU_391	LCU_6.9522963	LC_Urine
392	LCU_392	LCU_6.954574	LC_Urine
393	LCU_393	LCU_6.9557014	LC_Urine
394	LCU_394	LCU_6.9559526	LC_Urine
395	LCU_395	LCU_6.9560647	LC_Urine
396	LCU_396	LCU_6.956492	LC_Urine
397	LCU_397	LCU_6.957068	LC_Urine
398	LCU_398	LCU_6.957544	LC_Urine
399	LCU_399	LCU_6.958148	LC_Urine
400	LCU_400	LCU_6.9584837	LC_Urine
401	LCU_401	LCU_6.9586844	LC_Urine
402	LCU_402	LCU_6.9588256	LC_Urine
403	LCU_403	LCU_6.959577	LC_Urine
404	LCU_404	LCU_6.961377	LC_Urine
405	LCU_405	LCU_6.962525	LC_Urine
406	LCU_406	LCU_6.965726	LC_Urine
407	LCU_407	LCU_6.969448	LC_Urine
408	LCU_408	LCU_6.9710565	LC_Urine
409	LCU_409	LCU_6.972352	LC_Urine
410	LCU_410	LCU_6.9731226	LC_Urine
411	LCU_411	LCU_6.973138	LC_Urine
412	LCU_412	LCU_6.9741073	LC_Urine
413	LCU_413	LCU_6.976765	LC_Urine
414	LCU_414	LCU_6.978379	LC_Urine
415	LCU_415	LCU_6.9786825	LC_Urine
416	LCU_416	LCU_7.013635	LC_Urine
417	LCU_417	LCU_7.0158486	LC_Urine
418	LCU_418	LCU_7.0160627	LC_Urine
419	LCU_419	LCU_7.037093	LC_Urine
420	LCU_420	LCU_7.037522	LC_Urine
421	LCU_421	LCU_7.040122	LC_Urine
422	LCU_422	LCU_7.0494294	LC_Urine
423	LCU_423	LCU_7.0498953	LC_Urine
424	LCU_424	LCU_7.071801	LC_Urine
425	LCU_425	LCU_7.0998015	LC_Urine
426	LCU_426	LCU_7.100242	LC_Urine
427	LCU_427	LCU_7.100775	LC_Urine
428	LCU_428	LCU_7.1207232	LC_Urine
429	LCU_429	LCU_7.1270194	LC_Urine
430	LCU_430	LCU_7.211143	LC_Urine
431	LCU_431	LCU_7.21354	LC_Urine
432	LCU_432	LCU_7.244039	LC_Urine
433	LCU_433	LCU_7.244427	LC_Urine
434	LCU_434	LCU_7.3495092	LC_Urine
435	LCU_435	LCU_7.3521667	LC_Urine
436	LCU_436	LCU_7.352296	LC_Urine
437	LCU_437	LCU_7.365115	LC_Urine
438	LCU_438	LCU_7.4260163	LC_Urine
439	LCU_439	LCU_7.434476	LC_Urine
440	LCU_440	LCU_7.4347143	LC_Urine
441	LCU_441	LCU_7.491035	LC_Urine
442	LCU_442	LCU_7.499751	LC_Urine
443	LCU_443	LCU_7.542869	LC_Urine

444	LCU_444	LCU_7.5485697	LC_Urine
445	LCU_445	LCU_7.5485697	LC_Urine
446	LCU_446	LCU_7.555021	LC_Urine
447	LCU_447	LCU_7.5700464	LC_Urine
448	LCU_448	LCU_7.5703554	LC_Urine
449	LCU_449	LCU_7.575164	LC_Urine
450	LCU_450	LCU_7.57579	LC_Urine
451	LCU_451	LCU_7.6323013	LC_Urine
452	LCU_452	LCU_7.6323013	LC_Urine
453	LCU_453	LCU_7.6389775	LC_Urine
454	LCU_454	LCU_7.725359	LC_Urine
455	LCU_455	LCU_7.7751837	LC_Urine
456	LCU_456	LCU_7.77587	LC_Urine
457	LCU_457	LCU_7.8139033	LC_Urine
458	LCU_458	LCU_7.8139033	LC_Urine
459	LCU_459	LCU_7.8355966	LC_Urine
460	LCU_460	LCU_7.8360004	LC_Urine
461	LCU_461	LCU_7.8384027	LC_Urine
462	LCU_462	LCU_7.912367	LC_Urine
463	LCU_463	LCU_7.9271274	LC_Urine
464	LCU_464	LCU_7.9561768	LC_Urine
465	LCU_465	LCU_7.9772396	LC_Urine
466	LCU_466	LCU_7.989882	LC_Urine
467	LCU_467	LCU_8.005605	LC_Urine
468	LCU_468	LCU_8.005952	LC_Urine
469	LCU_469	LCU_8.007562	LC_Urine
470	LCU_470	LCU_8.026418	LC_Urine
471	LCU_471	LCU_8.117298	LC_Urine
472	LCU_472	LCU_8.121244	LC_Urine
473	LCU_473	LCU_8.130238	LC_Urine
474	LCU_474	LCU_8.162313	LC_Urine
475	LCU_475	LCU_8.255415	LC_Urine
476	LCU_476	LCU_8.2583	LC_Urine
477	LCU_477	LCU_8.271348	LC_Urine
478	LCU_478	LCU_8.379254	LC_Urine
479	LCU_479	LCU_8.400715	LC_Urine
480	LCU_480	LCU_8.449583	LC_Urine
481	LCU_481	LCU_8.452721	LC_Urine
482	LCU_482	LCU_8.522912	LC_Urine
483	LCU_483	LCU_8.5915365	LC_Urine
484	LCU_484	LCU_8.619595	LC_Urine
485	LCU_485	LCU_8.698693	LC_Urine
486	LCU_486	LCU_8.710734	LC_Urine
487	LCU_487	LCU_8.722143	LC_Urine
488	LCU_488	LCU_8.722477	LC_Urine
489	LCU_489	LCU_8.722596	LC_Urine
490	LCU_490	LCU_8.738303	LC_Urine
491	LCU_491	LCU_8.770103	LC_Urine
492	LCU_492	LCU_8.816201	LC_Urine
493	LCU_493	LCU_8.816201	LC_Urine
494	LCU_494	LCU_8.839639	LC_Urine
495	LCU_495	LCU_8.840083	LC_Urine
496	LCU_496	LCU_8.847407	LC_Urine
497	LCU_497	LCU_8.885116	LC_Urine
498	LCU_498	LCU_8.907862	LC_Urine
499	LCU_499	LCU_8.908082	LC_Urine
500	LCU_500	LCU_8.938262	LC_Urine
501	LCU_501	LCU_8.945626	LC_Urine
502	LCU_502	LCU_8.972689	LC_Urine
503	LCU_503	LCU_8.973355	LC_Urine
504	LCU_504	LCU_8.9947	LC_Urine
505	LCU_505	LCU_8.9947	LC_Urine

506	LCU_506	LCU_8.997327	LC_Urine
507	LCU_507	LCU_9.024211	LC_Urine
508	LCU_508	LCU_9.074976	LC_Urine
509	LCU_509	LCU_9.101583	LC_Urine
510	LCU_510	LCU_9.139768	LC_Urine
511	LCU_511	LCU_9.140408	LC_Urine
512	LCU_512	LCU_9.173713	LC_Urine
513	LCU_513	LCU_9.2345085	LC_Urine
514	LCU_514	LCU_9.236141	LC_Urine
515	LCU_515	LCU_9.371733	LC_Urine
516	LCU_516	LCU_9.379313	LC_Urine
517	LCU_517	LCU_9.464517	LC_Urine
518	LCU_518	LCU_9.46946	LC_Urine
519	LCU_519	LCU_9.470442	LC_Urine
520	LCU_520	LCU_9.470485	LC_Urine
521	LCU_521	LCU_9.50072	LC_Urine
522	LCU_522	LCU_9.561614	LC_Urine
523	LCU_523	LCU_9.578851	LC_Urine
524	LCU_524	LCU_9.645567	LC_Urine
525	LCU_525	LCU_9.720254	LC_Urine
526	LCU_526	LCU_9.751905	LC_Urine
527	LCU_527	LCU_9.773757	LC_Urine
528	LCU_528	LCU_9.774888	LC_Urine
529	LCU_529	LCU_9.788501	LC_Urine
530	LCU_530	LCU_9.994791	LC_Urine
531	LCU_531	LCU_10.031607	LC_Urine
532	LCU_532	LCU_10.077939	LC_Urine
533	LCU_533	LCU_10.079326	LC_Urine
534	LCU_534	LCU_10.080652	LC_Urine
535	LCU_535	LCU_10.0996475	LC_Urine
536	LCU_536	LCU_10.102722	LC_Urine
537	LCU_537	LCU_10.105436	LC_Urine
538	LCU_538	LCU_10.212898	LC_Urine
539	LCU_539	LCU_10.309588	LC_Urine
540	LCU_540	LCU_10.353525	LC_Urine
541	LCU_541	LCU_10.355059	LC_Urine
542	LCU_542	LCU_10.432632	LC_Urine
543	LCU_543	LCU_10.43348	LC_Urine
544	LCU_544	LCU_10.503603	LC_Urine
545	LCU_545	LCU_10.653122	LC_Urine
546	LCU_546	LCU_10.775558	LC_Urine
547	LCU_547	LCU_10.8821535	LC_Urine
548	LCU_548	LCU_10.928152	LC_Urine
549	LCU_549	LCU_10.947792	LC_Urine
550	LCU_550	LCU_11.206437	LC_Urine
551	LCU_551	LCU_11.276952	LC_Urine
552	LCU_552	LCU_11.3586645	LC_Urine
553	LCU_553	LCU_11.456981	LC_Urine
554	LCU_554	LCU_11.459462	LC_Urine
555	LCU_555	LCU_11.544444	LC_Urine
556	LCU_556	LCU_11.546586	LC_Urine
557	LCU_557	LCU_11.546889	LC_Urine
558	LCU_558	LCU_11.54727	LC_Urine
559	LCU_559	LCU_11.613403	LC_Urine
560	LCU_560	LCU_11.637125	LC_Urine
561	LCU_561	LCU_11.6875	LC_Urine
562	LCU_562	LCU_11.775129	LC_Urine
563	LCU_563	LCU_11.979732	LC_Urine
564	LCU_564	LCU_12.112909	LC_Urine
565	LCU_565	LCU_12.175001	LC_Urine
566	LCU_566	LCU_12.199838	LC_Urine
567	LCU_567	LCU_12.262382	LC_Urine

568	LCU_568	LCU_12.28367	LC_Urine
569	LCU_569	LCU_12.318223	LC_Urine
570	LCU_570	LCU_12.345813	LC_Urine
571	LCU_571	LCU_12.39081	LC_Urine
572	LCU_572	LCU_12.574298	LC_Urine
573	LCU_573	LCU_12.622506	LC_Urine
574	LCU_574	LCU_12.683618	LC_Urine
575	LCU_575	LCU_12.687773	LC_Urine
576	LCU_576	LCU_12.708569	LC_Urine
577	LCU_577	LCU_12.731935	LC_Urine
578	LCU_578	LCU_12.735333	LC_Urine
579	LCU_579	LCU_12.816047	LC_Urine
580	LCU_580	LCU_12.819522	LC_Urine
581	LCU_581	LCU_12.831981	LC_Urine
582	LCU_582	LCU_12.885777	LC_Urine
583	LCU_583	LCU_12.932332	LC_Urine
584	LCU_584	LCU_12.990493	LC_Urine
585	LCU_585	LCU_13.011092	LC_Urine
586	LCU_586	LCU_13.047636	LC_Urine
587	LCU_587	LCU_13.050343	LC_Urine
588	LCU_588	LCU_13.165144	LC_Urine
589	LCU_589	LCU_13.166287	LC_Urine
590	LCU_590	LCU_13.212046	LC_Urine
591	LCU_591	LCU_13.268795	LC_Urine
592	LCU_592	LCU_13.288082	LC_Urine
593	LCU_593	LCU_13.2883215	LC_Urine
594	LCU_594	LCU_13.364589	LC_Urine
595	LCU_595	LCU_13.465035	LC_Urine
596	LCU_596	LCU_13.520692	LC_Urine
597	LCU_597	LCU_13.632856	LC_Urine
598	LCU_598	LCU_13.647049	LC_Urine
599	LCU_599	LCU_13.874223	LC_Urine
600	LCU_600	LCU_13.942835	LC_Urine
601	LCU_601	LCU_14.094934	LC_Urine
602	LCU_602	LCU_14.145176	LC_Urine
603	LCU_603	LCU_14.145303	LC_Urine
604	LCU_604	LCU_14.251763	LC_Urine
605	LCU_605	LCU_14.266001	LC_Urine
606	LCU_606	LCU_14.281033	LC_Urine
607	LCU_607	LCU_14.298923	LC_Urine
608	LCU_608	LCU_14.304633	LC_Urine
609	LCU_609	LCU_14.35792	LC_Urine
610	LCU_610	LCU_14.358581	LC_Urine
611	LCU_611	LCU_14.359205	LC_Urine
612	LCU_612	LCU_14.361777	LC_Urine
613	LCU_613	LCU_14.361777	LC_Urine
614	LCU_614	LCU_14.365107	LC_Urine
615	LCU_615	LCU_14.365107	LC_Urine
616	LCU_616	LCU_14.453684	LC_Urine
617	LCU_617	LCU_14.461346	LC_Urine
618	LCU_618	LCU_14.5082655	LC_Urine
619	LCU_619	LCU_14.513499	LC_Urine
620	LCU_620	LCU_14.563794	LC_Urine
621	LCU_621	LCU_14.722332	LC_Urine
622	LCU_622	LCU_14.737936	LC_Urine
623	LCU_623	LCU_14.74322	LC_Urine
624	LCU_624	LCU_14.795255	LC_Urine
625	LCU_625	LCU_14.850871	LC_Urine
626	LCU_626	LCU_14.855693	LC_Urine
627	LCU_627	LCU_14.894733	LC_Urine
628	LCU_628	LCU_14.981606	LC_Urine
629	LCU_629	LCU_14.983619	LC_Urine

630	LCU_630	LCU_14.98419	LC_Urine
631	LCU_631	LCU_14.98419	LC_Urine
632	LCU_632	LCU_14.98438	LC_Urine
633	LCU_633	LCU_15.080557	LC_Urine
634	LCU_634	LCU_15.086254	LC_Urine
635	LCU_635	LCU_15.14852	LC_Urine
636	LCU_636	LCU_15.151192	LC_Urine
637	LCU_637	LCU_15.205523	LC_Urine
638	LCU_638	LCU_15.225825	LC_Urine
639	LCU_639	LCU_15.315269	LC_Urine
640	LCU_640	LCU_15.422077	LC_Urine
641	LCU_641	LCU_15.437079	LC_Urine
642	LCU_642	LCU_15.469015	LC_Urine
643	LCU_643	LCU_15.495161	LC_Urine
644	LCU_644	LCU_15.588093	LC_Urine
645	LCU_645	LCU_15.67758	LC_Urine
646	LCU_646	LCU_15.6806	LC_Urine
647	LCU_647	LCU_15.680611	LC_Urine
648	LCU_648	LCU_15.681091	LC_Urine
649	LCU_649	LCU_15.681164	LC_Urine
650	LCU_650	LCU_15.68129	LC_Urine
651	LCU_651	LCU_15.681965	LC_Urine
652	LCU_652	LCU_15.68209	LC_Urine
653	LCU_653	LCU_15.683433	LC_Urine
654	LCU_654	LCU_15.68474	LC_Urine
655	LCU_655	LCU_15.710089	LC_Urine
656	LCU_656	LCU_15.713095	LC_Urine
657	LCU_657	LCU_15.74408	LC_Urine
658	LCU_658	LCU_15.759273	LC_Urine
659	LCU_659	LCU_15.762667	LC_Urine
660	LCU_660	LCU_15.768744	LC_Urine
661	LCU_661	LCU_15.778476	LC_Urine
662	LCU_662	LCU_15.7982855	LC_Urine
663	LCU_663	LCU_15.813449	LC_Urine
664	LCU_664	LCU_15.896331	LC_Urine
665	LCU_665	LCU_15.900883	LC_Urine
666	LCU_666	LCU_15.906646	LC_Urine
667	LCU_667	LCU_15.912666	LC_Urine
668	LCU_668	LCU_15.916633	LC_Urine
669	LCU_669	LCU_15.946351	LC_Urine
670	LCU_670	LCU_15.960944	LC_Urine
671	LCU_671	LCU_16.002102	LC_Urine
672	LCU_672	LCU_16.004103	LC_Urine
673	LCU_673	LCU_16.005022	LC_Urine
674	LCU_674	LCU_16.010103	LC_Urine
675	LCU_675	LCU_16.01177	LC_Urine
676	LCU_676	LCU_16.013063	LC_Urine
677	LCU_677	LCU_16.01392	LC_Urine
678	LCU_678	LCU_16.053082	LC_Urine
679	LCU_679	LCU_16.085033	LC_Urine
680	LCU_680	LCU_16.104239	LC_Urine
681	LCU_681	LCU_16.113829	LC_Urine
682	LCU_682	LCU_16.139267	LC_Urine
683	LCU_683	LCU_16.148588	LC_Urine
684	LCU_684	LCU_16.155415	LC_Urine
685	LCU_685	LCU_16.15587	LC_Urine
686	LCU_686	LCU_16.156857	LC_Urine
687	LCU_687	LCU_16.160295	LC_Urine
688	LCU_688	LCU_16.164032	LC_Urine
689	LCU_689	LCU_16.167204	LC_Urine
690	LCU_690	LCU_16.19138	LC_Urine
691	LCU_691	LCU_16.205095	LC_Urine

692	LCU_692	LCU_16.231874	LC_Urine
693	LCU_693	LCU_16.254398	LC_Urine
694	LCU_694	LCU_16.297667	LC_Urine
695	LCU_695	LCU_16.301191	LC_Urine
696	LCU_696	LCU_16.304703	LC_Urine
697	LCU_697	LCU_16.30576	LC_Urine
698	LCU_698	LCU_16.315142	LC_Urine
699	LCU_699	LCU_16.327337	LC_Urine
700	LCU_700	LCU_16.358772	LC_Urine
701	LCU_701	LCU_16.374523	LC_Urine
702	LCU_702	LCU_16.384253	LC_Urine
703	LCU_703	LCU_16.412611	LC_Urine
704	LCU_704	LCU_16.424555	LC_Urine
705	LCU_705	LCU_16.443018	LC_Urine
706	LCU_706	LCU_16.44573	LC_Urine
707	LCU_707	LCU_16.447248	LC_Urine
708	LCU_708	LCU_16.45167	LC_Urine
709	LCU_709	LCU_16.452784	LC_Urine
710	LCU_710	LCU_16.453796	LC_Urine
711	LCU_711	LCU_16.453957	LC_Urine
712	LCU_712	LCU_16.49991	LC_Urine
713	LCU_713	LCU_16.530794	LC_Urine
714	LCU_714	LCU_16.531557	LC_Urine
715	LCU_715	LCU_16.531761	LC_Urine
716	LCU_716	LCU_16.563206	LC_Urine
717	LCU_717	LCU_16.592648	LC_Urine
718	LCU_718	LCU_16.594654	LC_Urine
719	LCU_719	LCU_16.630138	LC_Urine
720	LCU_720	LCU_16.651237	LC_Urine
721	LCU_721	LCU_16.657778	LC_Urine
722	LCU_722	LCU_16.690538	LC_Urine
723	LCU_723	LCU_16.72175	LC_Urine
724	LCU_724	LCU_16.733065	LC_Urine
725	LCU_725	LCU_16.740698	LC_Urine
726	LCU_726	LCU_16.750484	LC_Urine
727	LCU_727	LCU_16.76293	LC_Urine
728	LCU_728	LCU_16.76293	LC_Urine
729	LCU_729	LCU_16.76756	LC_Urine
730	LCU_730	LCU_16.797155	LC_Urine
731	LCU_731	LCU_16.797253	LC_Urine
732	LCU_732	LCU_16.803474	LC_Urine
733	LCU_733	LCU_16.840853	LC_Urine
734	LCU_734	LCU_16.85239	LC_Urine
735	LCU_735	LCU_16.86802	LC_Urine
736	LCU_736	LCU_16.889019	LC_Urine
737	LCU_737	LCU_16.901873	LC_Urine
738	LCU_738	LCU_16.904327	LC_Urine
739	LCU_739	LCU_16.906145	LC_Urine
740	LCU_740	LCU_16.913258	LC_Urine
741	LCU_741	LCU_16.926037	LC_Urine
742	LCU_742	LCU_16.927883	LC_Urine
743	LCU_743	LCU_16.942999	LC_Urine
744	LCU_744	LCU_16.947811	LC_Urine
745	LCU_745	LCU_16.9505	LC_Urine
746	LCU_746	LCU_16.95604	LC_Urine
747	LCU_747	LCU_16.957428	LC_Urine
748	LCU_748	LCU_16.95881	LC_Urine
749	LCU_749	LCU_16.959627	LC_Urine
750	LCU_750	LCU_16.965256	LC_Urine
751	LCU_751	LCU_16.96902	LC_Urine
752	LCU_752	LCU_16.986	LC_Urine
753	LCU_753	LCU_16.987621	LC_Urine

754	LCU_754	LCU_16.991556	LC_Urine
755	LCU_755	LCU_16.999695	LC_Urine
756	LCU_756	LCU_17.001925	LC_Urine
757	LCU_757	LCU_17.010366	LC_Urine
758	LCU_758	LCU_17.023525	LC_Urine
759	LCU_759	LCU_17.02505	LC_Urine
760	LCU_760	LCU_17.028112	LC_Urine
761	LCU_761	LCU_17.051466	LC_Urine
762	LCU_762	LCU_17.054537	LC_Urine
763	LCU_763	LCU_17.059132	LC_Urine
764	LCU_764	LCU_17.074694	LC_Urine
765	LCU_765	LCU_17.092299	LC_Urine
766	LCU_766	LCU_17.101738	LC_Urine
767	LCU_767	LCU_17.11657	LC_Urine
768	LCU_768	LCU_17.125387	LC_Urine
769	LCU_769	LCU_17.125635	LC_Urine
770	LCU_770	LCU_17.160244	LC_Urine
771	LCU_771	LCU_17.160688	LC_Urine
772	LCU_772	LCU_17.162664	LC_Urine
773	LCU_773	LCU_17.164072	LC_Urine
774	LCU_774	LCU_17.166054	LC_Urine
775	LCU_775	LCU_17.166166	LC_Urine
776	LCU_776	LCU_17.166704	LC_Urine
777	LCU_777	LCU_17.166906	LC_Urine
778	LCU_778	LCU_17.166945	LC_Urine
779	LCU_779	LCU_17.16811	LC_Urine
780	LCU_780	LCU_17.169128	LC_Urine
781	LCU_781	LCU_17.170296	LC_Urine
782	LCU_782	LCU_17.175451	LC_Urine
783	LCU_783	LCU_17.178919	LC_Urine
784	LCU_784	LCU_17.182117	LC_Urine
785	LCU_785	LCU_17.186037	LC_Urine
786	LCU_786	LCU_17.186518	LC_Urine
787	LCU_787	LCU_17.186779	LC_Urine
788	LCU_788	LCU_17.198364	LC_Urine
789	LCU_789	LCU_17.203732	LC_Urine
790	LCU_790	LCU_17.224146	LC_Urine
791	LCU_791	LCU_17.287683	LC_Urine
792	LCU_792	LCU_17.288176	LC_Urine
793	LCU_793	LCU_17.29038	LC_Urine
794	LCU_794	LCU_17.29657	LC_Urine
795	LCU_795	LCU_17.320658	LC_Urine
796	LCS_796	LCS_glychocholic acid	LC_Serum
797	LCS_797	LCS_1-heptadecanoyl-sn-glycero-3-phosphocholine	LC_Serum
798	LCS_798	LCS_1-O-Hexadecyl-2-O-arachidonoyl-sn-glycero-3-phosphorylcholine	LC_Serum
799	LCS_799	LCS_2-Methylbutyroylcarnitine	LC_Serum
800	LCS_800	LCS_3-O-Hexadecyl-2-O-acetyl-sn-glycero-1-phosphorylcholine	LC_Serum
801	LCS_801	LCS_Acetylcarnitine	LC_Serum
802	LCS_802	LCS_AcetylCarnitineC3:0	LC_Serum
803	LCS_803	LCS_Acylcarnitine C14:0	LC_Serum
804	LCS_804	LCS_Acylcarnitine C14:1	LC_Serum
805	LCS_805	LCS_Acylcarnitine C5:0	LC_Serum
806	LCS_806	LCS_Acylcarnitine C6:0	LC_Serum
807	LCS_807	LCS_Betaine	LC_Serum
808	LCS_808	LCS_Butyryl-L-carnitine	LC_Serum
809	LCS_809	LCS_Carnitine	LC_Serum
810	LCS_810	LCS_Choline	LC_Serum
811	LCS_811	LCS_Creatinine	LC_Serum
812	LCS_812	LCS_Decanoyl-L-carnitine	LC_Serum
813	LCS_813	LCS_Dioctyl phthalate Contaminent	LC_Serum
814	LCS_814	LCS_DL-Stearoylcarnitine	LC_Serum
815	LCS_815	LCS_Elaidiccarnitine	LC_Serum

816	LCS_816	LCS_Inosine	LC_Serum
817	LCS_817	LCS_L-Octanoylcarnitine	LC_Serum
818	LCS_818	LCS_PC 38:5	LC_Serum
819	LCS_819	LCS_PC16:0	LC_Serum
820	LCS_820	LCS_PC18:2	LC_Serum
821	LCS_821	LCS_PC32:1	LC_Serum
822	LCS_822	LCS_PC34:2	LC_Serum
823	LCS_823	LCS_PC36:4	LC_Serum
824	LCS_824	LCS_PC36:5	LC_Serum
825	LCS_825	LCS_PC38:4	LC_Serum
826	LCS_826	LCS_PC38:5	LC_Serum
827	LCS_827	LCS_PC38:6	LC_Serum
828	LCS_828	LCS_PC40:5	LC_Serum
829	LCS_829	LCS_PC40:6	LC_Serum
830	LCS_830	LCS_PELLETIERINE	LC_Serum
831	LCS_831	LCS_Phe Phe	LC_Serum
832	LCS_832	LCS_Phenylalanine	LC_Serum
833	LCS_833	LCS_Piperine	LC_Serum
834	LCS_834	LCS_Proline	LC_Serum
835	LCS_835	LCS_Propionyl-L-carnitine	LC_Serum
836	LCS_836	LCS_Theophylline	LC_Serum
837	LCS_837	LCS_Valine	LC_Serum
838	GCS_838	GCS_xanthine	GC_Serum
839	GCS_839	GCS_valine	GC_Serum
840	GCS_840	GCS_uridine	GC_Serum
841	GCS_841	GCS_uric acid	GC_Serum
842	GCS_842	GCS_urea	GC_Serum
843	GCS_843	GCS_tyrosine	GC_Serum
844	GCS_844	GCS_tryptophan	GC_Serum
845	GCS_845	GCS_trans-4-hydroxyproline	GC_Serum
846	GCS_846	GCS_tocopherol alpha	GC_Serum
847	GCS_847	GCS_thymine	GC_Serum
848	GCS_848	GCS_threose	GC_Serum
849	GCS_849	GCS_threonine	GC_Serum
850	GCS_850	GCS_threonic acid	GC_Serum
851	GCS_851	GCS_taurine	GC_Serum
852	GCS_852	GCS_succinic acid	GC_Serum
853	GCS_853	GCS_stearic acid	GC_Serum
854	GCS_854	GCS_sorbitol	GC_Serum
855	GCS_855	GCS_shikimic acid	GC_Serum
856	GCS_856	GCS_serine	GC_Serum
857	GCS_857	GCS_salicylic acid	GC_Serum
858	GCS_858	GCS_salicylaldehyde	GC_Serum
859	GCS_859	GCS_ribonic acid	GC_Serum
860	GCS_860	GCS_ribitol	GC_Serum
861	GCS_861	GCS_pyruvic acid	GC_Serum
862	GCS_862	GCS_propane-1,3-diol NIST	GC_Serum
863	GCS_863	GCS_proline	GC_Serum
864	GCS_864	GCS_pipecolic acid	GC_Serum
865	GCS_865	GCS_phthalic acid	GC_Serum
866	GCS_866	GCS_phosphoric acid	GC_Serum
867	GCS_867	GCS_phosphoethanolamine	GC_Serum
868	GCS_868	GCS_phenylethylamine	GC_Serum
869	GCS_869	GCS_phenylalanine	GC_Serum
870	GCS_870	GCS_phenylacetic acid	GC_Serum
871	GCS_871	GCS_pentadecanoic acid	GC_Serum
872	GCS_872	GCS_pelargonic acid	GC_Serum
873	GCS_873	GCS_paracetamol	GC_Serum
874	GCS_874	GCS_parabanic acid NIST	GC_Serum
875	GCS_875	GCS_pantothenic acid	GC_Serum
876	GCS_876	GCS_palmitoleic acid	GC_Serum
877	GCS_877	GCS_palmitic acid	GC_Serum

878	GCS_878	GCS_oxoproline	GC_Serum
879	GCS_879	GCS_oxalic acid	GC_Serum
880	GCS_880	GCS_ornithine	GC_Serum
881	GCS_881	GCS_oleic acid	GC_Serum
882	GCS_882	GCS_octadecanol	GC_Serum
883	GCS_883	GCS_naproxen	GC_Serum
884	GCS_884	GCS_N-acetylglycine NIST	GC_Serum
885	GCS_885	GCS_N-acetylglutamate	GC_Serum
886	GCS_886	GCS_N-acetyl-D-hexosamine	GC_Serum
887	GCS_887	GCS_myristic acid	GC_Serum
888	GCS_888	GCS_methylhexadecanoic acid	GC_Serum
889	GCS_889	GCS_methionine sulfoxide	GC_Serum
890	GCS_890	GCS_methionine	GC_Serum
891	GCS_891	GCS_methanolphosphate	GC_Serum
892	GCS_892	GCS_malic acid	GC_Serum
893	GCS_893	GCS_maleimide	GC_Serum
894	GCS_894	GCS_lysine	GC_Serum
895	GCS_895	GCS_linolenic acid	GC_Serum
896	GCS_896	GCS_linoleic acid	GC_Serum
897	GCS_897	GCS_leucine	GC_Serum
898	GCS_898	GCS_lauric acid	GC_Serum
899	GCS_899	GCS_lactic acid	GC_Serum
900	GCS_900	GCS_kynurenine	GC_Serum
901	GCS_901	GCS_isorhamnose	GC_Serum
902	GCS_902	GCS_isolinoleic acid NIST	GC_Serum
903	GCS_903	GCS_isoleucine	GC_Serum
904	GCS_904	GCS_isocitric acid	GC_Serum
905	GCS_905	GCS_inosine	GC_Serum
906	GCS_906	GCS_indole-3-lactate	GC_Serum
907	GCS_907	GCS_indole-3-acetate	GC_Serum
908	GCS_908	GCS_idonic acid NIST	GC_Serum
909	GCS_909	GCS_icosenoic acid	GC_Serum
910	GCS_910	GCS_hydroxylamine	GC_Serum
911	GCS_911	GCS_hydrocinnamic acid	GC_Serum
912	GCS_912	GCS_histidine	GC_Serum
913	GCS_913	GCS_hippuric acid	GC_Serum
914	GCS_914	GCS_heptadecanoic acid NIST	GC_Serum
915	GCS_915	GCS_guanosine	GC_Serum
916	GCS_916	GCS_glycolic acid	GC_Serum
917	GCS_917	GCS_glycine	GC_Serum
918	GCS_918	GCS_glycerol-alpha-phosphate	GC_Serum
919	GCS_919	GCS_glyceric acid	GC_Serum
920	GCS_920	GCS_glutaric acid	GC_Serum
921	GCS_921	GCS_glutamine	GC_Serum
922	GCS_922	GCS_glutamic acid	GC_Serum
923	GCS_923	GCS_glucose	GC_Serum
924	GCS_924	GCS_gluconic acid lactone	GC_Serum
925	GCS_925	GCS_gluconic acid	GC_Serum
926	GCS_926	GCS_gamma-tocopherol	GC_Serum
927	GCS_927	GCS_hexuronic acid	GC_Serum
928	GCS_928	GCS_galacturonic acid	GC_Serum
929	GCS_929	GCS_galactose	GC_Serum
930	GCS_930	GCS_GABA	GC_Serum
931	GCS_931	GCS_fumaric acid	GC_Serum
932	GCS_932	GCS_fructose	GC_Serum
933	GCS_933	GCS_ethanolamine	GC_Serum
934	GCS_934	GCS_erythritol	GC_Serum
935	GCS_935	GCS_dopamine NIST	GC_Serum
936	GCS_936	GCS_dodecanol	GC_Serum
937	GCS_937	GCS_dodecane	GC_Serum
938	GCS_938	GCS_dihydroabietic acid	GC_Serum
939	GCS_939	GCS_dihydro-3-coumaric acid	GC_Serum

940	GCS_940	GCS_cytidine-5'-diphosphate	GC_Serum
941	GCS_941	GCS_cystine	GC_Serum
942	GCS_942	GCS_cysteine-glycine	GC_Serum
943	GCS_943	GCS_creatinine	GC_Serum
944	GCS_944	GCS_citrulline	GC_Serum
945	GCS_945	GCS_citric acid	GC_Serum
946	GCS_946	GCS_citramalic acid	GC_Serum
947	GCS_947	GCS_cholesterol	GC_Serum
948	GCS_948	GCS_cellobiose	GC_Serum
949	GCS_949	GCS_caprylic acid	GC_Serum
950	GCS_950	GCS_capric acid	GC_Serum
951	GCS_951	GCS_butyrolactam NIST	GC_Serum
952	GCS_952	GCS_biuret	GC_Serum
953	GCS_953	GCS_beta-sitosterol	GC_Serum
954	GCS_954	GCS_beta-alanine	GC_Serum
955	GCS_955	GCS_benzylalcohol	GC_Serum
956	GCS_956	GCS_benzoic acid	GC_Serum
957	GCS_957	GCS_behenic acid	GC_Serum
958	GCS_958	GCS_azelaic acid	GC_Serum
959	GCS_959	GCS_aspartic acid	GC_Serum
960	GCS_960	GCS_asparagine	GC_Serum
961	GCS_961	GCS_arachidonic acid isomer	GC_Serum
962	GCS_962	GCS_arachidonic acid	GC_Serum
963	GCS_963	GCS_arabinose	GC_Serum
964	GCS_964	GCS_aminomalonic acid	GC_Serum
965	GCS_965	GCS_allantoic acid	GC_Serum
966	GCS_966	GCS_alanine	GC_Serum
967	GCS_967	GCS_adipic acid	GC_Serum
968	GCS_968	GCS_aconitic acid	GC_Serum
969	GCS_969	GCS_5-methoxytryptamine	GC_Serum
970	GCS_970	GCS_5-hydroxyindole-3-acetic acid NIST	GC_Serum
971	GCS_971	GCS_5-deoxyribitol NIST	GC_Serum
972	GCS_972	GCS_4-hydroxyphenylacetic acid	GC_Serum
973	GCS_973	GCS_3-phenyllactic acid	GC_Serum
974	GCS_974	GCS_3-hydroxypropionic acid	GC_Serum
975	GCS_975	GCS_3-hydroxybutanoic acid	GC_Serum
976	GCS_976	GCS_3-aminoisobutyric acid	GC_Serum
977	GCS_977	GCS_2-hydroxyvaleric acid	GC_Serum
978	GCS_978	GCS_2-hydroxyglutaric acid	GC_Serum
979	GCS_979	GCS_2-hydroxybutanoic acid	GC_Serum
980	GCS_980	GCS_2-hydroxy-2-methylbutanoic acid	GC_Serum
981	GCS_981	GCS_2-deoxyribonic acid	GC_Serum
982	GCS_982	GCS_2-deoxyerythritol NIST	GC_Serum
983	GCS_983	GCS_2-aminoadipic acid	GC_Serum
984	GCS_984	GCS_1-monostearin	GC_Serum
985	GCS_985	GCS_1-monopalmitin	GC_Serum
986	GCS_986	GCS_1-monoolein	GC_Serum
987	GCS_987	GCS_1-hexadecanol	GC_Serum
988	GCS_988	GCS_1-deoxyerythritol	GC_Serum
989	GCS_989	GCS_1,5-anhydroglucitol	GC_Serum
990	GCS_990	GCS_206289	GC_Serum
991	GCS_991	GCS_492765	GC_Serum
992	GCS_992	GCS_473067	GC_Serum
993	GCS_993	GCS_213253	GC_Serum
994	GCS_994	GCS_204344	GC_Serum
995	GCS_995	GCS_486090	GC_Serum
996	GCS_996	GCS_438058	GC_Serum
997	GCS_997	GCS_199786	GC_Serum
998	GCS_998	GCS_215397	GC_Serum
999	GCS_999	GCS_446633	GC_Serum
1000	GCS_1000	GCS_199777	GC_Serum
1001	GCS_1001	GCS_216427	GC_Serum

1002	GCS_1002	GCS_367936	GC_Serum
1003	GCS_1003	GCS_486054	GC_Serum
1004	GCS_1004	GCS_299153	GC_Serum
1005	GCS_1005	GCS_438059	GC_Serum
1006	GCS_1006	GCS_485397	GC_Serum
1007	GCS_1007	GCS_306148	GC_Serum
1008	GCS_1008	GCS_368008	GC_Serum
1009	GCS_1009	GCS_213972	GC_Serum
1010	GCS_1010	GCS_425836	GC_Serum
1011	GCS_1011	GCS_408731	GC_Serum
1012	GCS_1012	GCS_273773	GC_Serum
1013	GCS_1013	GCS_486007	GC_Serum
1014	GCS_1014	GCS_212649	GC_Serum
1015	GCS_1015	GCS_308137	GC_Serum
1016	GCS_1016	GCS_446628	GC_Serum
1017	GCS_1017	GCS_438061	GC_Serum
1018	GCS_1018	GCS_446067	GC_Serum
1019	GCS_1019	GCS_486211	GC_Serum
1020	GCS_1020	GCS_301536	GC_Serum
1021	GCS_1021	GCS_223566	GC_Serum
1022	GCS_1022	GCS_486213	GC_Serum
1023	GCS_1023	GCS_306159	GC_Serum
1024	GCS_1024	GCS_210286	GC_Serum
1025	GCS_1025	GCS_474632	GC_Serum
1026	GCS_1026	GCS_300866	GC_Serum
1027	GCS_1027	GCS_309532	GC_Serum
1028	GCS_1028	GCS_337157	GC_Serum
1029	GCS_1029	GCS_474971	GC_Serum
1030	GCS_1030	GCS_273182	GC_Serum
1031	GCS_1031	GCS_228673	GC_Serum
1032	GCS_1032	GCS_486218	GC_Serum
1033	GCS_1033	GCS_408618	GC_Serum
1034	GCS_1034	GCS_484792	GC_Serum
1035	GCS_1035	GCS_199596	GC_Serum
1036	GCS_1036	GCS_273925	GC_Serum
1037	GCS_1037	GCS_486020	GC_Serum
1038	GCS_1038	GCS_200644	GC_Serum
1039	GCS_1039	GCS_455312	GC_Serum
1040	GCS_1040	GCS_486017	GC_Serum
1041	GCS_1041	GCS_307966	GC_Serum
1042	GCS_1042	GCS_356925	GC_Serum
1043	GCS_1043	GCS_206526	GC_Serum
1044	GCS_1044	GCS_242565	GC_Serum
1045	GCS_1045	GCS_486016	GC_Serum
1046	GCS_1046	GCS_226912	GC_Serum
1047	GCS_1047	GCS_214011	GC_Serum
1048	GCS_1048	GCS_485388	GC_Serum
1049	GCS_1049	GCS_473083	GC_Serum
1050	GCS_1050	GCS_308234	GC_Serum
1051	GCS_1051	GCS_225043	GC_Serum
1052	GCS_1052	GCS_404754	GC_Serum
1053	GCS_1053	GCS_477827	GC_Serum
1054	GCS_1054	GCS_227902	GC_Serum
1055	GCS_1055	GCS_221574	GC_Serum
1056	GCS_1056	GCS_309730	GC_Serum
1057	GCS_1057	GCS_479886	GC_Serum
1058	GCS_1058	GCS_222115	GC_Serum
1059	GCS_1059	GCS_228872	GC_Serum
1060	GCS_1060	GCS_200506	GC_Serum
1061	GCS_1061	GCS_212866	GC_Serum
1062	GCS_1062	GCS_438093	GC_Serum
1063	GCS_1063	GCS_208557	GC_Serum

1064	GCS_1064	GCS_465393	GC_Serum
1065	GCS_1065	GCS_338896	GC_Serum
1066	GCS_1066	GCS_214160	GC_Serum
1067	GCS_1067	GCS_362005	GC_Serum
1068	GCS_1068	GCS_217797	GC_Serum
1069	GCS_1069	GCS_214685	GC_Serum
1070	GCS_1070	GCS_213185	GC_Serum
1071	GCS_1071	GCS_207223	GC_Serum
1072	GCS_1072	GCS_224632	GC_Serum
1073	GCS_1073	GCS_226850	GC_Serum
1074	GCS_1074	GCS_289052	GC_Serum
1075	GCS_1075	GCS_408490	GC_Serum
1076	GCS_1076	GCS_200850	GC_Serum
1077	GCS_1077	GCS_213160	GC_Serum
1078	GCS_1078	GCS_485553	GC_Serum
1079	GCS_1079	GCS_407508	GC_Serum
1080	GCS_1080	GCS_486245	GC_Serum
1081	GCS_1081	GCS_272694	GC_Serum
1082	GCS_1082	GCS_414988	GC_Serum
1083	GCS_1083	GCS_223191	GC_Serum
1084	GCS_1084	GCS_455340	GC_Serum
1085	GCS_1085	GCS_222169	GC_Serum
1086	GCS_1086	GCS_415114	GC_Serum
1087	GCS_1087	GCS_495239	GC_Serum
1088	GCS_1088	GCS_218765	GC_Serum
1089	GCS_1089	GCS_223618	GC_Serum
1090	GCS_1090	GCS_213226	GC_Serum
1091	GCS_1091	GCS_309642	GC_Serum
1092	GCS_1092	GCS_200588	GC_Serum
1093	GCS_1093	GCS_415158	GC_Serum
1094	GCS_1094	GCS_428311	GC_Serum
1095	GCS_1095	GCS_204157	GC_Serum
1096	GCS_1096	GCS_415924	GC_Serum
1097	GCS_1097	GCS_231796	GC_Serum
1098	GCS_1098	GCS_203259	GC_Serum
1099	GCS_1099	GCS_415143	GC_Serum
1100	GCS_1100	GCS_237984	GC_Serum
1101	GCS_1101	GCS_309837	GC_Serum
1102	GCS_1102	GCS_307915	GC_Serum
1103	GCS_1103	GCS_223973	GC_Serum
1104	GCS_1104	GCS_273590	GC_Serum
1105	GCS_1105	GCS_214058	GC_Serum
1106	GCS_1106	GCS_238384	GC_Serum
1107	GCS_1107	GCS_269864	GC_Serum
1108	GCS_1108	GCS_474690	GC_Serum
1109	GCS_1109	GCS_218597	GC_Serum
1110	GCS_1110	GCS_272855	GC_Serum
1111	GCS_1111	GCS_337230	GC_Serum
1112	GCS_1112	GCS_228377	GC_Serum
1113	GCS_1113	GCS_232869	GC_Serum
1114	GCS_1114	GCS_203786	GC_Serum
1115	GCS_1115	GCS_288822	GC_Serum
1116	GCS_1116	GCS_201042	GC_Serum
1117	GCS_1117	GCS_236890	GC_Serum
1118	GCS_1118	GCS_211453	GC_Serum
1119	GCS_1119	GCS_225427	GC_Serum
1120	GCS_1120	GCS_337196	GC_Serum
1121	GCS_1121	GCS_217816	GC_Serum
1122	GCS_1122	GCS_200429	GC_Serum
1123	GCS_1123	GCS_455826	GC_Serum
1124	GCS_1124	GCS_273447	GC_Serum
1125	GCS_1125	GCS_475951	GC_Serum

1126	GCS_1126	GCS_285340	GC_Serum
1127	GCS_1127	GCS_470289	GC_Serum
1128	GCS_1128	GCS_232660	GC_Serum
1129	GCS_1129	GCS_486192	GC_Serum
1130	GCS_1130	GCS_213961	GC_Serum
1131	GCS_1131	GCS_483487	GC_Serum
1132	GCS_1132	GCS_227318	GC_Serum
1133	GCS_1133	GCS_280546	GC_Serum
1134	GCS_1134	GCS_267884	GC_Serum
1135	GCS_1135	GCS_213960	GC_Serum
1136	GCS_1136	GCS_301399	GC_Serum
1137	GCS_1137	GCS_220010	GC_Serum
1138	GCS_1138	GCS_228546	GC_Serum
1139	GCS_1139	GCS_304945	GC_Serum
1140	GCS_1140	GCS_214535	GC_Serum
1141	GCS_1141	GCS_202681	GC_Serum
1142	GCS_1142	GCS_486884	GC_Serum
1143	GCS_1143	GCS_486024	GC_Serum
1144	GCS_1144	GCS_281910	GC_Serum
1145	GCS_1145	GCS_218483	GC_Serum
1146	GCS_1146	GCS_211952	GC_Serum
1147	GCS_1147	GCS_300920	GC_Serum
1148	GCS_1148	GCS_231657	GC_Serum
1149	GCS_1149	GCS_239577	GC_Serum
1150	GCS_1150	GCS_309540	GC_Serum
1151	GCS_1151	GCS_219510	GC_Serum
1152	GCS_1152	GCS_223625	GC_Serum
1153	GCS_1153	GCS_225430	GC_Serum
1154	GCS_1154	GCS_216838	GC_Serum
1155	GCS_1155	GCS_213296	GC_Serum
1156	GCS_1156	GCS_241097	GC_Serum
1157	GCS_1157	GCS_268313	GC_Serum
1158	GCS_1158	GCS_225446	GC_Serum
1159	GCS_1159	GCS_225882	GC_Serum
1160	GCS_1160	GCS_484345	GC_Serum
1161	GCS_1161	GCS_218694	GC_Serum
1162	GCS_1162	GCS_204425	GC_Serum
1163	GCS_1163	GCS_486179	GC_Serum
1164	GCS_1164	GCS_218724	GC_Serum
1165	GCS_1165	GCS_404516	GC_Serum
1166	GCS_1166	GCS_339455	GC_Serum
1167	GCS_1167	GCS_321685	GC_Serum
1168	GCS_1168	GCS_208723	GC_Serum
1169	GCS_1169	GCS_309949	GC_Serum
1170	GCS_1170	GCS_218512	GC_Serum
1171	GCS_1171	GCS_328803	GC_Serum
1172	GCS_1172	GCS_227017	GC_Serum
1173	GCS_1173	GCS_239565	GC_Serum
1174	GCS_1174	GCS_445039	GC_Serum
1175	GCS_1175	GCS_213697	GC_Serum
1176	GCS_1176	GCS_302351	GC_Serum
1177	GCS_1177	GCS_223521	GC_Serum
1178	GCS_1178	GCS_485464	GC_Serum
1179	GCS_1179	GCS_227951	GC_Serum
1180	GCS_1180	GCS_236725	GC_Serum
1181	GCS_1181	GCS_474686	GC_Serum
1182	GCS_1182	GCS_308192	GC_Serum
1183	GCS_1183	GCS_310667	GC_Serum
1184	GCS_1184	GCS_470283	GC_Serum
1185	GCS_1185	GCS_273170	GC_Serum
1186	GCS_1186	GCS_218951	GC_Serum
1187	GCS_1187	GCS_206556	GC_Serum

1188	GCS_1188	GCS_357010	GC_Serum
1189	GCS_1189	GCS_227983	GC_Serum
1190	GCS_1190	GCS_268445	GC_Serum
1191	GCS_1191	GCS_295046	GC_Serum
1192	GCS_1192	GCS_201862	GC_Serum
1193	GCS_1193	GCS_226901	GC_Serum
1194	GCS_1194	GCS_200906	GC_Serum
1195	GCS_1195	GCS_357211	GC_Serum
1196	GCS_1196	GCS_327918	GC_Serum
1197	GCS_1197	GCS_223548	GC_Serum
1198	GCS_1198	GCS_428330	GC_Serum
1199	GCS_1199	GCS_476951	GC_Serum
1200	GCS_1200	GCS_241307	GC_Serum
1201	GCS_1201	GCS_356987	GC_Serum
1202	GCS_1202	GCS_356938	GC_Serum
1203	GCS_1203	GCS_228612	GC_Serum
1204	GCS_1204	GCS_274743	GC_Serum
1205	GCS_1205	GCS_228594	GC_Serum
1206	GCS_1206	GCS_281268	GC_Serum
1207	GCS_1207	GCS_269625	GC_Serum
1208	GCS_1208	GCS_480140	GC_Serum
1209	GCS_1209	GCS_227761	GC_Serum
1210	GCS_1210	GCS_221505	GC_Serum
1211	GCS_1211	GCS_208664	GC_Serum
1212	GCS_1212	GCS_201887	GC_Serum
1213	GCS_1213	GCS_233365	GC_Serum
1214	GCS_1214	GCS_241087	GC_Serum
1215	GCS_1215	GCS_277432	GC_Serum
1216	GCS_1216	GCS_231850	GC_Serum
1217	GCS_1217	GCS_388098	GC_Serum
1218	GCS_1218	GCS_241319	GC_Serum
1219	GCS_1219	GCS_233455	GC_Serum
1220	GCS_1220	GCS_220012	GC_Serum
1221	GCS_1221	GCS_205680	GC_Serum
1222	GCS_1222	GCS_229954	GC_Serum
1223	GCS_1223	GCS_268083	GC_Serum
1224	GCS_1224	GCS_199337	GC_Serum
1225	GCS_1225	GCS_295226	GC_Serum
1226	GCS_1226	GCS_300379	GC_Serum
1227	GCS_1227	GCS_280564	GC_Serum
1228	GCS_1228	GCS_321696	GC_Serum
1229	GCS_1229	GCS_268457	GC_Serum
1230	GCS_1230	GCS_231945	GC_Serum
1231	GCS_1231	GCS_223597	GC_Serum
1232	GCS_1232	GCS_215978	GC_Serum
1233	GCS_1233	GCS_211891	GC_Serum
1234	GCS_1234	GCS_211997	GC_Serum
1235	GCS_1235	GCS_267647	GC_Serum
1236	GCS_1236	GCS_274189	GC_Serum
1237	GCS_1237	GCS_225863	GC_Serum
1238	GCS_1238	GCS_416140	GC_Serum
1239	GCS_1239	GCS_271128	GC_Serum
1240	GCS_1240	GCS_437266	GC_Serum
1241	GCS_1241	GCS_330990	GC_Serum
1242	GCS_1242	GCS_359447	GC_Serum
1243	GCS_1243	GCS_438109	GC_Serum
1244	GCS_1244	GCS_272880	GC_Serum
1245	GCS_1245	GCS_232075	GC_Serum
1246	GCS_1246	GCS_242526	GC_Serum
1247	GCS_1247	GCS_207684	GC_Serum
1248	GCS_1248	GCS_268506	GC_Serum
1249	GCS_1249	GCS_222199	GC_Serum

1250	GCS_1250	GCS_299159	GC_Serum
1251	GCS_1251	GCS_233174	GC_Serum
1252	GCS_1252	GCS_200490	GC_Serum
1253	GCS_1253	GCS_268100	GC_Serum
1254	GCS_1254	GCS_226272	GC_Serum
1255	GCS_1255	GCS_228147	GC_Serum
1256	GCS_1256	GCS_331132	GC_Serum
1257	GCS_1257	GCS_276947	GC_Serum
1258	GCS_1258	GCS_309791	GC_Serum
1259	GCS_1259	GCS_215494	GC_Serum
1260	GCS_1260	GCS_368022	GC_Serum
1261	GCS_1261	GCS_330991	GC_Serum
1262	GCS_1262	GCS_233011	GC_Serum
1263	GCS_1263	GCS_281419	GC_Serum
1264	GCS_1264	GCU_xylose 2	GC_Urine
1265	GCS_1265	GCU_xylitol	GC_Urine
1266	GCS_1266	GCU_xanthurenic acid	GC_Urine
1267	GCU_1267	GCU_xanthosine	GC_Urine
1268	GCU_1268	GCU_xanthine	GC_Urine
1269	GCU_1269	GCU_vanillic acid	GC_Urine
1270	GCU_1270	GCU_valine TMS1x	GC_Urine
1271	GCU_1271	GCU_valine	GC_Urine
1272	GCU_1272	GCU_urocanic acid	GC_Urine
1273	GCU_1273	GCU_uric acid (mix spec with myo-inositol)	GC_Urine
1274	GCU_1274	GCU_urea	GC_Urine
1275	GCU_1275	GCU_uracil	GC_Urine
1276	GCU_1276	GCU_UDP-glucuronic acid	GC_Urine
1277	GCU_1277	GCU_tyrosine mz147 missing	GC_Urine
1278	GCU_1278	GCU_tyrosine minor	GC_Urine
1279	GCU_1279	GCU_tryptophan minor	GC_Urine
1280	GCU_1280	GCU_tryptophan	GC_Urine
1281	GCU_1281	GCU_trigonelline NIST	GC_Urine
1282	GCU_1282	GCU_thymine	GC_Urine
1283	GCU_1283	GCU_thymidine 2	GC_Urine
1284	GCU_1284	GCU_threose meox2	GC_Urine
1285	GCU_1285	GCU_threonine minor	GC_Urine
1286	GCU_1286	GCU_threonine	GC_Urine
1287	GCU_1287	GCU_threonic acid 1	GC_Urine
1288	GCU_1288	GCU_tartaric acid	GC_Urine
1289	GCU_1289	GCU_talose	GC_Urine
1290	GCU_1290	GCU_syringic acid	GC_Urine
1291	GCU_1291	GCU_synephrine minor	GC_Urine
1292	GCU_1292	GCU_sulfuric acid	GC_Urine
1293	GCU_1293	GCU_succinic acid	GC_Urine
1294	GCU_1294	GCU_stearic acid	GC_Urine
1295	GCU_1295	GCU_sorbitol	GC_Urine
1296	GCU_1296	GCU_sophorose minor	GC_Urine
1297	GCU_1297	GCU_sophorose	GC_Urine
1298	GCU_1298	GCU_(s)-(+)-mandelic acid	GC_Urine
1299	GCU_1299	GCU_shikimic acid	GC_Urine
1300	GCU_1300	GCU_serine minor	GC_Urine
1301	GCU_1301	GCU_salicylic acid	GC_Urine
1302	GCU_1302	GCU_ribose	GC_Urine
1303	GCU_1303	GCU_ribonic acid	GC_Urine
1304	GCU_1304	GCU_ribitol	GC_Urine
1305	GCU_1305	GCU_rhamnose 1	GC_Urine
1306	GCU_1306	GCU_quinolinic acid	GC_Urine
1307	GCU_1307	GCU_pyruvic acid	GC_Urine
1308	GCU_1308	GCU_pyrophosphate	GC_Urine
1309	GCU_1309	GCU_pyrogallol	GC_Urine
1310	GCU_1310	GCU_propane-1,3-diol NIST	GC_Urine
1311	GCU_1311	GCU_pimelic acid	GC_Urine

1312	GCU_1312	GCU_phthalic acid	GC_Urine
1313	GCU_1313	GCU_phosphoric acid	GC_Urine
1314	GCU_1314	GCU_phosphoric acid	GC_Urine
1315	GCU_1315	GCU_phosphoethanolamine	GC_Urine
1316	GCU_1316	GCU_phenylalanine TMS1x	GC_Urine
1317	GCU_1317	GCU_phenylalanine	GC_Urine
1318	GCU_1318	GCU_phenol	GC_Urine
1319	GCU_1319	GCU_pentitol	GC_Urine
1320	GCU_1320	GCU_pelargonic acid	GC_Urine
1321	GCU_1321	GCU_paracetamol	GC_Urine
1322	GCU_1322	GCU_parabanic acid NIST	GC_Urine
1323	GCU_1323	GCU_pantothenic acid	GC_Urine
1324	GCU_1324	GCU_palmitic acid	GC_Urine
1325	GCU_1325	GCU_palatinose	GC_Urine
1326	GCU_1326	GCU_palatinitol	GC_Urine
1327	GCU_1327	GCU_oxoproline	GC_Urine
1328	GCU_1328	GCU_oxalic acid	GC_Urine
1329	GCU_1329	GCU_orotic acid	GC_Urine
1330	GCU_1330	GCU_oleic acid	GC_Urine
1331	GCU_1331	GCU_octadecanol	GC_Urine
1332	GCU_1332	GCU_noradrenaline	GC_Urine
1333	GCU_1333	GCU_nicotinamide	GC_Urine
1334	GCU_1334	GCU_n-epsilon-trimethyllysine	GC_Urine
1335	GCU_1335	GCU_N-carbamylglutamate	GC_Urine
1336	GCU_1336	GCU_naproxen	GC_Urine
1337	GCU_1337	GCU_N-acetyl-glutamic acid	GC_Urine
1338	GCU_1338	GCU_N-acetylglutamate	GC_Urine
1339	GCU_1339	GCU_N-acetyl-D-mannosamine major	GC_Urine
1340	GCU_1340	GCU_N-acetyl-D-hexosamine	GC_Urine
1341	GCU_1341	GCU_N-acetylaspartic acid 1	GC_Urine
1342	GCU_1342	GCU_myristic acid	GC_Urine
1343	GCU_1343	GCU_methylmaleic acid	GC_Urine
1344	GCU_1344	GCU_methylhexose NIST	GC_Urine
1345	GCU_1345	GCU_methylhexadecanoic acid	GC_Urine
1346	GCU_1346	GCU_methylcitrate	GC_Urine
1347	GCU_1347	GCU_melibiose	GC_Urine
1348	GCU_1348	GCU_mannose-6-phosphate NIST	GC_Urine
1349	GCU_1349	GCU_mannitol	GC_Urine
1350	GCU_1350	GCU_maltose-like	GC_Urine
1351	GCU_1351	GCU_maltose 1	GC_Urine
1352	GCU_1352	GCU_malonic acid	GC_Urine
1353	GCU_1353	GCU_malic acid	GC_Urine
1354	GCU_1354	GCU_maleic acid	GC_Urine
1355	GCU_1355	GCU_lysine	GC_Urine
1356	GCU_1356	GCU_levoglucosan	GC_Urine
1357	GCU_1357	GCU_levanbiose	GC_Urine
1358	GCU_1358	GCU_leucine	GC_Urine
1359	GCU_1359	GCU_lauric acid	GC_Urine
1360	GCU_1360	GCU_lactobionic acid	GC_Urine
1361	GCU_1361	GCU_lactic acid	GC_Urine
1362	GCU_1362	GCU_kynurenine	GC_Urine
1363	GCU_1363	GCU_isorhamnose	GC_Urine
1364	GCU_1364	GCU_isoleucine minor	GC_Urine
1365	GCU_1365	GCU_isocitric lactone	GC_Urine
1366	GCU_1366	GCU_isocitric acid	GC_Urine
1367	GCU_1367	GCU_inositol myo-	GC_Urine
1368	GCU_1368	GCU_inosine	GC_Urine
1369	GCU_1369	GCU_indole-3-lactate	GC_Urine
1370	GCU_1370	GCU_indole-3-acetate	GC_Urine
1371	GCU_1371	GCU_idonic acid NIST	GC_Urine
1372	GCU_1372	GCU_hydroxylamine	GC_Urine
1373	GCU_1373	GCU_homovanillic and 4-hydroxymandelic acid - mixed spectrum	GC_Urine

1374	GCU_1374	GCU_homoserine	GC_Urine
1375	GCU_1375	GCU_homogentisic acid	GC_Urine
1376	GCU_1376	GCU_histidine	GC_Urine
1377	GCU_1377	GCU_hippuric acid 1TMS	GC_Urine
1378	GCU_1378	GCU_guanine	GC_Urine
1379	GCU_1379	GCU_glyoxalurea NIST	GC_Urine
1380	GCU_1380	GCU_glycyl proline	GC_Urine
1381	GCU_1381	GCU_glycolic acid	GC_Urine
1382	GCU_1382	GCU_glycocyamine major	GC_Urine
1383	GCU_1383	GCU_glycine TMS1x	GC_Urine
1384	GCU_1384	GCU_glycerol-alpha-phosphate	GC_Urine
1385	GCU_1385	GCU_glyceric acid	GC_Urine
1386	GCU_1386	GCU_glutaric acid	GC_Urine
1387	GCU_1387	GCU_glutamine dehydrated 2TMS minor	GC_Urine
1388	GCU_1388	GCU_glutamine	GC_Urine
1389	GCU_1389	GCU_glutamate TMS2x	GC_Urine
1390	GCU_1390	GCU_glucose 2	GC_Urine
1391	GCU_1391	GCU_glucose-1-phosphate	GC_Urine
1392	GCU_1392	GCU_glucose 1	GC_Urine
1393	GCU_1393	GCU_gluconic acid lactone	GC_Urine
1394	GCU_1394	GCU_gluconic acid	GC_Urine
1395	GCU_1395	GCU_galacturonic acid 2	GC_Urine
1396	GCU_1396	GCU_galacturonic acid	GC_Urine
1397	GCU_1397	GCU_galactose	GC_Urine
1398	GCU_1398	GCU_galactonic acid	GC_Urine
1399	GCU_1399	GCU_galactitol	GC_Urine
1400	GCU_1400	GCU_galactinol minor 2	GC_Urine
1401	GCU_1401	GCU_galactinol major 2	GC_Urine
1402	GCU_1402	GCU_galactinol 3	GC_Urine
1403	GCU_1403	GCU_galactinol 2	GC_Urine
1404	GCU_1404	GCU_galactinol 1	GC_Urine
1405	GCU_1405	GCU_furoylglycine NIST	GC_Urine
1406	GCU_1406	GCU_fumaric acid	GC_Urine
1407	GCU_1407	GCU_fucose	GC_Urine
1408	GCU_1408	GCU_fructose 2	GC_Urine
1409	GCU_1409	GCU_fructose 1	GC_Urine
1410	GCU_1410	GCU_FAME marker very close to sorbitol	GC_Urine
1411	GCU_1411	GCU_ethanolamine	GC_Urine
1412	GCU_1412	GCU_erythronic acid lactone	GC_Urine
1413	GCU_1413	GCU_erythronic acid lactone	GC_Urine
1414	GCU_1414	GCU_erythritol	GC_Urine
1415	GCU_1415	GCU_enolpyruvate NIST	GC_Urine
1416	GCU_1416	GCU_dodecane	GC_Urine
1417	GCU_1417	GCU_DL-4-hydroxymandelonitrile major _RI570313	GC_Urine
1418	GCU_1418	GCU_dihydro-3-coumaric acid	GC_Urine
1419	GCU_1419	GCU_digalacturonic acid	GC_Urine
1420	GCU_1420	GCU_dehydroascorbic acid	GC_Urine
1421	GCU_1421	GCU_dehydroascorbate 3	GC_Urine
1422	GCU_1422	GCU_cytidine-5-monophosphate NIST	GC_Urine
1423	GCU_1423	GCU_cystine minor	GC_Urine
1424	GCU_1424	GCU_cystine	GC_Urine
1425	GCU_1425	GCU_cysteine	GC_Urine
1426	GCU_1426	GCU_cyclohexylamine NIST	GC_Urine
1427	GCU_1427	GCU_creatinine	GC_Urine
1428	GCU_1428	GCU_coniferin NIST	GC_Urine
1429	GCU_1429	GCU_citrulline	GC_Urine
1430	GCU_1430	GCU_citric acid	GC_Urine
1431	GCU_1431	GCU_citramalic acid	GC_Urine
1432	GCU_1432	GCU_chlorogenic acid	GC_Urine
1433	GCU_1433	GCU_cellobiotol	GC_Urine
1434	GCU_1434	GCU_cellobiose	GC_Urine
1435	GCU_1435	GCU_caprylic acid	GC_Urine

1436	GCU_1436	GCU_capric acid	GC_Urine
1437	GCU_1437	GCU_caffeic acid	GC_Urine
1438	GCU_1438	GCU_butyrolactam NIST	GC_Urine
1439	GCU_1439	GCU_butane-2,3-diol (NIST)	GC_Urine
1440	GCU_1440	GCU_beta-gentiobiose minor	GC_Urine
1441	GCU_1441	GCU_beta-gentiobiose	GC_Urine
1442	GCU_1442	GCU_beta-gentiobiose	GC_Urine
1443	GCU_1443	GCU_beta-alanine	GC_Urine
1444	GCU_1444	GCU_benzylalcohol	GC_Urine
1445	GCU_1445	GCU_benzoic acid mix spec	GC_Urine
1446	GCU_1446	GCU_azelaic acid	GC_Urine
1447	GCU_1447	GCU_aspartic acid	GC_Urine
1448	GCU_1448	GCU_aspartate minor	GC_Urine
1449	GCU_1449	GCU_asparagine dehydrated	GC_Urine
1450	GCU_1450	GCU_asparagine	GC_Urine
1451	GCU_1451	GCU_ascorbic acid	GC_Urine
1452	GCU_1452	GCU_arabitol	GC_Urine
1453	GCU_1453	GCU_arabinose	GC_Urine
1454	GCU_1454	GCU_aminomalonic acid	GC_Urine
1455	GCU_1455	GCU_allantoic acid -H2O	GC_Urine
1456	GCU_1456	GCU_alanine	GC_Urine
1457	GCU_1457	GCU_adipic acid	GC_Urine
1458	GCU_1458	GCU_adenosine	GC_Urine
1459	GCU_1459	GCU_adenine	GC_Urine
1460	GCU_1460	GCU_aconitic acid	GC_Urine
1461	GCU_1461	GCU_acetophenone NIST	GC_Urine
1462	GCU_1462	GCU_7-methylguanine NIST	GC_Urine
1463	GCU_1463	GCU_6-deoxyglucitol NIST	GC_Urine
1464	GCU_1464	GCU_5-methoxytryptamine	GC_Urine
1465	GCU_1465	GCU_5-hydroxymethyl-2-furoic acid NIST	GC_Urine
1466	GCU_1466	GCU_5-hydroxyindole-3-acetic acid NIST	GC_Urine
1467	GCU_1467	GCU_5-hydroxy-3-indoleacetic acid	GC_Urine
1468	GCU_1468	GCU_5'-deoxy-5'-methylthioadenosine	GC_Urine
1469	GCU_1469	GCU_5-aminovaleric acid lactame	GC_Urine
1470	GCU_1470	GCU_5,6-dihydrouracil	GC_Urine
1471	GCU_1471	GCU_4-hydroxyphenyllactic acid	GC_Urine
1472	GCU_1472	GCU_4-hydroxyphenylacetic acid	GC_Urine
1473	GCU_1473	GCU_4-hydroxyhippuric acid NIST	GC_Urine
1474	GCU_1474	GCU_4-hydroxybutyric acid	GC_Urine
1475	GCU_1475	GCU_4-hydroxybenzoate	GC_Urine
1476	GCU_1476	GCU_2-hydroxyvaleric acid	GC_Urine
1477	GCU_1477	GCU_2-hydroxy-2-methylbutanoic acid	GC_Urine
1478	GCU_1478	GCU_2-hydroxybutanoic acid	GC_Urine
1479	GCU_1479	GCU_3-hydroxybutanoic acid mix spec	GC_Urine
1480	GCU_1480	GCU_3-hydroxypyridine	GC_Urine
1481	GCU_1481	GCU_3-phosphoglycerate	GC_Urine
1482	GCU_1482	GCU_3-hydroxypropionic acid	GC_Urine
1483	GCU_1483	GCU_1-methylinosine NIST	GC_Urine
1484	GCU_1484	GCU_1,5-anhydroglucitol	GC_Urine
1485	GCU_1485	GCU_3-aminoisobutyric acid 1	GC_Urine
1486	GCU_1486	GCU_3-aminoisobutyric acid	GC_Urine
1487	GCU_1487	GCU_3,4-dihydroxyphenylacetic acid	GC_Urine
1488	GCU_1488	GCU_2-isopropylmalic acid	GC_Urine
1489	GCU_1489	GCU_2-aminoadipic acid	GC_Urine
1490	GCU_1490	GCU_2-hydroxyglutaric acid	GC_Urine
1491	GCU_1491	GCU_3-Arylcarbonyl-alanine NIST	GC_Urine
1492	GCU_1492	GCU_3,4-dihydroxyhydrocinnamic acid NIST	GC_Urine
1493	GCU_1493	GCU_1-methyladenosine	GC_Urine
1494	GCU_1494	GCU_2-monostearin NIST	GC_Urine
1495	GCU_1495	GCU_2-deoxyribonic acid	GC_Urine
1496	GCU_1496	GCU_3-methyl-1-oxobutylglycine NIST	GC_Urine
1497	GCU_1497	GCU_2-deoxyerythritol NIST	GC_Urine

1498	GCU_1498	GCU_3-hydroxy-3-indoleacetic acid	GC_Urine
1499	GCU_1499	GCU_3-hydroxy-3-methylglutaric acid	GC_Urine
1500	GCU_1500	GCU_2-deoxytetronic acid NIST	GC_Urine
1501	GCU_1501	GCU_2-deoxytetronic acid	GC_Urine
1502	GCU_1502	GCU_3-ureidopropionate	GC_Urine
1503	GCU_1503	GCU_1,2,4-benzenetriol	GC_Urine
1504	GCU_1504	GCU_3-(3-hydroxyphenyl)-3-hydroxypropionic acid NIST	GC_Urine
1505	GCU_1505	GCU_2-hydroxyhippuric acid	GC_Urine
1506	GCU_1506	GCU_227983	GC_Urine
1507	GCU_1507	GCU_225871	GC_Urine
1508	GCU_1508	GCU_308347	GC_Urine
1509	GCU_1509	GCU_483228	GC_Urine
1510	GCU_1510	GCU_483175	GC_Urine
1511	GCU_1511	GCU_483171	GC_Urine
1512	GCU_1512	GCU_480180	GC_Urine
1513	GCU_1513	GCU_480140	GC_Urine
1514	GCU_1514	GCU_480136	GC_Urine
1515	GCU_1515	GCU_480050	GC_Urine
1516	GCU_1516	GCU_479939	GC_Urine
1517	GCU_1517	GCU_479886	GC_Urine
1518	GCU_1518	GCU_477827	GC_Urine
1519	GCU_1519	GCU_476079	GC_Urine
1520	GCU_1520	GCU_476001	GC_Urine
1521	GCU_1521	GCU_475976	GC_Urine
1522	GCU_1522	GCU_475968	GC_Urine
1523	GCU_1523	GCU_475951	GC_Urine
1524	GCU_1524	GCU_474686	GC_Urine
1525	GCU_1525	GCU_473067	GC_Urine
1526	GCU_1526	GCU_470527	GC_Urine
1527	GCU_1527	GCU_470290	GC_Urine
1528	GCU_1528	GCU_470289	GC_Urine
1529	GCU_1529	GCU_470283	GC_Urine
1530	GCU_1530	GCU_470270	GC_Urine
1531	GCU_1531	GCU_466853	GC_Urine
1532	GCU_1532	GCU_466167	GC_Urine
1533	GCU_1533	GCU_462485	GC_Urine
1534	GCU_1534	GCU_460930	GC_Urine
1535	GCU_1535	GCU_455826	GC_Urine
1536	GCU_1536	GCU_448997	GC_Urine
1537	GCU_1537	GCU_446665	GC_Urine
1538	GCU_1538	GCU_446633	GC_Urine
1539	GCU_1539	GCU_446628	GC_Urine
1540	GCU_1540	GCU_446067	GC_Urine
1541	GCU_1541	GCU_445039	GC_Urine
1542	GCU_1542	GCU_438061	GC_Urine
1543	GCU_1543	GCU_438058	GC_Urine
1544	GCU_1544	GCU_437471	GC_Urine
1545	GCU_1545	GCU_437284	GC_Urine
1546	GCU_1546	GCU_437266	GC_Urine
1547	GCU_1547	GCU_429880	GC_Urine
1548	GCU_1548	GCU_428330	GC_Urine
1549	GCU_1549	GCU_428311	GC_Urine
1550	GCU_1550	GCU_425836	GC_Urine
1551	GCU_1551	GCU_424905	GC_Urine
1552	GCU_1552	GCU_420446	GC_Urine
1553	GCU_1553	GCU_419655	GC_Urine
1554	GCU_1554	GCU_417631	GC_Urine
1555	GCU_1555	GCU_417496	GC_Urine
1556	GCU_1556	GCU_416114	GC_Urine
1557	GCU_1557	GCU_415158	GC_Urine
1558	GCU_1558	GCU_411083	GC_Urine
1559	GCU_1559	GCU_411049	GC_Urine

1560	GCU_1560	GCU_409349	GC_Urine
1561	GCU_1561	GCU_408731	GC_Urine
1562	GCU_1562	GCU_408618	GC_Urine
1563	GCU_1563	GCU_408490	GC_Urine
1564	GCU_1564	GCU_407536	GC_Urine
1565	GCU_1565	GCU_407508	GC_Urine
1566	GCU_1566	GCU_404516	GC_Urine
1567	GCU_1567	GCU_404287	GC_Urine
1568	GCU_1568	GCU_400671	GC_Urine
1569	GCU_1569	GCU_393241	GC_Urine
1570	GCU_1570	GCU_393073	GC_Urine
1571	GCU_1571	GCU_393031	GC_Urine
1572	GCU_1572	GCU_385112	GC_Urine
1573	GCU_1573	GCU_385028	GC_Urine
1574	GCU_1574	GCU_384918	GC_Urine
1575	GCU_1575	GCU_384908	GC_Urine
1576	GCU_1576	GCU_382318	GC_Urine
1577	GCU_1577	GCU_375558	GC_Urine
1578	GCU_1578	GCU_374912	GC_Urine
1579	GCU_1579	GCU_374901	GC_Urine
1580	GCU_1580	GCU_374786	GC_Urine
1581	GCU_1581	GCU_373743	GC_Urine
1582	GCU_1582	GCU_373725	GC_Urine
1583	GCU_1583	GCU_372993	GC_Urine
1584	GCU_1584	GCU_369589	GC_Urine
1585	GCU_1585	GCU_368323	GC_Urine
1586	GCU_1586	GCU_368170	GC_Urine
1587	GCU_1587	GCU_368056	GC_Urine
1588	GCU_1588	GCU_368034	GC_Urine
1589	GCU_1589	GCU_368028	GC_Urine
1590	GCU_1590	GCU_368026	GC_Urine
1591	GCU_1591	GCU_368021	GC_Urine
1592	GCU_1592	GCU_368012	GC_Urine
1593	GCU_1593	GCU_368008	GC_Urine
1594	GCU_1594	GCU_368004	GC_Urine
1595	GCU_1595	GCU_367999	GC_Urine
1596	GCU_1596	GCU_367997	GC_Urine
1597	GCU_1597	GCU_367993	GC_Urine
1598	GCU_1598	GCU_367979	GC_Urine
1599	GCU_1599	GCU_367954	GC_Urine
1600	GCU_1600	GCU_367950	GC_Urine
1601	GCU_1601	GCU_367924	GC_Urine
1602	GCU_1602	GCU_367923	GC_Urine
1603	GCU_1603	GCU_362147	GC_Urine
1604	GCU_1604	GCU_362120	GC_Urine
1605	GCU_1605	GCU_362088	GC_Urine
1606	GCU_1606	GCU_362075	GC_Urine
1607	GCU_1607	GCU_362015	GC_Urine
1608	GCU_1608	GCU_362005	GC_Urine
1609	GCU_1609	GCU_359507	GC_Urine
1610	GCU_1610	GCU_359447	GC_Urine
1611	GCU_1611	GCU_358726	GC_Urine
1612	GCU_1612	GCU_358146	GC_Urine
1613	GCU_1613	GCU_357685	GC_Urine
1614	GCU_1614	GCU_357492	GC_Urine
1615	GCU_1615	GCU_357337	GC_Urine
1616	GCU_1616	GCU_357304	GC_Urine
1617	GCU_1617	GCU_357211	GC_Urine
1618	GCU_1618	GCU_357045	GC_Urine
1619	GCU_1619	GCU_357010	GC_Urine
1620	GCU_1620	GCU_356938	GC_Urine
1621	GCU_1621	GCU_353499	GC_Urine

1622	GCU_1622	GCU_352777	GC_Urine
1623	GCU_1623	GCU_352750	GC_Urine
1624	GCU_1624	GCU_352745	GC_Urine
1625	GCU_1625	GCU_352678	GC_Urine
1626	GCU_1626	GCU_350773	GC_Urine
1627	GCU_1627	GCU_349887	GC_Urine
1628	GCU_1628	GCU_349090	GC_Urine
1629	GCU_1629	GCU_349036	GC_Urine
1630	GCU_1630	GCU_349029	GC_Urine
1631	GCU_1631	GCU_349005	GC_Urine
1632	GCU_1632	GCU_348977	GC_Urine
1633	GCU_1633	GCU_348903	GC_Urine
1634	GCU_1634	GCU_348900	GC_Urine
1635	GCU_1635	GCU_339455	GC_Urine
1636	GCU_1636	GCU_339205	GC_Urine
1637	GCU_1637	GCU_338896	GC_Urine
1638	GCU_1638	GCU_338773	GC_Urine
1639	GCU_1639	GCU_338711	GC_Urine
1640	GCU_1640	GCU_338465	GC_Urine
1641	GCU_1641	GCU_337230	GC_Urine
1642	GCU_1642	GCU_337159	GC_Urine
1643	GCU_1643	GCU_337157	GC_Urine
1644	GCU_1644	GCU_335989	GC_Urine
1645	GCU_1645	GCU_330609	GC_Urine
1646	GCU_1646	GCU_328803	GC_Urine
1647	GCU_1647	GCU_328506	GC_Urine
1648	GCU_1648	GCU_327665	GC_Urine
1649	GCU_1649	GCU_326566	GC_Urine
1650	GCU_1650	GCU_326500	GC_Urine
1651	GCU_1651	GCU_324627	GC_Urine
1652	GCU_1652	GCU_323640	GC_Urine
1653	GCU_1653	GCU_322260	GC_Urine
1654	GCU_1654	GCU_322204	GC_Urine
1655	GCU_1655	GCU_322199	GC_Urine
1656	GCU_1656	GCU_321896	GC_Urine
1657	GCU_1657	GCU_321748	GC_Urine
1658	GCU_1658	GCU_321716	GC_Urine
1659	GCU_1659	GCU_321703	GC_Urine
1660	GCU_1660	GCU_321696	GC_Urine
1661	GCU_1661	GCU_321693	GC_Urine
1662	GCU_1662	GCU_321685	GC_Urine
1663	GCU_1663	GCU_321533	GC_Urine
1664	GCU_1664	GCU_320562	GC_Urine
1665	GCU_1665	GCU_320489	GC_Urine
1666	GCU_1666	GCU_320207	GC_Urine
1667	GCU_1667	GCU_320146	GC_Urine
1668	GCU_1668	GCU_319935	GC_Urine
1669	GCU_1669	GCU_319923	GC_Urine
1670	GCU_1670	GCU_319536	GC_Urine
1671	GCU_1671	GCU_319370	GC_Urine
1672	GCU_1672	GCU_318794	GC_Urine
1673	GCU_1673	GCU_318770	GC_Urine
1674	GCU_1674	GCU_318412	GC_Urine
1675	GCU_1675	GCU_312592	GC_Urine
1676	GCU_1676	GCU_312139	GC_Urine
1677	GCU_1677	GCU_311085	GC_Urine
1678	GCU_1678	GCU_311071	GC_Urine
1679	GCU_1679	GCU_311066	GC_Urine
1680	GCU_1680	GCU_311029	GC_Urine
1681	GCU_1681	GCU_310988	GC_Urine
1682	GCU_1682	GCU_310904	GC_Urine
1683	GCU_1683	GCU_310785	GC_Urine

1684	GCU_1684	GCU_310752	GC_Urine
1685	GCU_1685	GCU_310634	GC_Urine
1686	GCU_1686	GCU_310592	GC_Urine
1687	GCU_1687	GCU_310461	GC_Urine
1688	GCU_1688	GCU_310408	GC_Urine
1689	GCU_1689	GCU_310380	GC_Urine
1690	GCU_1690	GCU_310240	GC_Urine
1691	GCU_1691	GCU_310063	GC_Urine
1692	GCU_1692	GCU_310046	GC_Urine
1693	GCU_1693	GCU_310006	GC_Urine
1694	GCU_1694	GCU_309837	GC_Urine
1695	GCU_1695	GCU_309830	GC_Urine
1696	GCU_1696	GCU_309829	GC_Urine
1697	GCU_1697	GCU_309725	GC_Urine
1698	GCU_1698	GCU_309642	GC_Urine
1699	GCU_1699	GCU_309631	GC_Urine
1700	GCU_1700	GCU_309626	GC_Urine
1701	GCU_1701	GCU_309591	GC_Urine
1702	GCU_1702	GCU_309584	GC_Urine
1703	GCU_1703	GCU_308547	GC_Urine
1704	GCU_1704	GCU_308452	GC_Urine
1705	GCU_1705	GCU_308387	GC_Urine
1706	GCU_1706	GCU_308370	GC_Urine
1707	GCU_1707	GCU_308350	GC_Urine
1708	GCU_1708	GCU_308328	GC_Urine
1709	GCU_1709	GCU_308308	GC_Urine
1710	GCU_1710	GCU_308270	GC_Urine
1711	GCU_1711	GCU_308244	GC_Urine
1712	GCU_1712	GCU_308227	GC_Urine
1713	GCU_1713	GCU_308220	GC_Urine
1714	GCU_1714	GCU_308210	GC_Urine
1715	GCU_1715	GCU_308192	GC_Urine
1716	GCU_1716	GCU_308188	GC_Urine
1717	GCU_1717	GCU_308158	GC_Urine
1718	GCU_1718	GCU_308106	GC_Urine
1719	GCU_1719	GCU_307915	GC_Urine
1720	GCU_1720	GCU_306148	GC_Urine
1721	GCU_1721	GCU_306145	GC_Urine
1722	GCU_1722	GCU_305306	GC_Urine
1723	GCU_1723	GCU_305055	GC_Urine
1724	GCU_1724	GCU_304945	GC_Urine
1725	GCU_1725	GCU_304847	GC_Urine
1726	GCU_1726	GCU_304770	GC_Urine
1727	GCU_1727	GCU_303163	GC_Urine
1728	GCU_1728	GCU_303152	GC_Urine
1729	GCU_1729	GCU_303091	GC_Urine
1730	GCU_1730	GCU_303066	GC_Urine
1731	GCU_1731	GCU_303060	GC_Urine
1732	GCU_1732	GCU_302341	GC_Urine
1733	GCU_1733	GCU_302328	GC_Urine
1734	GCU_1734	GCU_302325	GC_Urine
1735	GCU_1735	GCU_301755	GC_Urine
1736	GCU_1736	GCU_301583	GC_Urine
1737	GCU_1737	GCU_301570	GC_Urine
1738	GCU_1738	GCU_301565	GC_Urine
1739	GCU_1739	GCU_301536	GC_Urine
1740	GCU_1740	GCU_300919	GC_Urine
1741	GCU_1741	GCU_300866	GC_Urine
1742	GCU_1742	GCU_300663	GC_Urine
1743	GCU_1743	GCU_300661	GC_Urine
1744	GCU_1744	GCU_300626	GC_Urine
1745	GCU_1745	GCU_300481	GC_Urine

1746	GCU_1746	GCU_300379	GC_Urine
1747	GCU_1747	GCU_300353	GC_Urine
1748	GCU_1748	GCU_300348	GC_Urine
1749	GCU_1749	GCU_299859	GC_Urine
1750	GCU_1750	GCU_296113	GC_Urine
1751	GCU_1751	GCU_296081	GC_Urine
1752	GCU_1752	GCU_295269	GC_Urine
1753	GCU_1753	GCU_295226	GC_Urine
1754	GCU_1754	GCU_295150	GC_Urine
1755	GCU_1755	GCU_294988	GC_Urine
1756	GCU_1756	GCU_294826	GC_Urine
1757	GCU_1757	GCU_294799	GC_Urine
1758	GCU_1758	GCU_294795	GC_Urine
1759	GCU_1759	GCU_294621	GC_Urine
1760	GCU_1760	GCU_294547	GC_Urine
1761	GCU_1761	GCU_294511	GC_Urine
1762	GCU_1762	GCU_294462	GC_Urine
1763	GCU_1763	GCU_294456	GC_Urine
1764	GCU_1764	GCU_294129	GC_Urine
1765	GCU_1765	GCU_293873	GC_Urine
1766	GCU_1766	GCU_293824	GC_Urine
1767	GCU_1767	GCU_293814	GC_Urine
1768	GCU_1768	GCU_293688	GC_Urine
1769	GCU_1769	GCU_293097	GC_Urine
1770	GCU_1770	GCU_289827	GC_Urine
1771	GCU_1771	GCU_289055	GC_Urine
1772	GCU_1772	GCU_288966	GC_Urine
1773	GCU_1773	GCU_288892	GC_Urine
1774	GCU_1774	GCU_288846	GC_Urine
1775	GCU_1775	GCU_288375	GC_Urine
1776	GCU_1776	GCU_288351	GC_Urine
1777	GCU_1777	GCU_288331	GC_Urine
1778	GCU_1778	GCU_286873	GC_Urine
1779	GCU_1779	GCU_286263	GC_Urine
1780	GCU_1780	GCU_285189	GC_Urine
1781	GCU_1781	GCU_285065	GC_Urine
1782	GCU_1782	GCU_284611	GC_Urine
1783	GCU_1783	GCU_281398	GC_Urine
1784	GCU_1784	GCU_281386	GC_Urine
1785	GCU_1785	GCU_280705	GC_Urine
1786	GCU_1786	GCU_280553	GC_Urine
1787	GCU_1787	GCU_280546	GC_Urine
1788	GCU_1788	GCU_278207	GC_Urine
1789	GCU_1789	GCU_277432	GC_Urine
1790	GCU_1790	GCU_275375	GC_Urine
1791	GCU_1791	GCU_274454	GC_Urine
1792	GCU_1792	GCU_273925	GC_Urine
1793	GCU_1793	GCU_272849	GC_Urine
1794	GCU_1794	GCU_271143	GC_Urine
1795	GCU_1795	GCU_271063	GC_Urine
1796	GCU_1796	GCU_271008	GC_Urine
1797	GCU_1797	GCU_270547	GC_Urine
1798	GCU_1798	GCU_270251	GC_Urine
1799	GCU_1799	GCU_270080	GC_Urine
1800	GCU_1800	GCU_269870	GC_Urine
1801	GCU_1801	GCU_269852	GC_Urine
1802	GCU_1802	GCU_269776	GC_Urine
1803	GCU_1803	GCU_269689	GC_Urine
1804	GCU_1804	GCU_269648	GC_Urine
1805	GCU_1805	GCU_269625	GC_Urine
1806	GCU_1806	GCU_269261	GC_Urine
1807	GCU_1807	GCU_269146	GC_Urine

1808	GCU_1808	GCU_268716	GC_Urine
1809	GCU_1809	GCU_268610	GC_Urine
1810	GCU_1810	GCU_268506	GC_Urine
1811	GCU_1811	GCU_268437	GC_Urine
1812	GCU_1812	GCU_268365	GC_Urine
1813	GCU_1813	GCU_268326	GC_Urine
1814	GCU_1814	GCU_268316	GC_Urine
1815	GCU_1815	GCU_268313	GC_Urine
1816	GCU_1816	GCU_268167	GC_Urine
1817	GCU_1817	GCU_268165	GC_Urine
1818	GCU_1818	GCU_268148	GC_Urine
1819	GCU_1819	GCU_268141	GC_Urine
1820	GCU_1820	GCU_268110	GC_Urine
1821	GCU_1821	GCU_268106	GC_Urine
1822	GCU_1822	GCU_268103	GC_Urine
1823	GCU_1823	GCU_268100	GC_Urine
1824	GCU_1824	GCU_268093	GC_Urine
1825	GCU_1825	GCU_268083	GC_Urine
1826	GCU_1826	GCU_268050	GC_Urine
1827	GCU_1827	GCU_268042	GC_Urine
1828	GCU_1828	GCU_268026	GC_Urine
1829	GCU_1829	GCU_268025	GC_Urine
1830	GCU_1830	GCU_268024	GC_Urine
1831	GCU_1831	GCU_268007	GC_Urine
1832	GCU_1832	GCU_268006	GC_Urine
1833	GCU_1833	GCU_268005	GC_Urine
1834	GCU_1834	GCU_267987	GC_Urine
1835	GCU_1835	GCU_267972	GC_Urine
1836	GCU_1836	GCU_267958	GC_Urine
1837	GCU_1837	GCU_267946	GC_Urine
1838	GCU_1838	GCU_267926	GC_Urine
1839	GCU_1839	GCU_267923	GC_Urine
1840	GCU_1840	GCU_267904	GC_Urine
1841	GCU_1841	GCU_267898	GC_Urine
1842	GCU_1842	GCU_267893	GC_Urine
1843	GCU_1843	GCU_267890	GC_Urine
1844	GCU_1844	GCU_267886	GC_Urine
1845	GCU_1845	GCU_267884	GC_Urine
1846	GCU_1846	GCU_267882	GC_Urine
1847	GCU_1847	GCU_267880	GC_Urine
1848	GCU_1848	GCU_267872	GC_Urine
1849	GCU_1849	GCU_267871	GC_Urine
1850	GCU_1850	GCU_267870	GC_Urine
1851	GCU_1851	GCU_267868	GC_Urine
1852	GCU_1852	GCU_267851	GC_Urine
1853	GCU_1853	GCU_267850	GC_Urine
1854	GCU_1854	GCU_267842	GC_Urine
1855	GCU_1855	GCU_267833	GC_Urine
1856	GCU_1856	GCU_267830	GC_Urine
1857	GCU_1857	GCU_267824	GC_Urine
1858	GCU_1858	GCU_267816	GC_Urine
1859	GCU_1859	GCU_267808	GC_Urine
1860	GCU_1860	GCU_267787	GC_Urine
1861	GCU_1861	GCU_267785	GC_Urine
1862	GCU_1862	GCU_267774	GC_Urine
1863	GCU_1863	GCU_267771	GC_Urine
1864	GCU_1864	GCU_267769	GC_Urine
1865	GCU_1865	GCU_267768	GC_Urine
1866	GCU_1866	GCU_267762	GC_Urine
1867	GCU_1867	GCU_267760	GC_Urine
1868	GCU_1868	GCU_267757	GC_Urine
1869	GCU_1869	GCU_267756	GC_Urine

1870	GCU_1870	GCU_267755	GC_Urine
1871	GCU_1871	GCU_267751	GC_Urine
1872	GCU_1872	GCU_267749	GC_Urine
1873	GCU_1873	GCU_267748	GC_Urine
1874	GCU_1874	GCU_267742	GC_Urine
1875	GCU_1875	GCU_267740	GC_Urine
1876	GCU_1876	GCU_267737	GC_Urine
1877	GCU_1877	GCU_267730	GC_Urine
1878	GCU_1878	GCU_267727	GC_Urine
1879	GCU_1879	GCU_267723	GC_Urine
1880	GCU_1880	GCU_267721	GC_Urine
1881	GCU_1881	GCU_267715	GC_Urine
1882	GCU_1882	GCU_267714	GC_Urine
1883	GCU_1883	GCU_267707	GC_Urine
1884	GCU_1884	GCU_267704	GC_Urine
1885	GCU_1885	GCU_267703	GC_Urine
1886	GCU_1886	GCU_267701	GC_Urine
1887	GCU_1887	GCU_267698	GC_Urine
1888	GCU_1888	GCU_267696	GC_Urine
1889	GCU_1889	GCU_267695	GC_Urine
1890	GCU_1890	GCU_267692	GC_Urine
1891	GCU_1891	GCU_267691	GC_Urine
1892	GCU_1892	GCU_267687	GC_Urine
1893	GCU_1893	GCU_267686	GC_Urine
1894	GCU_1894	GCU_267682	GC_Urine
1895	GCU_1895	GCU_267676	GC_Urine
1896	GCU_1896	GCU_267675	GC_Urine
1897	GCU_1897	GCU_267674	GC_Urine
1898	GCU_1898	GCU_267673	GC_Urine
1899	GCU_1899	GCU_267671	GC_Urine
1900	GCU_1900	GCU_267670	GC_Urine
1901	GCU_1901	GCU_267669	GC_Urine
1902	GCU_1902	GCU_267665	GC_Urine
1903	GCU_1903	GCU_267661	GC_Urine
1904	GCU_1904	GCU_267656	GC_Urine
1905	GCU_1905	GCU_267653	GC_Urine
1906	GCU_1906	GCU_267652	GC_Urine
1907	GCU_1907	GCU_267649	GC_Urine
1908	GCU_1908	GCU_267647	GC_Urine
1909	GCU_1909	GCU_267645	GC_Urine
1910	GCU_1910	GCU_245734	GC_Urine
1911	GCU_1911	GCU_245705	GC_Urine
1912	GCU_1912	GCU_244467	GC_Urine
1913	GCU_1913	GCU_242565	GC_Urine
1914	GCU_1914	GCU_242526	GC_Urine
1915	GCU_1915	GCU_242419	GC_Urine
1916	GCU_1916	GCU_241911	GC_Urine
1917	GCU_1917	GCU_241882	GC_Urine
1918	GCU_1918	GCU_241387	GC_Urine
1919	GCU_1919	GCU_241319	GC_Urine
1920	GCU_1920	GCU_241312	GC_Urine
1921	GCU_1921	GCU_241307	GC_Urine
1922	GCU_1922	GCU_241277	GC_Urine
1923	GCU_1923	GCU_241218	GC_Urine
1924	GCU_1924	GCU_241189	GC_Urine
1925	GCU_1925	GCU_241141	GC_Urine
1926	GCU_1926	GCU_241097	GC_Urine
1927	GCU_1927	GCU_241095	GC_Urine
1928	GCU_1928	GCU_241054	GC_Urine
1929	GCU_1929	GCU_241039	GC_Urine
1930	GCU_1930	GCU_241032	GC_Urine
1931	GCU_1931	GCU_240984	GC_Urine

1932	GCU_1932	GCU_240583	GC_Urine
1933	GCU_1933	GCU_240374	GC_Urine
1934	GCU_1934	GCU_240346	GC_Urine
1935	GCU_1935	GCU_239565	GC_Urine
1936	GCU_1936	GCU_239312	GC_Urine
1937	GCU_1937	GCU_238571	GC_Urine
1938	GCU_1938	GCU_238291	GC_Urine
1939	GCU_1939	GCU_238267 trisaccharide	GC_Urine
1940	GCU_1940	GCU_238165	GC_Urine
1941	GCU_1941	GCU_237946	GC_Urine
1942	GCU_1942	GCU_237937	GC_Urine
1943	GCU_1943	GCU_237912	GC_Urine
1944	GCU_1944	GCU_237892	GC_Urine
1945	GCU_1945	GCU_237772	GC_Urine
1946	GCU_1946	GCU_237520	GC_Urine
1947	GCU_1947	GCU_237488	GC_Urine
1948	GCU_1948	GCU_237358	GC_Urine
1949	GCU_1949	GCU_237281	GC_Urine
1950	GCU_1950	GCU_237267	GC_Urine
1951	GCU_1951	GCU_236889	GC_Urine
1952	GCU_1952	GCU_236870	GC_Urine
1953	GCU_1953	GCU_236810	GC_Urine
1954	GCU_1954	GCU_236725	GC_Urine
1955	GCU_1955	GCU_236724	GC_Urine
1956	GCU_1956	GCU_235770	GC_Urine
1957	GCU_1957	GCU_235755	GC_Urine
1958	GCU_1958	GCU_234636	GC_Urine
1959	GCU_1959	GCU_234618	GC_Urine
1960	GCU_1960	GCU_234486	GC_Urine
1961	GCU_1961	GCU_234432	GC_Urine
1962	GCU_1962	GCU_233803	GC_Urine
1963	GCU_1963	GCU_233455	GC_Urine
1964	GCU_1964	GCU_233443	GC_Urine
1965	GCU_1965	GCU_233429	GC_Urine
1966	GCU_1966	GCU_233331	GC_Urine
1967	GCU_1967	GCU_233289	GC_Urine
1968	GCU_1968	GCU_233275	GC_Urine
1969	GCU_1969	GCU_233017	GC_Urine
1970	GCU_1970	GCU_232946	GC_Urine
1971	GCU_1971	GCU_232869	GC_Urine
1972	GCU_1972	GCU_232774	GC_Urine
1973	GCU_1973	GCU_232752	GC_Urine
1974	GCU_1974	GCU_232739	GC_Urine
1975	GCU_1975	GCU_232707	GC_Urine
1976	GCU_1976	GCU_232660	GC_Urine
1977	GCU_1977	GCU_232659	GC_Urine
1978	GCU_1978	GCU_232658	GC_Urine
1979	GCU_1979	GCU_232610	GC_Urine
1980	GCU_1980	GCU_232586	GC_Urine
1981	GCU_1981	GCU_232506	GC_Urine
1982	GCU_1982	GCU_232366	GC_Urine
1983	GCU_1983	GCU_232185	GC_Urine
1984	GCU_1984	GCU_232102	GC_Urine
1985	GCU_1985	GCU_232075	GC_Urine
1986	GCU_1986	GCU_231947	GC_Urine
1987	GCU_1987	GCU_231945	GC_Urine
1988	GCU_1988	GCU_231941	GC_Urine
1989	GCU_1989	GCU_231935	GC_Urine
1990	GCU_1990	GCU_231879	GC_Urine
1991	GCU_1991	GCU_231813	GC_Urine
1992	GCU_1992	GCU_231805	GC_Urine
1993	GCU_1993	GCU_231801	GC_Urine

1994	GCU_1994	GCU_231796	GC_Urine
1995	GCU_1995	GCU_231708	GC_Urine
1996	GCU_1996	GCU_231685	GC_Urine
1997	GCU_1997	GCU_231680	GC_Urine
1998	GCU_1998	GCU_231678	GC_Urine
1999	GCU_1999	GCU_231657	GC_Urine
2000	GCU_2000	GCU_231544	GC_Urine
2001	GCU_2001	GCU_231511	GC_Urine
2002	GCU_2002	GCU_231248	GC_Urine
2003	GCU_2003	GCU_231210	GC_Urine
2004	GCU_2004	GCU_231123	GC_Urine
2005	GCU_2005	GCU_231070	GC_Urine
2006	GCU_2006	GCU_229947	GC_Urine
2007	GCU_2007	GCU_229199	GC_Urine
2008	GCU_2008	GCU_229010	GC_Urine
2009	GCU_2009	GCU_228918	GC_Urine
2010	GCU_2010	GCU_228913	GC_Urine
2011	GCU_2011	GCU_228911	GC_Urine
2012	GCU_2012	GCU_228904	GC_Urine
2013	GCU_2013	GCU_228630	GC_Urine
2014	GCU_2014	GCU_228613	GC_Urine
2015	GCU_2015	GCU_228546	GC_Urine
2016	GCU_2016	GCU_228405	GC_Urine
2017	GCU_2017	GCU_228394	GC_Urine
2018	GCU_2018	GCU_228262	GC_Urine
2019	GCU_2019	GCU_228249	GC_Urine
2020	GCU_2020	GCU_228202	GC_Urine
2021	GCU_2021	GCU_228075	GC_Urine
2022	GCU_2022	GCU_228070	GC_Urine
2023	GCU_2023	GCU_228039	GC_Urine
2024	GCU_2024	GCU_228008	GC_Urine
2025	GCU_2025	GCU_228004	GC_Urine
2026	GCU_2026	GCU_227902	GC_Urine
2027	GCU_2027	GCU_227774	GC_Urine
2028	GCU_2028	GCU_227769	GC_Urine
2029	GCU_2029	GCU_227716	GC_Urine
2030	GCU_2030	GCU_227710	GC_Urine
2031	GCU_2031	GCU_227677	GC_Urine
2032	GCU_2032	GCU_227656	GC_Urine
2033	GCU_2033	GCU_227632	GC_Urine
2034	GCU_2034	GCU_227626	GC_Urine
2035	GCU_2035	GCU_227604	GC_Urine
2036	GCU_2036	GCU_227601	GC_Urine
2037	GCU_2037	GCU_227592	GC_Urine
2038	GCU_2038	GCU_227588	GC_Urine
2039	GCU_2039	GCU_227367	GC_Urine
2040	GCU_2040	GCU_227360	GC_Urine
2041	GCU_2041	GCU_226303	GC_Urine
2042	GCU_2042	GCU_225891	GC_Urine
2043	GCU_2043	GCU_225890	GC_Urine
2044	GCU_2044	GCU_225882	GC_Urine
2045	GCU_2045	GCU_225867	GC_Urine
2046	GCU_2046	GCU_225863	GC_Urine
2047	GCU_2047	GCU_225854	GC_Urine
2048	GCU_2048	GCU_225494	GC_Urine
2049	GCU_2049	GCU_225481	GC_Urine
2050	GCU_2050	GCU_225417	GC_Urine
2051	GCU_2051	GCU_225262	GC_Urine
2052	GCU_2052	GCU_225043	GC_Urine
2053	GCU_2053	GCU_224901	GC_Urine
2054	GCU_2054	GCU_224843	GC_Urine
2055	GCU_2055	GCU_224799	GC_Urine

2056	GCU_2056	GCU_224633	GC_Urine
2057	GCU_2057	GCU_224533	GC_Urine
2058	GCU_2058	GCU_224322	GC_Urine
2059	GCU_2059	GCU_223865	GC_Urine
2060	GCU_2060	GCU_223854	GC_Urine
2061	GCU_2061	GCU_223625	GC_Urine
2062	GCU_2062	GCU_223618	GC_Urine
2063	GCU_2063	GCU_223535	GC_Urine
2064	GCU_2064	GCU_222169	GC_Urine
2065	GCU_2065	GCU_222115	GC_Urine
2066	GCU_2066	GCU_222050	GC_Urine
2067	GCU_2067	GCU_221701	GC_Urine
2068	GCU_2068	GCU_221594	GC_Urine
2069	GCU_2069	GCU_221571	GC_Urine
2070	GCU_2070	GCU_221568	GC_Urine
2071	GCU_2071	GCU_221505	GC_Urine
2072	GCU_2072	GCU_220384	GC_Urine
2073	GCU_2073	GCU_220136	GC_Urine
2074	GCU_2074	GCU_220122	GC_Urine
2075	GCU_2075	GCU_220007	GC_Urine
2076	GCU_2076	GCU_219510	GC_Urine
2077	GCU_2077	GCU_219486	GC_Urine
2078	GCU_2078	GCU_218821	GC_Urine
2079	GCU_2079	GCU_218816	GC_Urine
2080	GCU_2080	GCU_218767	GC_Urine
2081	GCU_2081	GCU_218765	GC_Urine
2082	GCU_2082	GCU_218724	GC_Urine
2083	GCU_2083	GCU_218708	GC_Urine
2084	GCU_2084	GCU_218699	GC_Urine
2085	GCU_2085	GCU_218694	GC_Urine
2086	GCU_2086	GCU_218574	GC_Urine
2087	GCU_2087	GCU_218520	GC_Urine
2088	GCU_2088	GCU_217893	GC_Urine
2089	GCU_2089	GCU_217816	GC_Urine
2090	GCU_2090	GCU_217783	GC_Urine
2091	GCU_2091	GCU_216870	GC_Urine
2092	GCU_2092	GCU_216844	GC_Urine
2093	GCU_2093	GCU_216838	GC_Urine
2094	GCU_2094	GCU_216427	GC_Urine
2095	GCU_2095	GCU_215559	GC_Urine
2096	GCU_2096	GCU_215532	GC_Urine
2097	GCU_2097	GCU_215520	GC_Urine
2098	GCU_2098	GCU_215494	GC_Urine
2099	GCU_2099	GCU_215397	GC_Urine
2100	GCU_2100	GCU_215345	GC_Urine
2101	GCU_2101	GCU_215066	GC_Urine
2102	GCU_2102	GCU_214685	GC_Urine
2103	GCU_2103	GCU_214405	GC_Urine
2104	GCU_2104	GCU_214313	GC_Urine
2105	GCU_2105	GCU_214287	GC_Urine
2106	GCU_2106	GCU_214175	GC_Urine
2107	GCU_2107	GCU_214172	GC_Urine
2108	GCU_2108	GCU_214166	GC_Urine
2109	GCU_2109	GCU_214158	GC_Urine
2110	GCU_2110	GCU_214011	GC_Urine
2111	GCU_2111	GCU_213972	GC_Urine
2112	GCU_2112	GCU_213735	GC_Urine
2113	GCU_2113	GCU_213353	GC_Urine
2114	GCU_2114	GCU_213296	GC_Urine
2115	GCU_2115	GCU_213253	GC_Urine
2116	GCU_2116	GCU_213155	GC_Urine
2117	GCU_2117	GCU_212781	GC_Urine

2118	GCU_2118	GCU_212748	GC_Urine
2119	GCU_2119	GCU_212734	GC_Urine
2120	GCU_2120	GCU_212586	GC_Urine
2121	GCU_2121	GCU_212209	GC_Urine
2122	GCU_2122	GCU_211991	GC_Urine
2123	GCU_2123	GCU_211916	GC_Urine
2124	GCU_2124	GCU_211902	GC_Urine
2125	GCU_2125	GCU_211896	GC_Urine
2126	GCU_2126	GCU_211891	GC_Urine
2127	GCU_2127	GCU_211736	GC_Urine
2128	GCU_2128	GCU_211378	GC_Urine
2129	GCU_2129	GCU_210286	GC_Urine
2130	GCU_2130	GCU_208770	GC_Urine
2131	GCU_2131	GCU_208723	GC_Urine
2132	GCU_2132	GCU_208712	GC_Urine
2133	GCU_2133	GCU_208688	GC_Urine
2134	GCU_2134	GCU_208655	GC_Urine
2135	GCU_2135	GCU_208647	GC_Urine
2136	GCU_2136	GCU_208397	GC_Urine
2137	GCU_2137	GCU_208394	GC_Urine
2138	GCU_2138	GCU_207598	GC_Urine
2139	GCU_2139	GCU_207223	GC_Urine
2140	GCU_2140	GCU_206526	GC_Urine
2141	GCU_2141	GCU_206309	GC_Urine
2142	GCU_2142	GCU_206289	GC_Urine
2143	GCU_2143	GCU_205849	GC_Urine
2144	GCU_2144	GCU_205680	GC_Urine
2145	GCU_2145	GCU_205679	GC_Urine
2146	GCU_2146	GCU_205675	GC_Urine
2147	GCU_2147	GCU_205670	GC_Urine
2148	GCU_2148	GCU_205669	GC_Urine
2149	GCU_2149	GCU_204994	GC_Urine
2150	GCU_2150	GCU_204426	GC_Urine
2151	GCU_2151	GCU_204425	GC_Urine
2152	GCU_2152	GCU_204375	GC_Urine
2153	GCU_2153	GCU_204344	GC_Urine
2154	GCU_2154	GCU_204283	GC_Urine
2155	GCU_2155	GCU_204217	GC_Urine
2156	GCU_2156	GCU_203786	GC_Urine
2157	GCU_2157	GCU_203765	GC_Urine
2158	GCU_2158	GCU_203592	GC_Urine
2159	GCU_2159	GCU_203235	GC_Urine
2160	GCU_2160	GCU_202911	GC_Urine
2161	GCU_2161	GCU_202899	GC_Urine
2162	GCU_2162	GCU_202857	GC_Urine
2163	GCU_2163	GCU_202832	GC_Urine
2164	GCU_2164	GCU_202737	GC_Urine
2165	GCU_2165	GCU_202681	GC_Urine
2166	GCU_2166	GCU_202571	GC_Urine
2167	GCU_2167	GCU_202361	GC_Urine
2168	GCU_2168	GCU_202250	GC_Urine
2169	GCU_2169	GCU_202196	GC_Urine
2170	GCU_2170	GCU_202091	GC_Urine
2171	GCU_2171	GCU_202081	GC_Urine
2172	GCU_2172	GCU_202056	GC_Urine
2173	GCU_2173	GCU_201984	GC_Urine
2174	GCU_2174	GCU_201902	GC_Urine
2175	GCU_2175	GCU_201887	GC_Urine
2176	GCU_2176	GCU_201870	GC_Urine
2177	GCU_2177	GCU_201862	GC_Urine
2178	GCU_2178	GCU_201042	GC_Urine
2179	GCU_2179	GCU_200909	GC_Urine

2180	GCU_2180	GCU_200905	GC_Urine
2181	GCU_2181	GCU_200712	GC_Urine
2182	GCU_2182	GCU_200687	GC_Urine
2183	GCU_2183	GCU_200675	GC_Urine
2184	GCU_2184	GCU_200670	GC_Urine
2185	GCU_2185	GCU_200629	GC_Urine
2186	GCU_2186	GCU_200597	GC_Urine
2187	GCU_2187	GCU_200589	GC_Urine
2188	GCU_2188	GCU_200588	GC_Urine
2189	GCU_2189	GCU_200541	GC_Urine
2190	GCU_2190	GCU_200540	GC_Urine
2191	GCU_2191	GCU_200506	GC_Urine
2192	GCU_2192	GCU_200490	GC_Urine
2193	GCU_2193	GCU_200478	GC_Urine
2194	GCU_2194	GCU_200472	GC_Urine
2195	GCU_2195	GCU_200469	GC_Urine
2196	GCU_2196	GCU_200468	GC_Urine
2197	GCU_2197	GCU_200421	GC_Urine
2198	GCU_2198	GCU_200392	GC_Urine
2199	GCU_2199	GCU_200391	GC_Urine
2200	GCU_2200	GCU_1-methyl-1,3-propanediyl)bis(oxy)-2TMS NIST	GC_Urine
2201	GCU_2201	GCU_199596	GC_Urine
2202	GCU_2202	GCU_199562	GC_Urine
2203	GCU_2203	GCU_199553	GC_Urine
2204	GCU_2204	GCU_199463	GC_Urine
2205	GCU_2205	GCU_199433	GC_Urine
2206	GCU_2206	GCU_199338	GC_Urine
2207	GCU_2207	GCU_199337	GC_Urine
2208	GCU_2208	GCU_199317	GC_Urine
2209	GCU_2209	GCU_199229	GC_Urine
2210	GCU_2210	GCU_199177	GC_Urine
2211	GCU_2211	GCU_492765	GC_Urine
2212	GCU_2212	GCU_487513	GC_Urine
2213	GCU_2213	GCU_487272	GC_Urine
2214	GCU_2214	GCU_487269	GC_Urine
2215	GCU_2215	GCU_486245	GC_Urine
2216	GCU_2216	GCU_486213	GC_Urine
2217	GCU_2217	GCU_486211	GC_Urine
2218	GCU_2218	GCU_486054	GC_Urine
2219	GCU_2219	GCU_486020	GC_Urine
2220	GCU_2220	GCU_486017	GC_Urine
2221	GCU_2221	GCU_486007	GC_Urine
2222	GCU_2222	GCU_485553	GC_Urine
2223	GCU_2223	GCU_485481	GC_Urine
2224	GCU_2224	GCU_485464	GC_Urine
2225	GCU_2225	GCU_485443	GC_Urine
2226	GCU_2226	GCU_485397	GC_Urine
2227	GCU_2227	GCU_485388	GC_Urine
2228	GCU_2228	GCU_484792	GC_Urine
2229	GCU_2229	GCU_484719	GC_Urine
2230	GCU_2230	GCU_484689	GC_Urine
2231	GCU_2231	GCU_484573	GC_Urine
2232	GCU_2232	GCU_484345	GC_Urine
2233	GCU_2233	GCU_484307	GC_Urine
2234	GCU_2234	GCU_484108	GC_Urine
2235	GCU_2235	GCU_484104	GC_Urine
2236	GCU_2236	GCU_483593	GC_Urine
2237	GCU_2237	GCU_483487	GC_Urine
2238	GCU_2238	GCU_483342	GC_Urine
