# Supplementary material for: Modulation of the Metabiome by Rifaximin in Patients with Cirrhosis and Minimal Hepatic Encephalopathy
Source: PLoS One. 2013 Apr 2;8(4):e60042. doi: 10.1371/journal.pone.0060042 (PMC3615021; doi:10.1371/journal.pone.0060042)
Supplement: Text S1 — Includes Supplementary Methods (Pages S1–6), Supplementary Discussion (pages S7–9), Supplementary References (Page S10) and Supplementary Figure and Table Legends (page S11). (DOCX) [file pone.0060042.s004.docx]

**Supplementary Methods**

Supplementary microbiome analysis methods:

Stool (0.2 mg) was suspended in buffer argininosuccinate lyase to which 0.75-g 0.1-mm zirconia/silica beads (Biospec Products) were added, and cells were disrupted twice at 4,800 revolution/min on a bead beater for 1 minute. Tubes were placed in a 90°C water bath for 10 min, and the remainder of the protocol was followed according to the manufacturer. Microbiome community fingerprinting: We routinely use length heterogeneity PCR (LH-PCR) fingerprinting of the 16S rRNA to rapidly survey our samples and standardize the community amplification[1]. Briefly, total genomic DNA was extracted from tissue using Bio101 kit from MP Biomedicals per the manufacturer's instructions. About 10 ng of extracted DNA was amplified by PCR using a fluorescently labeled forward primer 27F [5′- (6FAM) AGAGTTTGATCCTGGCTCA G-3′] and unlabeled reverse primer 355R′ (5′-GCTGCCTCCCGTAGGAGT-3′). Both primers are universal primers for bacteria. The LH-PCR products were diluted according to their intensity on agarose gel electrophoresis and mixed with ILS-600 size standards (Promega) and HiDi Formamide (Applied Biosystems). The diluted samples were then separated on an ABI 3130xl fluorescent capillary sequencer (Applied Biosystems) and processed using the Genemapper software package (Applied Biosystems). Normalized peak areas were calculated using a custom PERL script, and operational taxonomic units constituting less than 1% of the total community from each sample were eliminated from the analysis to remove the variable low-abundance components within the communities.

Multitag Pyrosequencing (MTPS): We then interrogated the microbial taxa associated with the gut fecal microbiome using multitag pyrosequencing (MTPS). This technique allows the rapid sequencing of multiple samples at one time, yielding thousands of sequence reads per sample[1]. Specifically, we have generated a set of 96 emulsion PCR fusion primers that contain the 454 emulsion PCR linkers on the 27F and 355R primers and different eight-base “barcode” between the A adapter and 27F primer. Thus each fecal sample was amplified with unique barcoded forward 16S rRNA primers, and then up to 96 samples were pooled and subjected to emulsion PCR and pyrosequenced using a GS-Junior pyrosequencer (Roche). Data from each pooled sample were “deconvoluted” by sorting the sequences into bins based on the barcodes using custom PERL scripts. Reads were filtered based on quality scores and length. Thus we were able to normalize each sample by the total number of reads from each barcode. We have noted that ligating tagged primers to PCR amplicons distorts the abundances of the communities, and thus it is critical to incorporate the tags during the original amplification step[1]. We identified the taxa present in each sample using the Bayesian analysis tool in Version 10 of the Ribosomal Database Project (RDP10). The abundances of the bacterial identifications were then normalized using a custom PERL script, and taxa present at >1% of the community were tabulated. We chose this cutoff because of our a priori assumption that taxa present in <1% of the community vary between individuals and have minimal contribution to the functionality of that community and that 2,000 reads per sample will only reliably identify community components that are >1% in abundance. . Unifrac analysis was performed using Version 1.3.0 of Quantitative Insights into Microbial Ecology (QIIME) and weighted P-values were calculated using a Bonferroni correction.

Supplementary Metabolomic methods:

GC MS Data acquisition: 30 ul serum, or 10 ul urine samples, were taken out one by one and extracted with concomitant protein precipitation by 1 ml of a mixture of nitrogen-degassed -20°C cold of isopropanol/acetonitrile/water (3:3:2, v/v/v) by shaking for 5 min at 4°C. After centrifugation at 12,800x *g* for 2 min, 90% of the supernatant was removed, separated into two equal aliquots, transferred to a 1.5 ml Eppendorf tube and concentrated to dryness in a vacuum concentrator. Plasma samples (but not urine samples) were purified by adding 500 ul of a nitrogen-degassed acetonitrile/water mixture (1/1, v/v), vortexing, centrifugation, decanting the supernatant and drying in a speed vac concentrator. This step removed plasma triglycerides and most of the complex lipids found in blood, but not phytosterols or free fatty acids. This step is needed because otherwise, the involatile matrix lipids would interfere with the derivatization reaction of primary amines and amino acids. C08-C30 fatty acid methyl esters in chloroform were added as internal retention index (RI) markers. Subsequently, metabolites were derivatized by adding 10 ul of a solution of 20 mg/ml of 98% pure methoxyamine hydrochloride (CAS number 593–56-6, Sigma-Aldrich) in pyridine for 90 min at 28°C. Afterwards 90 ul of N-methyl-N-trimethylsilyltrifluoroacetamide (MSTFA, Sigma-Aldrich) was added for trimethylsilylation of acidic protons and shaken at 37°C for 30 min. The reaction mixture was transferred to a 2 ml clear glass auto-sampler vial with micro-insert (Agilent) and closed using a 11 mm T/S/T crimp cap (MicroLiter). A Gerstel automatic liner exchange system with a MPS2 dual rail multi-purpose sampler was used in conjunction with a Gerstel CIS cold injection system. For every 10 samples, a fresh multi-baffled liner was inserted (Gerstel #011711-010-00). Before and after each injection, the 10 ul injection syringe was washed three times with 10 ul ethyl acetate. 1 ul sample was filled using 39 mm vial penetration at 1ul sec^-1^ filling speed, injecting 0.5 ul at a 10 ul s^-1^ injection speed at an initial temperature of 50°C which was ramped by 12°C sec^-1^ to a final temperature of 250°C and held for 3 min. The injector was operated in split-less mode, opening the split vent after 25 sec. Samples were injected between 2–24 h after derivatization using randomized sequences controlled by the laboratory information management and database system. An Agilent 6890 gas chromatograph was used with a 30 m long, 0.25 mm internal diameter Rtx-5Sil MS column with 0.25 lm 95% dimethyl/5%diphenyl polysiloxane film and an additional 10 m integrated guard column was used (Restek). Separation was achieved by 99.9999% pure helium with built-in purifier (Airgas) at a constant flow of 1 ml min^-1^. The oven temperature was held constant at 50°C for 1 min, and then ramped at 20°C min^-1^ to 330°C, and held constant for 5 min. The transfer line temperature was set to 280 °C. After 290 s solvent delay, filament 1 was turned on at an ion source temperature of 250 °C. Electron ionization mass spectra were acquired at +70 eV with mass resolving power R = 600 from m/z 85-500 at 20 spectra s^-1^ and 1850 V detector voltage without turning on the mass defect option. Recording ended after 1200 s. The instrument performed auto-tuning for mass calibration using FC43 (perfluorotributyl-amine) before starting analysis sequences.

Metabolome Data processing: Chromatogram acquisition, data handling, automated peak deconvolution, and export of spectra was automatically performed by the Leco ChromaTOF software (v2.32). Peak picking was achieved in ChromaTOF (v2.32) at signal/noise levels of 10:1 throughout the chromatogram with baseline subtraction just above the noise level, no smoothing, 3 s default peak widths, automatic mass spectral deconvolution and peak detection and export of result spectra as *.csv files in addition to export of raw data in open-access *.cdf formats. Data were further processed using the algorithms implemented in the open-source BinBase metabolome database. This algorithm used the settings: validity of chromatogram (<10 peaks with intensity >10^7 counts sec^-1^), unbiased retention index marker detection (MS similarity >800 and exceeding thresholds for ion ratio abundances for high m/z marker ions), retention index calculation by 5th order polynomial regression. Spectra were cut to 5% base peak abundance, and matched to database entries from most- to least-abundant spectra using the following matching filters: retention index window ±2000 units (equivalent to about ±2 sec retention time), validation of unique ions and apex masses (unique ion must be included in apex masses and present at >3% of base peak abundance), mass spectrum similarity that must fit criteria dependent on peak purity and signal/noise ratios, optional ion ratio settings to distinguish peaks with high similarity, and a final isomer filter (annotating the isomer spectrum with the closest RI fit). Novel spectra not yet included in BinBase were automatically entered as new database entries if their signal-to-noise ratio >25, purity <1.0 and presence in the biological study design class was >80%. This filter ensured that (i) signals were reported that had never been detected previously in any other sample, but (ii) only signals were reported that can be assumed to be biologically relevant using relatively abundant and pure signals and ensuring that these are positively detected in most of the biological replicates. Signal intensities were reported as peak heights using the unique ion as default, unless an alternative quantification ion was manually set in the BinBase administration software Bellerophon. All known artifact peaks such as internal standards, column bleed, plasticizers or reagent peaks were assigned by BinBase but not exported for further statistical calculations. Metabolites were identified using the Fiehnlib libraries consisting of over 1,000 authentic compounds and referenced using PubChem identifiers. Daily quality controls were used comprising method blanks and five-point calibration curve samples of 31 pure reference compounds which were repeatedly analyzed over the full analytical sequence in addition to injection of one QC sample for every 10 biological samples. A quantification report table was produced for all database entries that were positively detected in more than 50% of the samples of a study design class (as defined in the in-house miniX database). This procedure results in 10–30% missing values, which could be caused by true negatives (compounds that were below the detection limit in a specific sample) or false negatives (compounds that were present in a specific sample but that did not match quality criteria in the BinBase algorithm). A subsequent post-processing module was employed to automatically replace missing values from the *.cdf files with the following parameters: for each positively detected metabolite, the average retention time was calculated for the day of analysis. Subsequently, for each chromatogram and each missing value, the intensity of the quantification ion at this retention time was extracted by seeking its maximum value in a retention time region of ± 1 s and subtracting the minimum (local background) intensity in a retention time region of ±5 s around the peak maximum. The resulting report table therefore did not have any missing values. Replaced values were labeled as ‘lower confidence’ by color coding.

Metabolome Data normalization and statistics: Result files were normalized by calculating the sum intensities of all structurally identified compounds for each sample (i.e. those signals that had been positively identified in the data pre-processing schema outlined above), and subsequently dividing all data associated with a sample by the corresponding metabolite sum. The resulting data were multiplied by the average sum of all identified metabolites detected in the study (total average metabolome transformation), disregarding unknown metabolites as these might potentially also comprise artifacts. Intensities of identified metabolites with more than one peak (e.g. for the syn- and anti-forms of methoximated reducing sugars, or amino acids with different derivatization status of amine groups) were summed to only one value in the transformed data set. The original non-transformed data set was retained for retrospective analysis. When comparing classes of samples with biologically different sum concentrations of identified metabolites (*p*<0.05), these class averages were used for mean transformations.

**Supplementary Discussion of Bacterial Correlation Networks:**

*Bacteroidaceae* correlations (Supplementary Figure 3): Prior to rifaximin therapy, a positive correlation between *Bacteroidaceae* and metabolites associated with ammonia generation (glutamic acid, glutamine, asparagine), and oxidative stress (parabanic acid) was seen[2]. *Bacteriodaceae* were negatively linked with products of ammonia fixation and nitrification (hydroxylamine), with pantothenic acid, that is needed for acetyl coenzyme-A generation and ammonia fixation through carbamoyl phosphate synthetase I and the mitochondrial shuttle and urine alanine which is a non-toxic route of nitrogen excretion[3]. There was also a negative correlation with dopamine, which is an active neuro-transmitter synthesized by phenylalanine and tyrosine, that is often deficient in cirrhotic brains[4]. Also as expected there was a significant negative correlation directly as well through several unnamed compounds (most have been removed for clarity) with the autochthonous *Lachnospiraceae* and Clostridium Cluster XIV. Interestingly the correlations of *Bacteriodaceae* changed dramatically after rifaximin therapy. Post-rifaximin there was a negative correlation with *Enterobacteriaceae* abundance and indirectly negatively with serum ammonia. We also found an interesting negative linkage with acetophenone that serves as a quorum sensor in *Enterobacteriaceae*; this could modulate the *Enterobacteriaceae* abundance[5]. There was also a negative correlation with carbohydrate metabolism intermediates (galactinol, which are normally metabolized into raffinose and broken down by gut bacteria), malic acid (Kreb’s cycle intermediate) and urinary valine. *Bacteroidaceae* before were negatively correlated with autochthonous families (Clostridium cluster XIV and *Lachnospiraceae*) directly and with *Ruminococcaceae* indirectly. After rifaximin they were negatively linked with *Enterobacteriaceae* and in the same direction as *Porphyromonadaceae*.

*Porphyromonadaceae* correlations (Supplementary Figure 4): This taxon has been associated with poor cognitive performance in cirrhosis and with fatty liver disease[6,7]. Before rifaximin it was negatively associated with *Rikenellaceae* and several of the fatty acids that ultimately increased as a result of rifaximin therapy. There was also a linkage with endotoxin through an unassigned metabolite. There was also a significant correlation with hippuric acid, an end-product of ammonia metabolism, which remained after rifaximin therapy. After rifaximin, *Porphyromonadaceae* were negatively correlated with butane 2,3 diol and hydroxybutanoic acids, which can both influence the co-existence of other gut bacteria, specifically members of *Enterobacteriaceae*[8]. After rifaximin there was also a negative correlation with serine and threonine which are sources of ammonia[9].

*Enterobacteriaceae* (Supplementary Figure 5) linkages significantly changed before and after rifaximin. The correlations were positive with aromatic amino acids and their metabolites (tryptophan, 5-methoxy tryptamine) and bacterial metabolites of glycerol (glycerol phosphate and propane 1,3 diol). After rifaximin, *Enterobacteriaceae* correlations changed and were correlated significantly with fatty acids that increased after rifaximin, negatively with *Ruminococcaceae* and negatively with 3-hydroxy butanoic acid and benzyl alcohol which can act as a quorum sensor for *Enterobacteriaceae* members[10]. Hydroxybutanoic acids are ketone bodies that are also markers of oxidative stress[11]. There were also positive correlations with carnitine derivatives that are needed for neurotransmission and ammonia transport.

*Veillonellaceae* correlations (Supplementary Figure 6): Pre-rifaximin, *Veillonellaceae* were correlated with tryptophan metabolites (3 aryl carbonyl alanine) and 2 hydroxy butanoic acid (ketone body and marker of oxidative stress), and with ammonia through an unnamed compound[8]. After rifaximin therapy, in addition to an absolute reduction in the abundance, there was a positive correlation with sophorose, the sugar moiety of the antibacterial and anti-endotoxin compound sophorolipid, with fatty acid metabolites (1 hexadecanol and valerylglycine) while a negative correlation with the tryptophan metabolite phenylacetic acid[12].

Autochthonous bacteria and *Rikenellaceae* correlations (Supplementary Figures 3 and 7): *Lachnospiraceae* and Clostridium cluster XIV remained positively correlated either directly or through metabolites in the same direction. *Ruminococcaceae* after was positively correlated with Clostridium cluster XIV but before were not correlated with them; before they were negatively correlated with *Bacteriodaceae*. Autochthonous bacterial families continued to have linkages before and after rifaximin with metabolites that reflect their overall beneficial nature. Specifically the families *Lachnospiraceae*, Clostridium cluster XIV and *Ruminococcaceae* were negatively linked with products of aromatic amino acid metabolism, ethanolamines and carboxylic acids were positively correlated with tocopherol alpha (vitamin E), urea and with the neuro-excitatory metabolite, aminoadipic acid[13]. *Lachnospiraceae* were also positively correlated with aminomalonic acid that is an inhibitor of asparagine synthetase. Inosine is a scavenger of peroxynitrite which prevents oxidative stress; it was positively correlated with the autochthonous family *Ruminococcaceae* and after rifaximin with *Rikenellaceae[14]*. *Rikenellaceae* was linked with *Porphyromonadaceae* prior to rifaximin through fatty acids but this correlation disappeared after rifaximin and was replaced by a dramatic positive correlation with autochthonous families, *Ruminococcaceae* and *Lachnospiraceae*.

**Supplementary Discussion References:**

1. Gillevet P, Sikaroodi M, Keshavarzian A, Mutlu EA (2010) Quantitative assessment of the human gut microbiome using multitag pyrosequencing. Chem Biodivers 7: 1065-1075.

2. Hillered L, Persson L (1995) Parabanic acid for monitoring of oxygen radical activity in the injured human brain. Neuroreport 6: 1816-1820.

3. Otsuka M, Akiba T, Okita Y, Tomita K, Yoshiyama N, et al. (1990) Lactic acidosis with hypoglycemia and hyperammonemia observed in two uremic patients during calcium hopantenate treatment. Jpn J Med 29: 324-328.

4. Skowronska M, Albrecht J Alterations of blood brain barrier function in hyperammonemia: an overview. Neurotox Res 21: 236-244.

5. Kesarwani M, Hazan R, He J, Que YA, Apidianakis Y, et al. A quorum sensing regulated small volatile molecule reduces acute virulence and promotes chronic infection phenotypes. PLoS Pathog 7: e1002192.

6. Bajaj JS, Ridlon JM, Hylemon PB, Thacker LR, Heuman DM, et al. (2012) Linkage of gut microbiome with cognition in hepatic encephalopathy. Am J Physiol Gastrointest Liver Physiol 302: G168-175.

7. Henao-Mejia J, Elinav E, Jin C, Hao L, Mehal WZ, et al. (2012) Inflammasome-mediated dysbiosis regulates progression of NAFLD and obesity. Nature 482: 179-185.

8. Campbell AK, Naseem R, Wann K, Holland IB, Matthews SB (2007) Fermentation product butane 2,3-diol induces Ca2+ transients in E. coli through activation of lanthanum-sensitive Ca2+ channels. Cell Calcium 41: 97-106.

9. Nelson J, Qureshi IA, Vasudevan S (1992) [Mechanism of the effect of serine and threonine on ammoniagenesis and biosynthesis of orotate in the mouse]. Clin Invest Med 15: 113-121.

10. Christensen BB, Haagensen JA, Heydorn A, Molin S (2002) Metabolic commensalism and competition in a two-species microbial consortium. Appl Environ Microbiol 68: 2495-2502.

11. Landaas S, Pettersen JE (1975) Clinical conditions associated with urinary excretion of 2-hydroxybutyric acid. Scand J Clin Lab Invest 35: 259-266.

12. Bluth MH, Kandil E, Mueller CM, Shah V, Lin YY, et al. (2006) Sophorolipids block lethal effects of septic shock in rats in a cecal ligation and puncture model of experimental sepsis. Crit Care Med 34: 188-195.

13. Wu HQ, Ungerstedt U, Schwarcz R (1995) L-alpha-aminoadipic acid as a regulator of kynurenic acid production in the hippocampus: a microdialysis study in freely moving rats. Eur J Pharmacol 281: 55-61.

14. Gudkov SV, Shtarkman IN, Smirnova VS, Chernikov AV, Bruskov VI (2006) Guanosine and inosine display antioxidant activity, protect DNA in vitro from oxidative damage induced by reactive oxygen species, and serve as radioprotectors in mice. Radiat Res 165: 538-545.

**Supplementary Figure and Table Legends**

Figure S1 Unifrac PCO Analysis: no significant difference was seen before (red) and after rifaximin (blue) therapy with respect to microbiome

Figure S2 Legend : PLS-DA shows significant separation between before and after rifaximin on urine and serum metabolites.

S2A: Urine Partial Least Square analysis Discriminant Analysis (PLS-DA).

S2B Serum PLS-DA before and after rifaximin treatment.

Legends common to S3-S7:

The complex correlation network represented parameters that were linked with a correlation coefficient >0.6 (negative or positive) and with a p value <0.05. Red nodes represent bacterial taxa, green one the serum metabolites, yellow nodes indicate urinary metabolites while blue ones indicate clinical parameters. Red edges represent negative correlation between connected nodes and blue edges indicate positive correlations.

Figure S3 *Bacteroidaceae* correlations with metabolome before and after Rifaximin treatment.

Figure S4 *Porphyromonadaceae* correlations with metabolome before and after Rifaximin treatment.

Figure S5 *Enterobacteriaceae* correlations with metabolome before and after Rifaximin treatment.

Figure S6 *Veillonellaceae* correlations with metabolome before and after Rifaximin treatment.

Figure S7 Autochthonous bacteria and *Rikenellaceae* correlations with metabolome before and after Rifaximin treatment.

Table S8 Table of Phenome, Microbiome, and Metabolome features used in Correlation Analysis.
