# Supplementary material for: Modulation of the Metabiome by Rifaximin in Patients with Cirrhosis and Minimal Hepatic Encephalopathy
Source: PLoS One. 2013 Apr 2;8(4):e60042. doi: 10.1371/journal.pone.0060042 (PMC3615021; doi:10.1371/journal.pone.0060042)
Supplement: File S1 — Figures S3–S7 with individual correlation networks centered before and after rifaximin around Figure S3: Bacterioidaceae , Figure S4: Porphyromonadaceae , Figure S5: Enterobacteriaceae , Figure S6: Veillonellaceae , Figure S7: Rickenellaceae. (PDF) [file pone.0060042.s005.pdf]

Figure S3

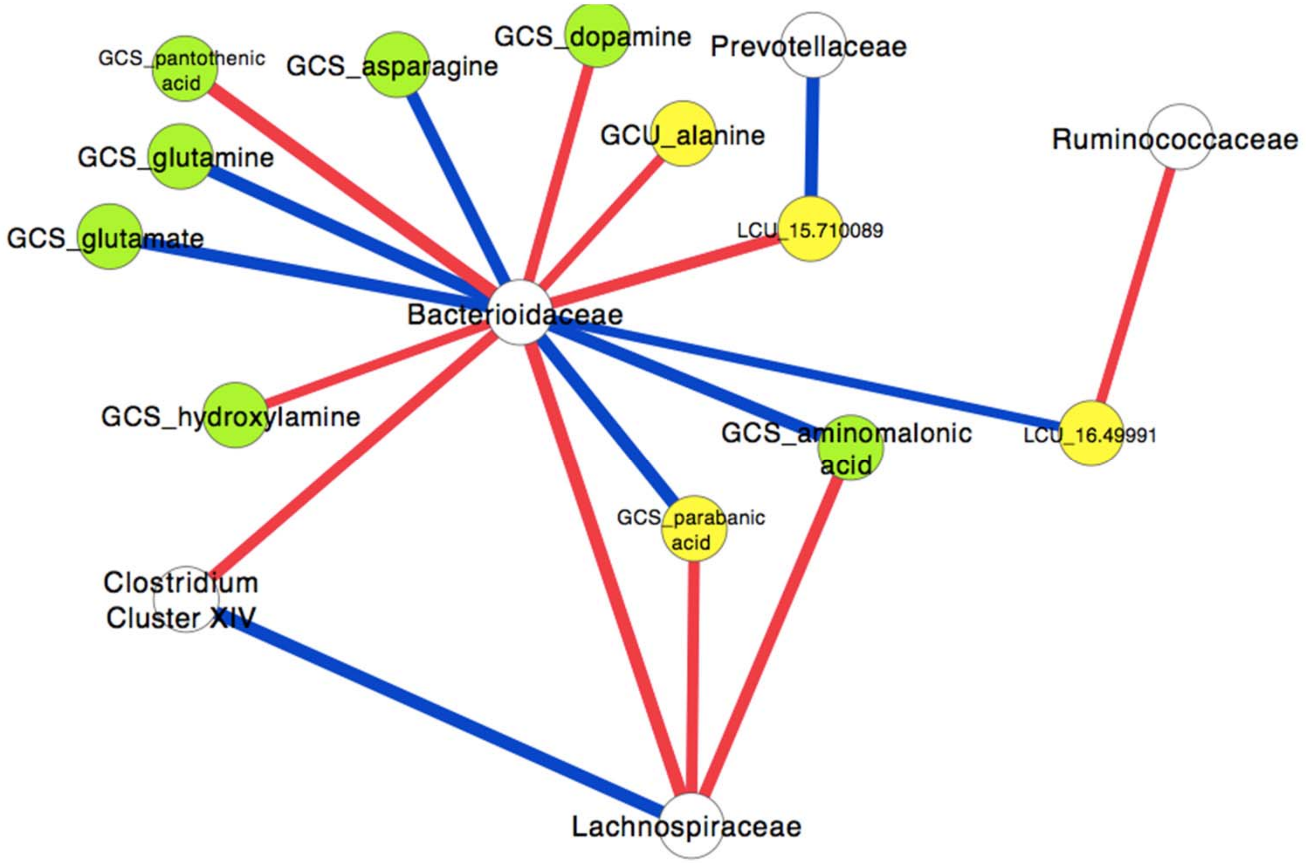

***Bacteriodacaeae* Before**

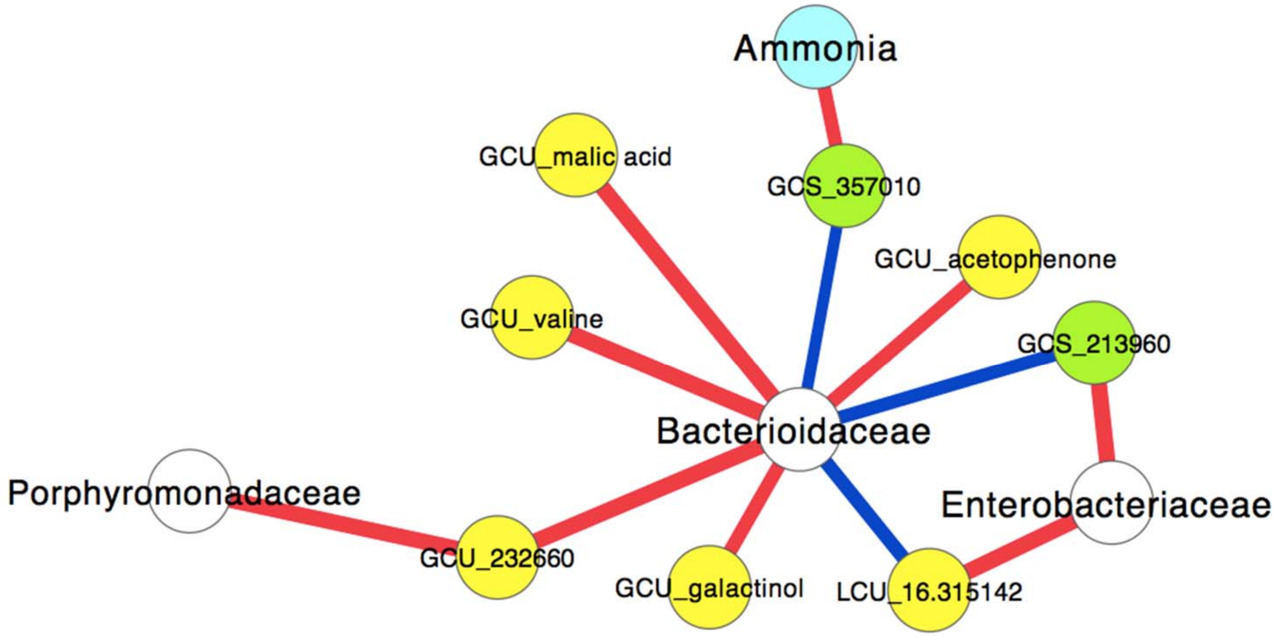

***Bacteriodacaeae* After**

Figure S4

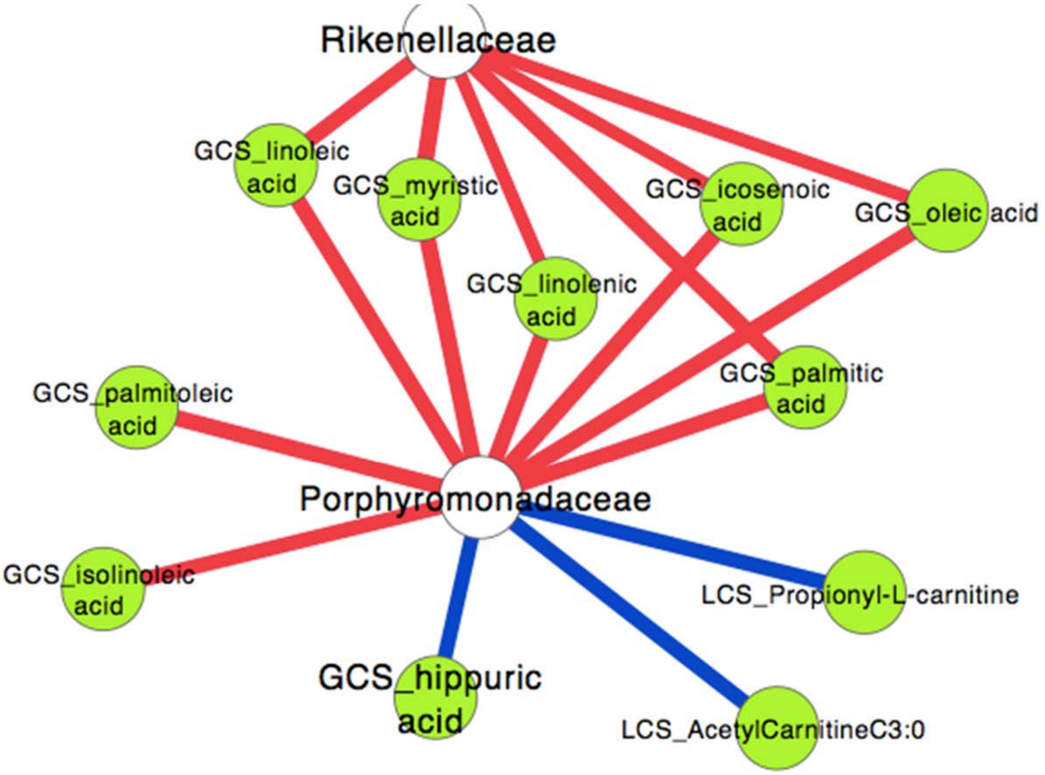

Porphyromonadaceae **Before**

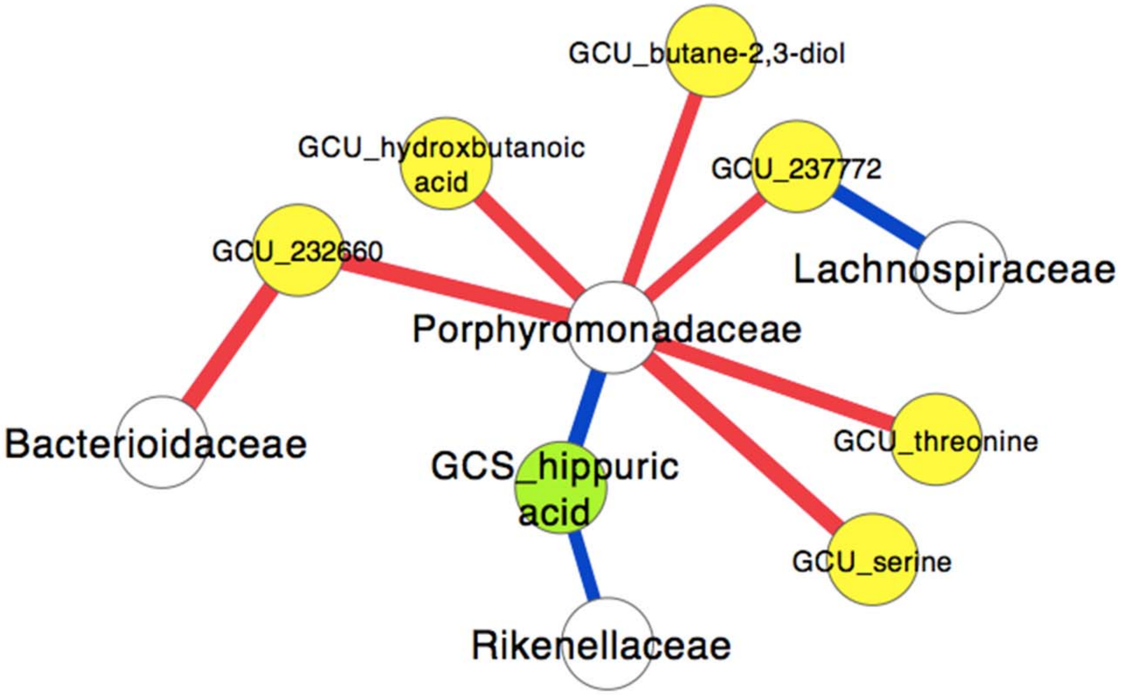

Porphyromonadaceae **After**

Figure S5

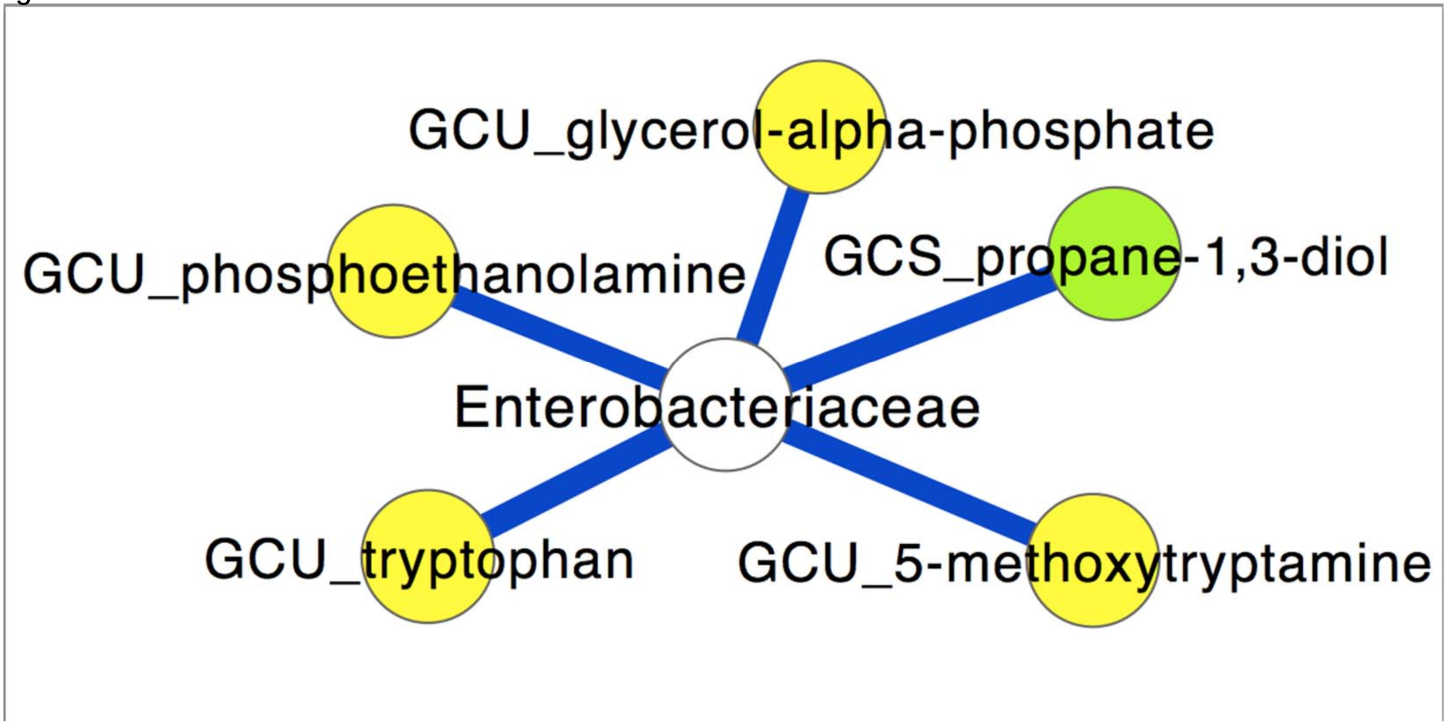

***Enterobacteriaceae* Before**

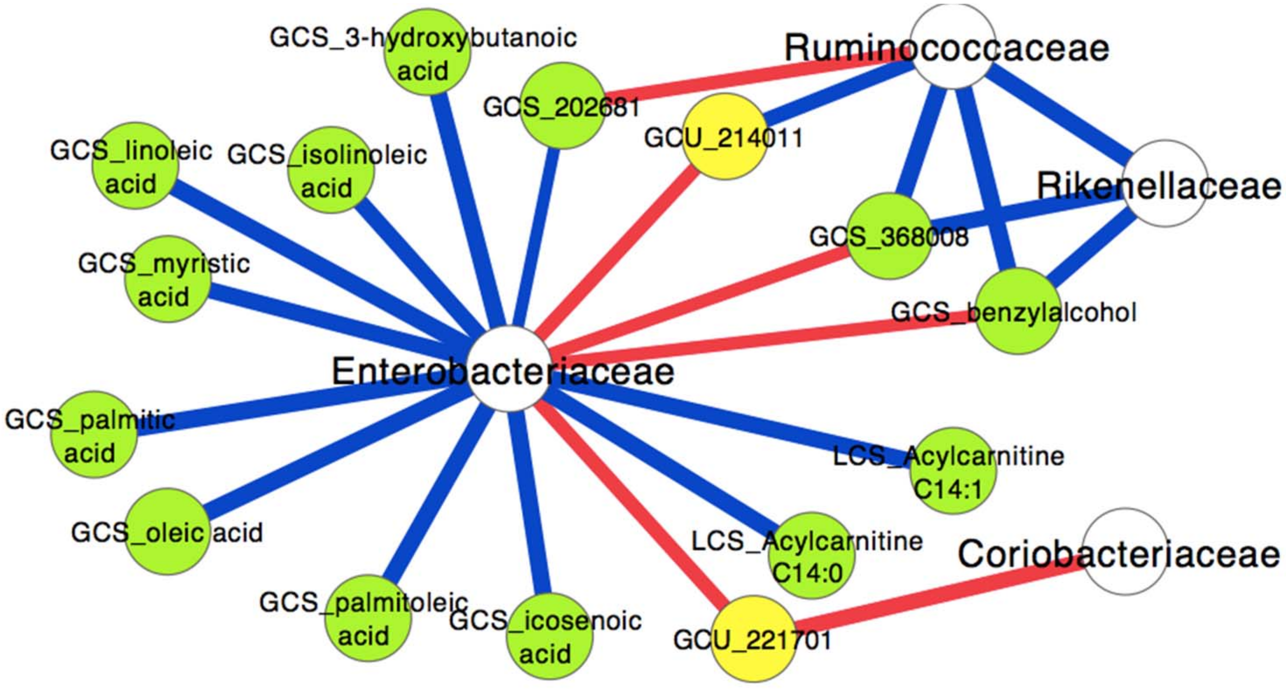

***Enterobacteriaceae* After**

Figure S6

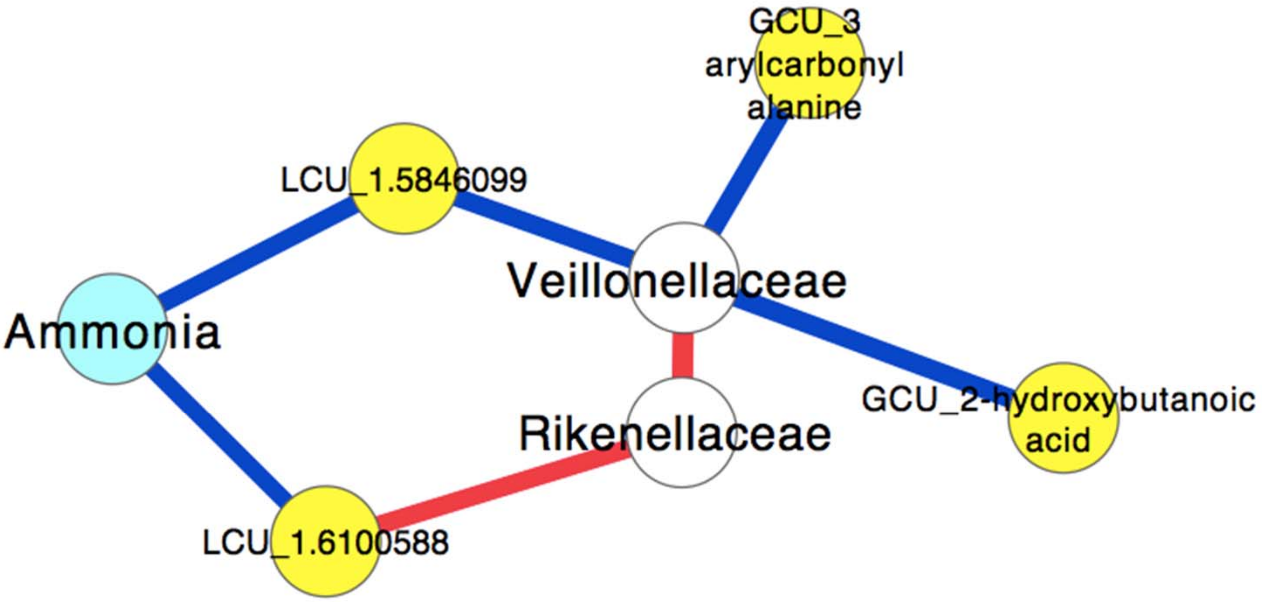

***Veillonellaceae* Before**

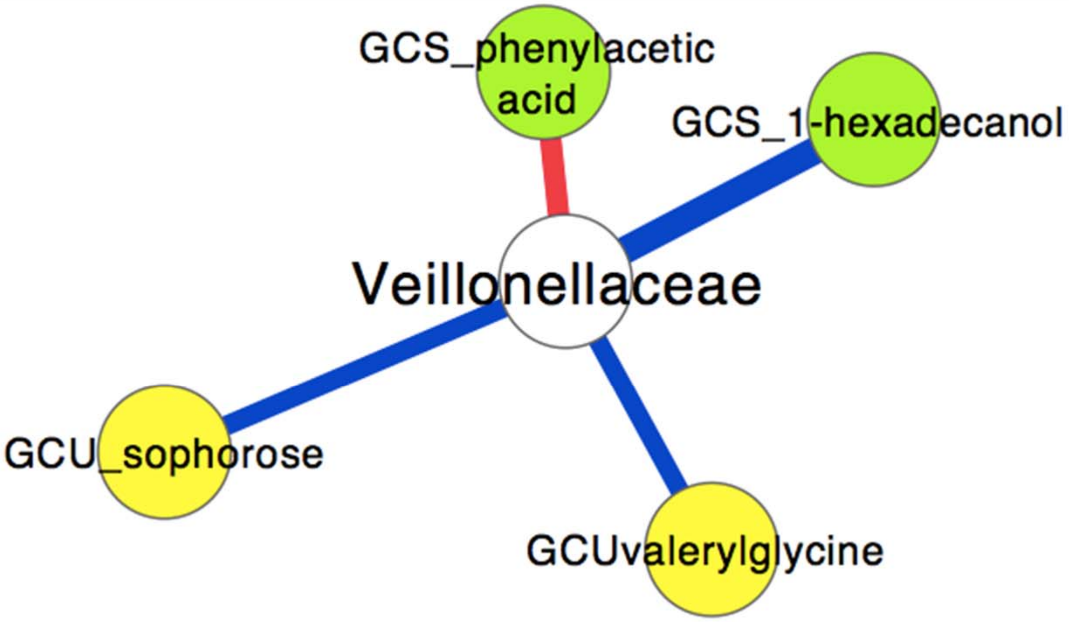

***Veillonellaceae* After**

Figure S7

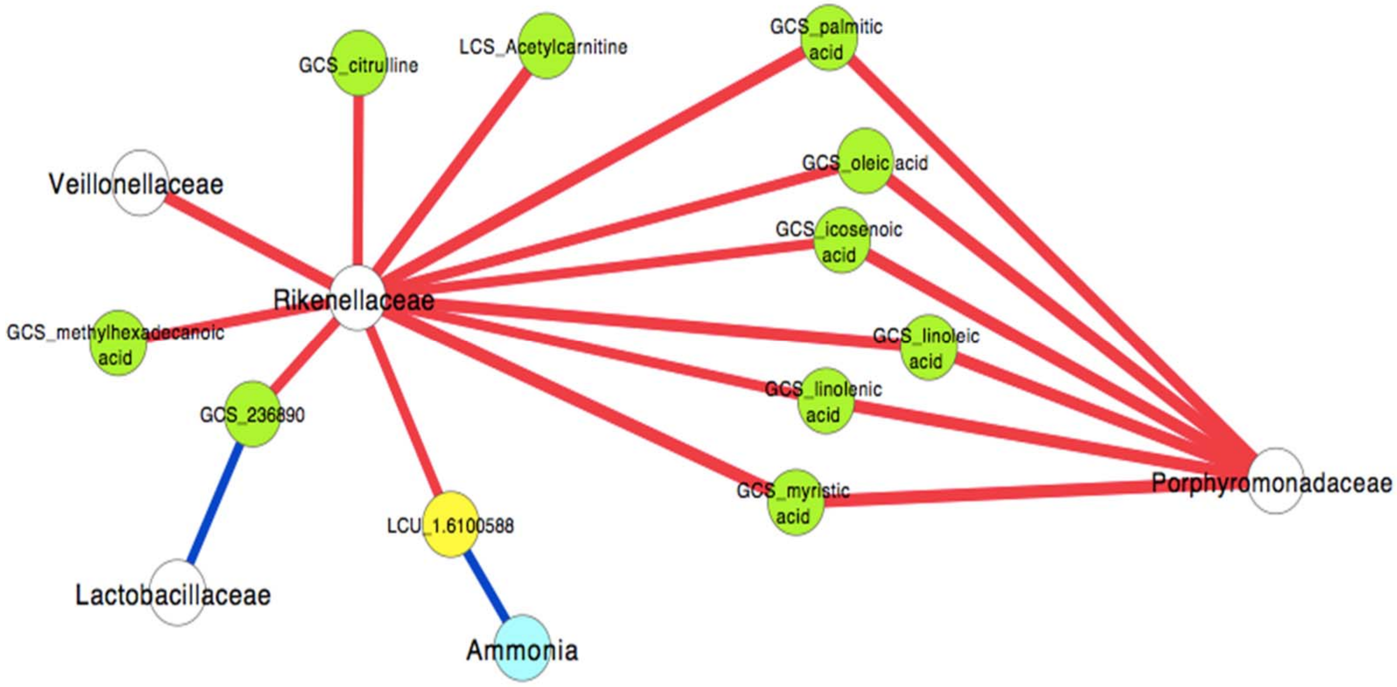

***Rikenellaceae* Before**

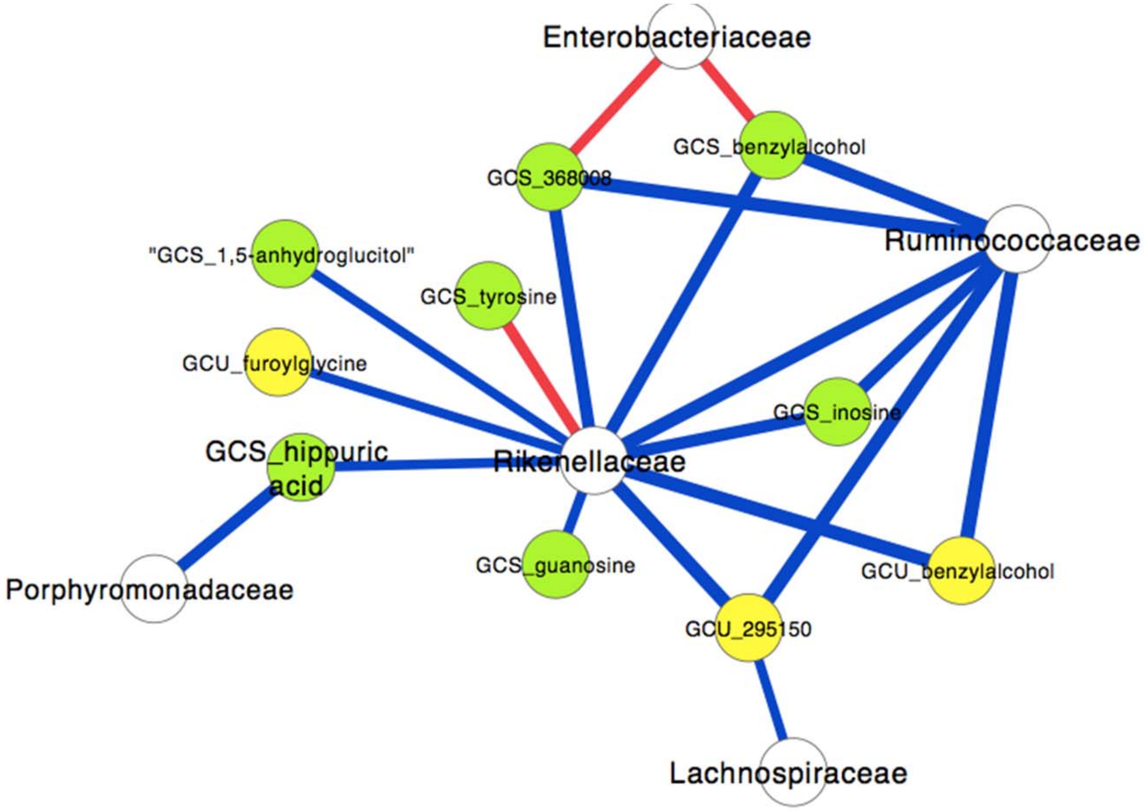

***Rikenellaceae* After**
