# Supplementary material for: Modulation of the Metabiome by Rifaximin in Patients with Cirrhosis and Minimal Hepatic Encephalopathy
Source: PLoS One. 2013 Apr 2;8(4):e60042. doi: 10.1371/journal.pone.0060042 (PMC3615021; doi:10.1371/journal.pone.0060042)
Supplement: Protocol S1 — Trial Protocol (DOC) [file pone.0060042.s006.doc]

**EFFECT OF RIFAXIMIN THERAPY ON BRAIN ACTIVATION IN PATIENTS WITH MINIMAL HEPATIC ENCEPHALOPATHY USING FUNCTIONAL MR, MR SPECTROSCOPY AND DIFFUSION TENSOR IMAGING: A PROSPECTIVE TRIAL**

**Principal Investigator:** Jasmohan S. Bajaj, MD, MSc, GI Section

**Subinvestigators:** Douglas M. Heuman, MD, GI Section

Mitchell L. Schubert MD, GI Section

William M. Pandak MD, GI Section

Juan Diego Baltodano MD, GI Section

HoChong S. Gilles FNP, GI Section

Michael Lennon, MD, Radiology Dept

Vishwadeep Ahluwalia, PhD, GI Section

**Study Location:** Hunter Holmes McGuire VA Medical Center

Richmond, VA

**Protocol No.:**  BAJAJ0010 Amendment # 7

**SPECIFIC AIM**

***Hypothesis:* Rifaximin therapy will result in the improvement of brain edema, reduction in glutamine/glutamate concentration and increase in the brain volume activated on MR assessment in subjects with minimal hepatic encephalopathy.**

This hypothesis will be tested using the following specific aim and 3 sub-aims:

***Specific Aim: To define the effect of rifaximin therapy on functional MRI activation, MR spectroscopic assessment and diffusion weighted imaging evaluation of the brain in subjects with minimal hepatic encephalopathy.***

**Sub-Aim 1**: To define the effect of rifaximin on brain activation induced by the Inhibitory Control Task and N-Back task in functional MRI measurements in subjects with minimal hepatic encephalopathy.

**Sub-Aim 2**: To define the effect of rifaximin on glutamine/glutamate, myoinositol, creatine and n-acetylaspartate levels in the white and gray matter using MR spectroscopy in subjects with minimal hepatic encephalopathy.

**Sub-Aim 3:** To define the effect of rifaximin on brain edema in the entire brain using diffusion-tensor imaging in subjects with minimal hepatic encephalopathy.

**Sub-Aim 4:** To correlate differences in microflora and metabolomice between cirrhotics before and after rifaximin.

**BACKGROUND**

Minimal hepatic encephalopathy (MHE) is a prevalent and significant neuro-cognitive complication of cirrhosis which can adversely affect quality of life, progression to overt hepatic encephalopathy (OHE) and driving skills(1-4). It is considered a pre-clinical stage of OHE and is not diagnosable without specialized testing(5). Small-range studies in MHE subjects have shown that it has a long natural history, which would require a treatment with good efficacy and acceptable adverse effect profile(6).

Subjects with MHE have a characteristic deficit profile which consists of impaired sustained attention, vigilance, response inhibition, processing speed and visuo-motor coordination(5, 7-9). This has led to a specific testing strategy that encompasses testing these specific abilities for diagnosis of MHE. However patho-physiologically, there are several questions that are unanswered regarding MHE and OHE. These questions have been investigated using specialized brain imaging such as structural Magnet Resonance Imaging (MRI), PET and specialized MRI. The specialized MRI is MR spectroscopy, which is a non-invasive assessment of metabolites in specific targeted brain areas, diffusion weighted imaging that measures the movement of water across brain areas and functional MRI (fMRI) that evaluates brain function indirect via detection of local hemodynamic changes –transmitted via neurovascular coupling - in capillaries and draining veins (10, 11).

Brain imaging in subjects with MHE has offered an insight into the pathogenesis of this disease. Similar to OHE, subjects with MHE have increased brain edema, which is vasogenic according to the diffusion-weighted imaging. In addition, subjects with MHE also have an increased accumulation of glutamate and glutamine and reduced myo-inositol in both white and grey matter. This phenomenon is found both in cortical and subcortical structures(12). These studies however have been performed in separate subjects across differing ethnicities and diagnostic criteria for MHE. In addition, there are no studies assessing the effect of therapy on these vital and important brain imaging parameters.

There is considerable controversy regarding the diagnosis of MHE but the Working group on Hepatic encephalopathy has endorsed the use of at least 2 of the following psychometric tests: number connection tests A and B (NCT-A and NCT-B), digit symbol (DST) and block design tests (BDT)(2). The inhibitory control test (ICT) is a computerized test of response inhibition and attention which has shown a high sensitivity for the diagnosis of MHE and is a valid test for this indication(10). It consists of the presentation of a series of letters, interspersed within which are targets, that the subjects are encouraged to respond to and lures, that they are trained not to respond to. The inhibition of response to lures is the crux of this test(7, 13). A unique ability of the ICT is that it can be administered within the MRI scanner, which offers an insight into the patterns of brain activation when mistakes are being made by the subject(10). This sets ICT as a test that can be used to diagnose and characterize MHE both outside and inside the MRI scanner.

The treatment of MHE with lactulose has been shown to improve psychometric performance but owing to the adverse effects associated with this therapy, it is not considered an alternative for long-term therapy(14). Therefore a medical intervention such as rifaximin, will be ideal to study the therapy of MHE, both from a clinical and from a patho-physiological standpoint using the brain imaging.

**IMPORTANCE OF THIS RESEARCH**

**Why is this research important for subjects and the scientific community?**

1. Change in quality of life and psychometric performance: these are essential goals to be achieved in subjects with MHE since they influence every aspect of their life including driving impairment.
2. Tolerability of therapy with rifaximin: compared to the other major therapy of lactulose, which is associated with a high degree of adverse effects that limit compliance, rifaximin is well-tolerated. Since the duration of MHE therapy is likely to be several months to years, it is important for subjects to be treated with an agent with minimal adverse events to encourage compliance.
3. Mechanism of MHE: if this research demonstrates that rifaximin can increase brain activation, decrease brain edema and pro-inflammatory cytokines, it will provide a new insight into the pathogenesis of MHE and will underscore the importance of intestinal based therapies without adverse effects for these subjects.

**PRELIMINARY DATA**

**Feasibility at McGuire DVA Medical Center**:

**Investigator Experience:**

VCUHS and the McGuire VA Medical center have a depth of expertise in hepatology, neuro-radiology and psychology in both the clinical and research aspects. Dr Bajaj, the PI, has made MHE his research focus and has been widely noted as an expert in this field. His co-investigators in the Hepatology Division, including Dr Sanyal and Dr Stravitz are thought-leaders in this field and have a long history of research in hepatology. Dr Bajaj has been working closely with them and has enrolled more than 65 subjects in various MHE-related protocols in the VCUHS and VA over the last 6 months. The neuro-radiologists, Dr Lennon at the VA is an expert in the field of neuroimaging and the hepatology division has a close collaboration with the neuro-radiology division for the study of the effect of liver disease on the brain.

**Investigator experience with rifaximin at the McGuire VA Medical Center:**

Dr Bajaj is currently working on the following investigator-initiated trial “Effect of Rifaximin on Driving Performance, Psychometric Test Performance and Quality of Life in Cirrhotic Subjects With Minimal Hepatic Encephalopathy: A Randomized, Double-Blind, Placebo-Controlled Trial” Dr Bajaj has screened 38 subjects and has enrolled 28 subjects so far in this trial. This trial is also based at the McGuire VA Medical Center and demonstrates the feasibility of performing this research here.

**Equipment and study personnel:**

The VA Medical Center has a 3 Tesla and a 1.5 Tesla magnet to apply fMRI, DTI and MRS techniques. Dr Bajaj and Dr Lennon (previously Dr Damiano) have been working closely on two MRI-related protocols (not related to any medications), the results of which are presented in the preliminary data.

Dr Bajaj has three research coordinators at VCU and one at the VA Medical Center. These coordinators have been trained by Dr. Bajaj in questionnaire administration, administration of psychometric tests and are competent to perform these duties. Dr Bajaj also has access to the VA Medical Center Investigational Pharmacy for storage and to an experienced research phlebotomistwho is invaluable in collection, processing and storage of blood samples from subjects.

**MR Spectroscopic analysis of cirrhosis and HE in the brain:** Preliminary results from BAJAJ003 (MIRB ID #01478), Is Continuous Lactulose Therapy Necessary for Subjects with Hepatic Encephalopathy? A Prospective Study of Controlled Lactulose Withdrawal

6 subjects with cirrhosis on lactulose therapy underwent MRS and DTI imaging. They were compared to 4 age-matched healthy controls. Lactulose therapy was withdrawn from all cirrhotics and they underwent MRS 14 days after lactulose withdrawal.

|  | **Glu/Gln** | | **mI** | | **GPC** | | **Cr** | | **NAA/NAAG** | |
| --- | --- | --- | --- | --- | --- | --- | --- | --- | --- | --- |
|  | RPWM | OCG | RPWM | OCG | RPWM | OCG | RPWM | OCG | RPWM | OCG |
| Normal | 7.35 | 8.4 | 4.65 | 4.49 | 1.4 | 0.93 | 4.56 | 5.26 | 9.51 | 8.18 |
| On lactulose | 11.48* | 10.7* | 0.93* | 2.13* | 1.21 | 0.89 | 3.73 | 5.36 | 8.56 | 7.79 |
| Without lactulose | 15.08 | 18.3 | 0.72 | 1.81 | 1.17 | 0.96 | 4.22 | 5.66 | 9.11 | 8.43 |
| Glu/Gln: glutamate/glutamine, mI: myoinositol, GPC: Choline, Cr: Creatine, NAA/NAAG:  RPWM: right parietal white matter, OCG: Occipital gyrus grey matter  *: significantly different compared to baseline and to controls. | | | | | | | | | | |

**RESEARCH DESIGN AND METHODS**

**This will be a prospective clinical trial of the effect of rifaximin on brain activation and psychometric function which will be performed in subjects with minimal hepatic encephalopathy.**

**Subject Recruitment:**

All subjects for this study will be recruited from the McGuire DVAMC or VCU Medical Center in Richmond, VA. A written informed consent will be obtained from each subject before enrollment.

All prospective, interested subjects will undergo a screening visit in which the inclusion and exclusion criteria will be confirmed. Screening visits will be conducted by Dr Bajaj and his research coordinators.

All subjects who successfully screen will be included in the study. Subjects determined ineligible following screening will be paid $25 for their time and their participation in the study will be complete. Based on previous recruitment outcomes, it is anticipated that 85% of screened subjects will be eligible to participate.

**Inclusion Criteria for Cirrhotics:**

- Age 18-65 years
- Cirrhosis diagnosed clinically or by biopsy
- Minimal hepatic encephalopathy defined by impaired performance on at least 2 of the following: NCT-A, NCT-B, DST and BDT compared to age and education-matched controls.
- No contraindication to MRI
- No TIPS procedure or elective surgery planned within the next 8 weeks—

**Exclusion Criteria for Cirrhotics:**

- Current therapy with lactulose, rifaximin or other treatment for hepatic encephalopathy.
- Prior episodes of overt HE
- Mini-Mental Status Evaluation (MMSE) score <25
- TIPS placement
- Current therapy with opioids, anti-depressants, anti-seizure medications or anti-psychotic medications
- Unable to give informed consent.
- Pregnancy

**Screening Visit Procedures for Cirrhotics:**

1. Obtain informed consent
2. Ability to undergo MRI will be determined using the MRI safety form.
3. Review of medical history: demographic variables including age and gender, date of diagnosis of cirrhosis and etiology of cirrhosis, prior history of and date of complications of cirrhosis such as variceal bleeding, spontaneous bacterial peritonitis and hepatocellular carcinoma, endoscopy reports to evaluate for presence of esophageal/gastric varices and concurrent medical problems, especially diabetes mellitus.
4. Physical and neurological examination by Dr Bajaj to exclude overt HE and to confirm right-handedness.
5. Review of medications to exclude those on prohibited medications.
6. MMSE (a score > 25 will qualify the subject for the study).
7. PHES. NCT-A, NCT-B, BDT, SIP and DST administration.
8. Urine sample collection for pregnancy testing for women of child-bearing potential.

**Study Visit 1 Procedures for Cirrhotics:**

1. Current medications and compliance will be noted.
2. Sickness impact profile (SIP), a questionnaire inquiring about quality of life will be administered.
3. PHES,ICT, NCT-A, NCT-B, BDT and DST will be administered.
4. Blood draw for MELD calculation (a logarithmic score of INR, serum bilirubin and serum
5. creatinine), complete blood count, basic metabolic panel and liver function tests, cytokine and bile acid assessment, neuroglial injury marker assessment and proteomics.
6. Stool collection for testing of microbial population. Bile acid analysis will also be conducted on stool samples.
7. Urine sample collection for metabolomics and pregnancy testing for women of child-bearing potential.
8. MRI scans:

1 fMRI

2. MR spectroscopy

3. DTI

1. Study drug (rifaximin) will be dispensed to cirrhotic subjects at Visit 1. Subjects will be instructed to take the study drug (one 550mg tablet PO BID) for 8 weeks.
2. Medication and symptom logs will be given to subjects to be used to record study drug compliance and any symptoms experienced for the next 8 weeks.

*Cirrhotic subjects may undergo Screening and Visit 1 on the same day for their convenience. Should this occur, subjects will be asked to complete the ICT, NCT-A, NCT-B, BDT and DST only once.*

**Study Visit 2 for Cirrhotics: (Week 8 or Final Study Visit)**

The following will be performed:

1. Review of medication logs to assess compliance.
2. Review of symptom log.
3. Sickness impact profile (SIP), a questionnaire inquiring about quality of life
4. PHES, ICT, NCT-A, NCT-B, BDT and DST administration.
5. Blood draw for MELD calculation, specialized cytokine and bile acid assessment, neuroglial injury marker assessment and proteomics.
6. Urine sample collection for metabolomics and pregnancy testing for women of child-bearing potential.
7. Stool sample collection for microbial analysis. Bile acid analysis will also be

conducted on stool samples.

1. MRI scans:
2. fMRI with ICT and N-back task
3. MR spectroscopy
4. DTI

**Early Termination / Early Withdrawal**

Subjects withdrawn from the study prior to completing Study Visit 2 will be asked to undergo Study Visit 2 procedures if feasible.

**Laboratory Analysis:**

Routine blood/urine tests will be drawn and analyzed locally at McGuire DVAMC.

**Specialized Blood Testing:**

Blood samples will be collected by the GI Research phlebotomist, centrifuged and stored for analysis of the following cytokines and metabolomics:

1. Endotoxin
2. Metabolomics

**Cytokine and Biomarker Analysis:**

De-identified serum samples will be sent to Assay Gate Inc, Suite 103, Ijamsville, MD for independent analysis of cytokines. These samples will be destroyed after analysis is complete.

**Specialized Urine / Stool Testing:**

1. Urine sample for metabolomics
2. Stool sample for microbial population and bile acid analysis.

Specialized test samples will be sent to Dr. Oliver Fiehn at UC Davis , Dr. Philip Hylemon at Virginia Commonwealth University, to Dr. Patrick Gillevet at George Mason University, Manassas, VirginiaThe specialized test samples listed above will be de-identified and destroyed after analysis is completed.

Costs incurred for processing and shipping of samples for specialized testing will be paid by Dr. Bajaj, the principal investigator.

**Storage of Blood, Urine and Stool Samples:**

Subjects will be specifically asked in the consent form to allow storage of a portion of their specialized test samples for future analysis. If subjects agree, a portion of their blood, urine and stool samples will be stored in Dr. William M. Pandak’s research laboratory on the 3rd Floor of McGuire DVAMC until future studies are performed. Bile acid analysis of de-identified stool samples will be conducted by Dr. Pandak’s laboratory staff prior to sample storage. Subjects not agreeing to storage of their samples for future analysis can still participate in the study but all of their samples will be destroyed upon completion of analysis for this study.

**Study Drug:**

Rifaximin 550mg tablets will be supplied by Salix Pharmaceuticals, Inc. and stored and dispensed through the Investigational Pharmacy at McGuire DVAMC.

**Psychometric Test Descriptions:**

SIP (Sickness Impact Profile): A quality of life questionnaire (pencil/paper).

ICT (Inhibitory Control Test): A test of memory and the ability to inhibit a response (computerized).

DST (Digit Symbol Test): A test of reaction time and psychomotor speed (pencil/paper).

BDT (Block Design Test): A test of intelligence and visuo-motor coordination (pencil/paper).

NCT-A (Number Connection Test A): A test of reaction time (pencil/paper).

NCT-B (Number Connection Test B): A test of reaction time (pencil/paper).

**Sample Size:** Based on our preliminary data, we were able to find strong differences in brain activation, volumes and ROI in subjects with MHE compared to healthy controls. We expect a sample size of 15 subjects (compared to their state before rifaximin) to be an adequate group given the strength of the longitudinal design.

**Primary Statistical Analysis:** Response to lure inhibitions before and after rifaximin therapy on fMRI

fMRI analysis will be performed using:

**a) Deconvolution analysis of fMRI hemodynamic response**

**b) Voxel-Wise Analysis**

**c) Region of Interest (ROI) analysis**

Similar analyses will be performed for the N-back task.

DTI analysis will study the change in brain water content in specific gray and white matter areas in both states.

MRS will study the changes in metabolite concentrations in pre-specified gray and white matter areas in both states.

**Other Outcomes**:

- 1. Psychometric test performance
  2. MELD score
  3. Sickness Impact Profile
  4. Cytokine and neuroglial injury marker change
  5. Microbiome analysis
  6. metabolomics

**All of the above will be compared before and after rifaximin using paired t-tests, metastats, PLS-DA and other parametric microbiome tests .**

**It is anticipated that subjects will increase their brain volume and activation intensity while successfully inhibiting from lures, to increase activation during the N-back task, to reduce the brain edema and decrease the glutamine/glutamate concentrations in the white and gray matter after rifaximin therapy. It is also anticipated that rifaximin will improve psychometric performance, MELD score and SIP and to reduce the inflammatory cytokine milieu by altering the microbiome and metabolome.**

**Monitoring & Reporting of Adverse Events:** Subjects will be monitored closely per protocol at all visits for adverse events, All subjects will be provided with a 24 hr. phone # for investigators which they can use to obtain assistance during the study. Unscheduled visits may be necessary for subject evaluation, particularly, if a subject has an adverse event. Subjects experiencing adverse events will be monitored with relevant clinical assessments and laboratory tests as determined by the Investigator. All adverse events will be followed to satisfactory resolution or stabilization of the event(s). Actions taken and follow-up results will be recorded on the appropriate page of the case report form, as well as in the subject's source documentation. Follow-up laboratory results will be filed with the subject's source documentation. All Serious Adverse Events (SAEs) will be reported to Dr. Bajaj within 24 hours of the study staff becoming aware of the event. All SAEs will subsequently be reported to McGuire IRB. Serious Adverse Events are defined as any untoward medical occurrence that:

- Results in death.
- Is life-threatening.
- Requires in-subject hospitalization or prolongation of existing hospitalization.
- Results in persistent or significant disability or incapacity.
- Is a congenital anomaly or birth defect.

**Pregnancy:** Medically accepted birth control will be required to enter this study. For females, this may include, but is not limited to, birth control pills, IUD’s, condoms, diaphragms, implants, being surgically sterile, or being in a post menopausal state. Females of childbearing age, must have a negative pregnancy test before entering the study. Additional pregnancy tests will be done at Visit 1 & Visit 2 and Early Termination. Subjects that become pregnant while taking part in this study will be required to notify Dr. Bajaj immediately. Males participating in this study, must agree not to have sexual intercourse, or agree to use barrier methods of birth control (condom with spermicidal gel) while in the study. Should the spouse/partner of a male participant become pregnant during the study, Dr. Bajaj should be notified immediately. Both male and female participants must use birth control for 2 months after stopping study drug. Should pregnancy occur during this 2 month post-study period, Dr. Bajaj should be notified immediately.

**Ethical Considerations:**

This study will be conducted according to good clinical practice guidelines, applicable laws and

regulations. Significant findings that could affect the safety and welfare of research subjects will be reported to Salix Pharmaeuticals, Inc., and McGuire Institutional Review Board.

**HUMAN RESEARCH SUBJECTS PROTECTION**

**Potential risks to subjects:**

**Physical:**

- Related to drug: 8-9% chance of minor GI upset and allergic reactions
- MRI-related risks: low risk of claustrophobia and ferromagnetic reaction due to implants.
- Development of OHE

**Psychological:**

- Small risk of anxiety regarding test performance

**Measures To Protect Against Risks:**

- A written informed consent will be obtained.
- Subjects will be given a 24 hour number to contact us regarding any adverse events
- MRI safety form will be completed at screening to avoid possible adverse events
- All study materials will be stored in a locked cabinet in Dr Bajaj’s office and only the study team will have access to it.

**References:**

1. Ortiz M, Jacas C, Cordoba J. Minimal hepatic encephalopathy: diagnosis, clinical significance and recommendations. J Hepatol 2005;42 Suppl:S45-53.

2. Ferenci P, Lockwood A, Mullen K, et al. Hepatic encephalopathy--definition, nomenclature, diagnosis, and quantification: final report of the working party at the 11th World Congresses of Gastroenterology, Vienna, 1998. Hepatology 2002;35:716-21.

3. Bajaj JS, Hafeezullah M, Hoffmann RG, et al. Navigation skill impairment: Another dimension of the driving difficulties in minimal hepatic encephalopathy. Hepatology 2008;47:596-604.

4. Bustamante J, Rimola A, Ventura PJ, et al. Prognostic significance of hepatic encephalopathy in subjects with cirrhosis. J Hepatol 1999;30:890-5.

5. Weissenborn K, Ennen JC, Schomerus H, et al. Neuropsychological characterization of hepatic encephalopathy. J Hepatol 2001;34:768-73.

6. Das A, Dhiman RK, Saraswat VA, et al. Prevalence and natural history of subclinical hepatic encephalopathy in cirrhosis. J Gastroenterol Hepatol 2001;16:531-5.

7. Bajaj JS, Hafeezullah M, Franco J, et al. Inhibitory Control Test for the Diagnosis of Minimal Hepatic Encephalopathy. Gastroenterology 2008.

8. Weissenborn K, Giewekemeyer K, Heidenreich S, et al. Attention, memory, and cognitive function in hepatic encephalopathy. Metab Brain Dis 2005;20:359-67.

9. Weissenborn K, Heidenreich S, Ennen J, et al. Attention deficits in minimal hepatic encephalopathy. Metab Brain Dis 2001;16:13-9.

10. Garavan H, Ross TJ, Stein EA. Right hemispheric dominance of inhibitory control: an event-related functional MRI study. Proc Natl Acad Sci U S A 1999;96:8301-6.

11. Zafiris O, Kircheis G, Rood HA, et al. Neural mechanism underlying impaired visual judgement in the dysmetabolic brain: an fMRI study. Neuroimage 2004;22:541-52.

12. Rovira A, Alonso J, Cordoba J. MR imaging findings in hepatic encephalopathy. AJNR Am J Neuroradiol 2008;29:1612-21.

13. Bajaj JS, Saeian K, Verber MD, et al. Inhibitory control test is a simple method to diagnose minimal hepatic encephalopathy and predict development of overt hepatic encephalopathy. Am J Gastroenterol 2007;102:754-60.

14. Prasad S, Dhiman RK, Duseja A, et al. Lactulose improves cognitive functions and health-related quality of life in subjects with cirrhosis who have minimal hepatic encephalopathy. Hepatology 2007;45:549-559.
