# Supplementary material for: Modulation of the Metabiome by Rifaximin in Patients with Cirrhosis and Minimal Hepatic Encephalopathy
Source: PLoS One. 2013 Apr 2;8(4):e60042. doi: 10.1371/journal.pone.0060042 (PMC3615021; doi:10.1371/journal.pone.0060042)
Supplement: Listing S1 — Clinical trials registration. (PDF) [file pone.0060042.s008.pdf]

Protocol Registration Receipt
12/10/2012

Grantor: CDER   IND/IDE Number: 77,783   Serial Number: 1

Study of Rifaximin in Minimal Hepatic Encephalopathy

This study has been completed.

Sponsor:	Hunter Holmes Mcguire Veteran Affairs Medical Center
Collaborators:	Salix Pharmaceuticals
Information provided by (Responsible Party):	Jasmohan Bajaj, Hunter Holmes Mcguire Veteran Affairs Medical Center
ClinicalTrials.gov Identifier:	NCT01069133

Purpose

Rifaximin therapy will improve brain functioning on MRI scanning and change the microbiome and metabolome.

Condition	Intervention	Phase
Minimal Hepatic Encephalopathy	Drug: Rifaximin<br>Drug: rifaximin	Phase 1/Phase 2

Study Type: Interventional
Study Design: Treatment, Single Group Assignment, Open Label, Non-Randomized
Official Title: Effect of Rifaximin Therapy on Brain Activation in Patients With Minimal Hepatic Encephalopathy Using Functional MR, MR Spectroscopy,Diffusion Tensor Imaging Microbiome and Metabolome: a Prospective Trial

Further study details as provided by Jasmohan Bajaj, Hunter Holmes Mcguire Veteran Affairs Medical Center:

Primary Outcome Measure:

- brain activation on fMRI [Time Frame: 2 months] [Designated as safety issue: No]
- Microbiome constituents [Time Frame: 8 weeks] [Designated as safety issue: No]

Secondary Outcome Measures:

- brain edema and brain metabolite concentration [Time Frame: 2 months] [Designated as safety issue: No]
- Metabolome of urine and serum [Time Frame: 8 weeks] [Designated as safety issue: No]

Enrollment: 20
Study Start Date: February 2010
Study Completion Date: December 2012
Primary Completion Date: May 2012

Arms	Assigned Interventions
Experimental: Rifaximin	Drug: Rifaximin<br>550mg BID open-label<br>Other Names:<br>xifaxan<br>Drug: rifaximin<br>550mg PO BID<br>Other Names:<br>xifaxan

Eligibility

Ages Eligible for Study: 18 Years to 65 Years
Genders Eligible for Study: Both

Inclusion Criteria:

- Age 18-65 years
- cirrhosis diagnosed by clinical or biopsy grounds
- Minimal hepatic encephalopathy defined by impaired performance on at least 2 of the following: number connection tests A/B, digit symbol and block design tests (NCT-A, NCT-B, DST and BDT) compared to age and education-matched controls.
- No contraindications to MRI
- TIPS (transjugular intra-hepatic porto-systemic shunt) procedure or elective surgery planned within the next 8 weeks

Exclusion Criteria:

- Current therapy with lactulose, rifaximin or other treatment for hepatic encephalopathy.
- Prior episodes of overt HE
- MMSE <25
- TIPS placement
- Unable to give informed consent.

- Contra-indications to MRI

Contacts and Locations

Locations

United States, Virginia

Hunter Holmes McGuire VA Medical Center
Richmond, Virginia, United States, 23249

Investigators

Principal Investigator: Jasmohan S Bajaj, MD, MSc McGuire VA Medical Center

More Information

Responsible Party:	Jasmohan Bajaj, Associate Professor of Medicine, Hunter Holmes Mcguire Veteran Affairs Medical Center
Study ID Numbers:	BAJAJ010
Health Authority:	United States: Food and Drug Administration
